# Proanthocyanidins enhance antitumor immunity by promoting ubiquitin-proteasomal PD-L1 degradation via stabilization of LKB1 and SYVN1

**DOI:** 10.1172/JCI197592

**Published:** 2026-02-02

**Authors:** Mengting Xu, Xuwen Lin, Hanchi Xu, Hongmei Hu, Xinying Xue, Qing Zhang, Dianping Yu, Saisai Tian, Mei Xie, Linyang Li, Xiaoyu Tao, Xinru Li, Simeng Li, Shize Xie, Yating Tian, Xia Liu, Hanchen Xu, Qun Wang, Weidong Zhang, Sanhong Liu

**Affiliations:** 1 Institute of Digestive Diseases, Longhua Hospital, Shanghai University of Traditional Chinese Medicine, Shanghai, China.; 2 State Key Laboratory of Discovery and Utilization of Functional Components in Traditional Chinese Medicine, Shanghai Frontiers Science Center of TCM Chemical Biology, Institute of Interdisciplinary Integrative Medicine Research, Shanghai University of Traditional Chinese Medicine, Shanghai, China.; 3 Department of Respiratory and Critical Care, Xuanwu Hospital, Capital Medical University, Beijing, China.; 4 School of Pharmacy, Second Military Medical University, Shanghai, China.; 5 Institute of Medicinal Plant Development, Chinese Academy of Medical Sciences and Peking Union Medical College, Beijing, China.

**Keywords:** Immunology, Oncology, Cancer immunotherapy

## Abstract

Programmed cell death 1 ligand 1–targeted (PD-L1–targeted) immune checkpoint inhibitors are revolutionizing cancer therapy. However, strategies to induce endogenous PD-L1 degradation represent an emerging therapeutic paradigm. Here, we identified proanthocyanidins (PC) as a potent inducer of PD-L1 degradation through an endoplasmic reticulum–associated degradation (ERAD) mechanism. Mechanistically, PC exerted dual effects: First, it targeted and stabilized LKB1 to activate AMPK in tumor cells, subsequently inducing the phosphorylation of PD-L1 at Ser195 — a disruption that in turn impaired glycosylation of PD-L1 and promoted its retention in the ER. Second, PC directly bound to the E3 ubiquitin ligase SYVN1 to increase its protein stability, which strengthened PD-L1–SYVN1 binding, thereby accelerating K48-linked ubiquitination and proteasomal degradation of ER-retained PD-L1. This cascade culminated in the activation of CD8^+^ T cell–dominated antitumor immune responses, accompanied by suppression of myeloid-derived suppressor cells and regulatory T cells. In preclinical models of lung and colorectal cancer, PC exhibited synergistic antitumor efficacy when combined with anti–cytotoxic T lymphocyte antigen 4 (anti–CTLA-4) antibodies. Notably, PC also potently inhibited the progression of azoxymethane/dextran sodium sulfate–induced orthotopic colorectal cancer in mice. Collectively, our findings unveil an antitumor mechanism of PC, establishing this small-molecule compound as an ERAD pathway–exploiting immune checkpoint modulator with promising translational potential for cancer therapy.

## Abstract 2

Proanthocyanidins promote PD-L1 degradation via SYVN1 to enhance antitumor immunity.

## Graphical abstract

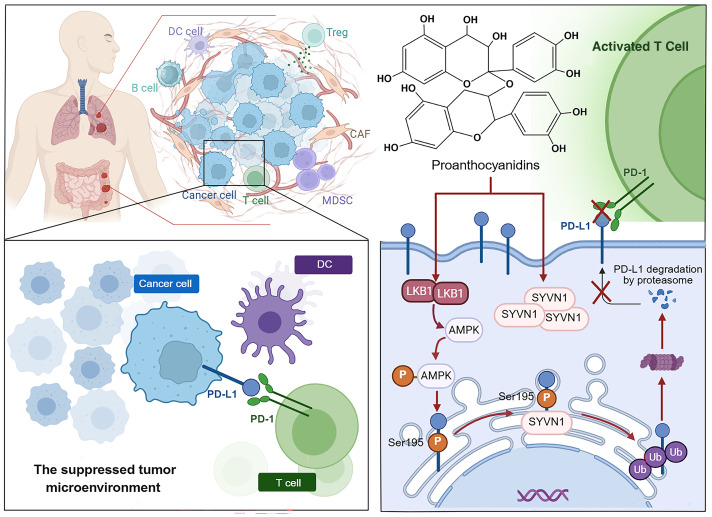

## Introduction

In the landscape of cancer treatment, immune checkpoint inhibitors (ICIs) that target programmed cell death 1 (PD-1), programmed cell death 1 ligand 1 (PD-L1), or cytotoxic T lymphocyte antigen 4 (CTLA-4) have emerged as a revolutionary force in cancer immunotherapy in recent years. These therapies have substantially enhanced the clinical outcomes for patients with diverse malignancies by reactivating antitumor immune responses through the blockade of T cell inhibitory signals, thus becoming a pillar in oncology treatment (1–3).

However, a growing body of clinical evidence has illuminated the limitations of ICIs. A substantial number of patients experience either primary or acquired resistance to immune checkpoint blockade. The underlying mechanisms are multifaceted, encompassing sustained PD-L1 expression, compensatory upregulation of alternative immune checkpoints, and the recruitment of immunosuppressive cell populations within the tumor microenvironment (4). This resistance has spurred a quest for alternative therapeutic strategies, particularly among small-molecule inhibitors, as the understanding of immune checkpoint regulatory mechanisms deepens (5, 6).

Small-molecule inhibitors present several pharmacological advantages over monoclonal antibodies. They offer superior tissue penetration, oral bioavailability, and the potential for combination therapies, which could overcome the limitations of antibody-based approaches (7). Currently, several small-molecule inhibitors targeting the PD-1/PD-L1 axis are under clinical investigation. For instance, CA-170 binds to PD-L1 and induces conformational changes that disrupt PD-1/PD-L1 interactions (8, 9), while INCB086550 promotes PD-L1 dimerization and internalization, thereby reducing its cell surface expression (10). Nevertheless, challenges such as limited target specificity and inconsistent clinical response rates continue to hamper their widespread success, fueling the search for next-generation inhibitors with improved therapeutic profiles.

The role of posttranslational modifications in regulating PD-L1 expression and function has been a focal point of emerging research. A complex network of phosphorylation, ubiquitination, and glycosylation events dynamically governs PD-L1 stability and its interaction with immune cells (11, 12). In EGFR-driven tumors, for example, GSK3β-mediated phosphorylation of PD-L1 enables its recognition by the β-TrCP E3 ubiquitin ligase complex, modulating protein turnover (11, 13). The recent discovery of PD-L1 acetylation and its regulatory enzymes has further expanded the pool of potential therapeutic targets (14). These advancements not only provide a more profound understanding of the epigenetic regulation of immune checkpoints but also open up new avenues for the development of small-molecule drugs that target “epi-immune” modulation, offering innovative strategies to overcome clinical resistance. Proanthocyanidins (PC), a natural oligomeric flavonoid abundantly present in grape seeds, has been known for its broad-spectrum bioactivities, including antioxidant and antiinflammatory effects (15). Recent studies have indicated its inhibitory effect on reflux-induced esophageal adenocarcinoma (16). However, the molecular mechanisms of PC, especially its role in modulating immune checkpoints, remain largely uncharted territory.

In this study, through a systematic screening of a series of natural small-molecule compounds, we identified that PC substantially reduced PD-L1 expression on cancer cells. Our mechanistic investigations revealed that PC promotes PD-L1 degradation and obstructs immunosuppressive signaling by targeting LKB1 to facilitate AMPK phosphorylation, thereby inducing the phosphorylation of PD-L1 at Ser195, which disrupts the normal glycosylation of PD-L1, resulting in its retention in the ER. Concurrently, PC directly interacts with synoviolin 1 (SYVN1) to increase its protein stability, which strengthens PD-L1–SYVN1 binding, driving K48-linked ubiquitination and proteasomal degradation of ER-retained PD-L1. We further evaluated the combination of PC with immunotherapy, and our results demonstrated a notable enhancement in therapeutic efficacy, suggesting that this combination may serve as an effective strategy for cancer treatment. These findings not only shed light on a mechanism of PC in modulating immune checkpoints but also hold promise for the development of cancer immunotherapy approaches.

## Results

### PC demonstrates potent broad-spectrum capability to downregulate PD-L1 expression in tumor cells.

**Figure 1:**
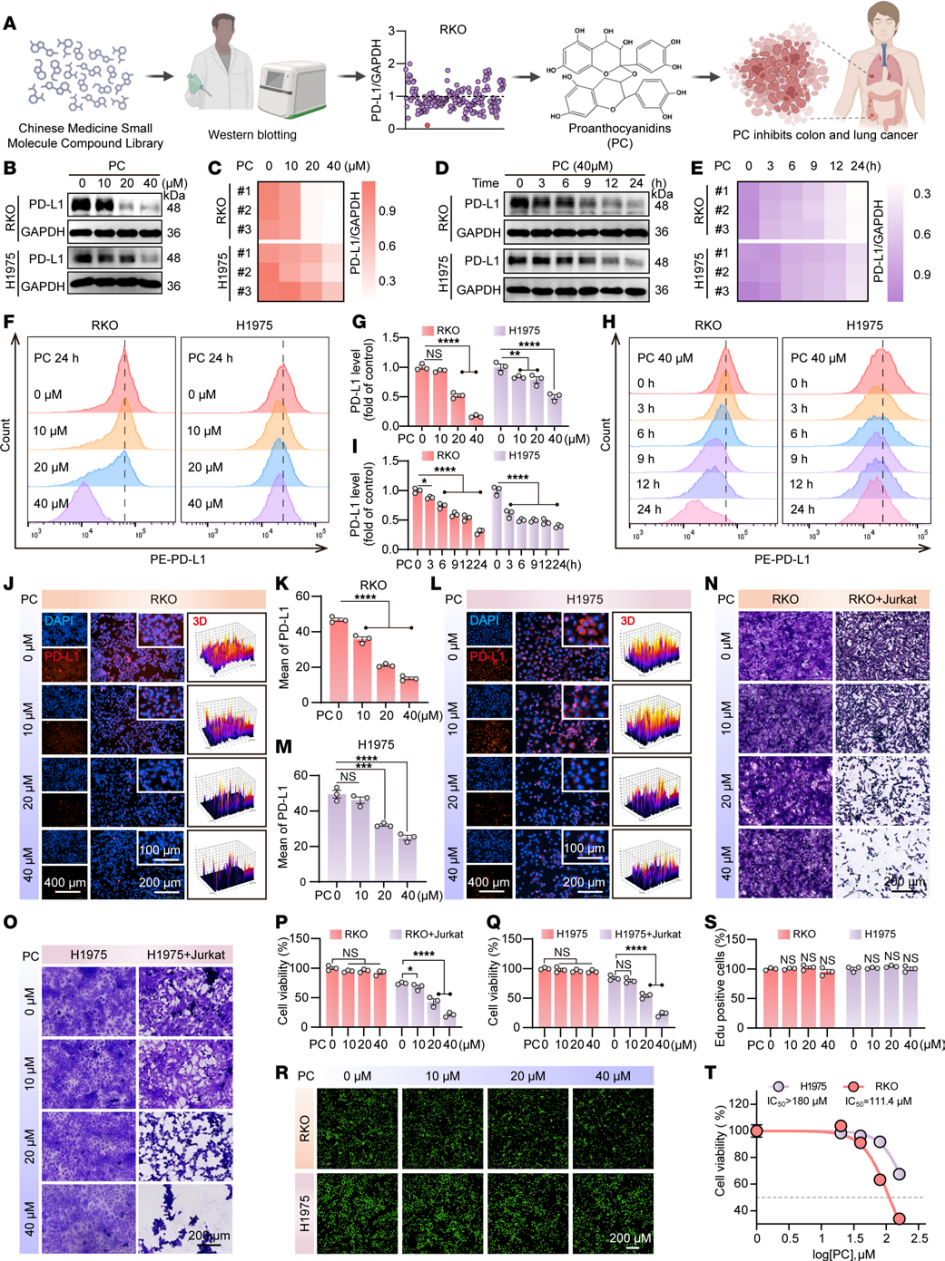
PC enhances T cell killing by downregulating PD-L1. (**A**) PC was identified as a PD-L1 inhibitor from a screen of 209 natural molecules using Western blotting. (**B**) Total PD-L1 protein levels were determined by Western blotting analysis in RKO and H1975 cells following exposure to PC at concentrations of 0 μM, 10 μM, 20 μM, and 40 μM for 24 hours. (**C**) Quantification of **B**. (**D**) Western blotting analysis was performed to assess total PD-L1 protein expression in RKO and H1975 cells treated with 40 μM PC for various time intervals. (**E**) Quantification of **D**. (**F**–**I**) Membrane PD-L1 levels were measured by flow cytometry after treatment with varying PC concentrations (**F**) or durations (**H**), with quantifications in **G** and **I**. (**J**–**M**) Immunofluorescence detected membrane PD-L1 in RKO (**J**) and H1975 (**L**) cells after PC treatment (nuclei stained with DAPI; scale bar: 200 μm). Quantifications are in **K** and **M**. (**N**–**Q**) Pretreated RKO (9 hours) (**N**) and H1975 (24 hours) (**O**) cells were cocultured with PD-1–overexpressing Jurkat T cells for 24 hours at an effector-to-target ratio of 9:1. Prior to coculture, the Jurkat T cells were activated with phytohemagglutinin (PHA) (1 mg/mL) and PMA (50 ng/mL). The survival of target cells was quantified using crystal violet staining. Quantitative data from **N** and **O** are presented in **P** and **Q**, respectively. (**R** and **S**) Cytotoxicity of various PC concentrations on RKO and H1975 cells was assessed by EdU assay and the corresponding quantitation. (**T**) CCK-8 assay for cytotoxicity under the same conditions. The data shown are the mean ± SEM of triplicate experiments. Statistical differences were determined by 1-way ANOVA with Dunnett’s multiple-comparison test. *P < 0.05, **P < 0.01, ***P < 0.001, and ****P < 0.0001.

To identify small-molecule compounds that can substantially downregulate PD-L1, we conducted pharmacological screening of 209 natural small-molecule compounds (Selleck) using the RKO cell line, which has high PD-L1 expression. Among the tested compounds, PC resulted in the most substantial PD-L1 downregulation (Figure 1A). To systematically evaluate the broad-spectrum efficacy of PC-mediated PD-L1 downregulation, flow cytometric quantification across multiple cancer lineages, including HCT116 (colorectal), HT29 (colon), MC38 (murine colorectal), and H460 (non–small cell lung cancer, NSCLC) cells consistently revealed an inverse correlation between PC concentration and cell surface PD-L1 expression levels (Supplemental Figure 1, A–D; supplemental material available online with this article; https://doi.org/10.1172/JCI197592DS1). To further explore the effects of PC on the RKO and H1975 cell lines, experiments were conducted in which these cell lines were treated with gradient concentrations of PC (ranging from 0 to 40 μM) or exposed to 40 μM PC for different durations (from 0 to 24 hours). As shown in Figure 1, B–I, PC markedly inhibited total PD-L1 protein expression in both cell lines in a concentration- and time-dependent manner while decreasing PD-L1 membrane surface levels. Importantly, immunofluorescence (IF) assays further confirmed this regulatory effect (Figure 1, J–M, and Supplemental Figure 1, E–H).

Subsequently, to investigate whether PC enhances T cell–mediated cytotoxicity against tumor cells by downregulating PD-L1, PD-1–overexpressing Jurkat cells were subsequently cocultured with RKO and H1975 cells treated with different concentrations of PC. As shown in Figure 1, N–Q, the cytotoxicity of Jurkat cells to tumor cells was substantially increased with increasing PC concentration. Primary T cell killing exhibited identical results (Supplemental Figure 1, I–L). Moreover, 5-ethynyl-2′-deoxyuridine (EdU) (Figure 1, R and S) and cell counting kit-8 (CCK-8) (Figure 1T) assays demonstrated that PC exhibited minimal cytotoxicity in its effective concentration range. In conclusion, PC is a safe and broad-spectrum inhibitor of PD-L1 with potential applications in enhancing T cell–mediated antitumor activity.

### PC mediates antitumor immunity by inhibiting PD-L1 expression in vivo.

**Figure 2:**
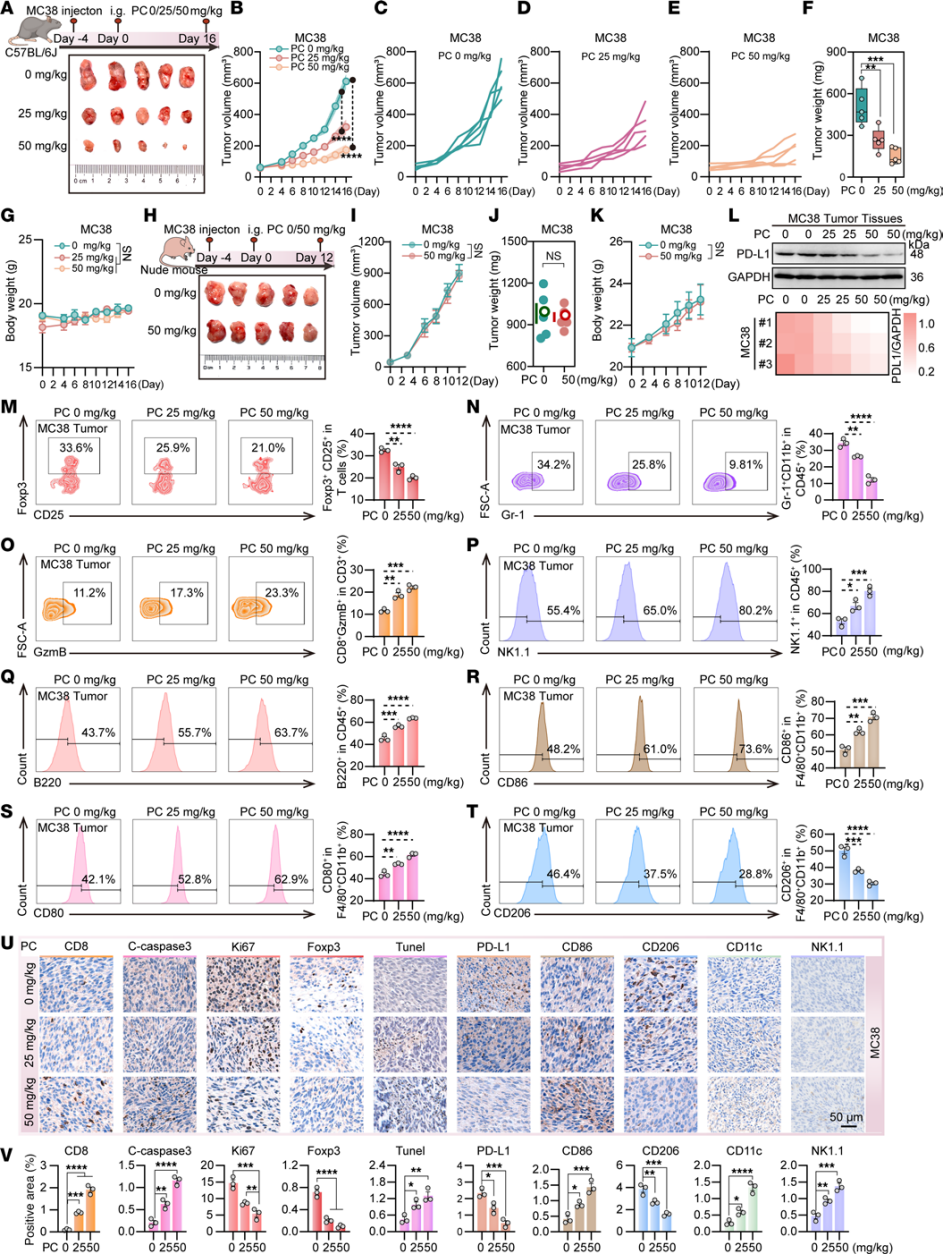
PC attenuates MC38 tumor growth in C57BL/6 mice. C57BL/6 mice or nude mice were subcutaneously implanted with 1 × 10^7^ MC38 colon cancer cells. Upon reaching a tumor volume of approximately 50 mm^3^, different doses of PC were administered via oral gavage. *n* = 5 mice per group. Tissues were harvested approximately 2 weeks after the initiation of treatment. (**A**) Experimental flowchart and tumor schematic in C57BL/6 mice. (**B**–**E**) Tumor growth curves in C57BL/6 mice treated with different doses of PC, specifically showing 0 (**C**), 25 (**D**), and 50 (**E**) mg/kg. (**F**) Statistical analysis of subcutaneous tumor weights in C57BL/6 mice. (**G**) C57BL/6 mice body weight changes. (**H**–**K**) In nude mice bearing MC38 subcutaneous tumors (**H**), 50 mg/kg PC showed no effect on tumor growth (**I**), tumor weight (**J**), or body weight (**K**) versus the 0 mg/kg control. Further analyses were conducted on C57BL/6 mouse tumors: (**L**) Western blot detection of PD-L1 protein expression; (**M**–**T**) flow cytometry analysis of tumor-infiltrating lymphocytes identified the following populations: Tregs (**M**, CD4^+^CD25^+^Foxp3^+^), MDSCs (**N**, CD11b^+^Gr-1^+^ in CD45^+^), cytotoxic T cells (**O**, CD8^+^GzmB^+^), NK cells (**P**, NK1.1^+^), B cells (**Q**, B220^+^), M1 macrophages (**R** and **S**; CD86^+^ and CD80^+^), and M2 macrophages (**T**, CD206^+^). (**U** and **V**) Immunohistochemical analysis of MC38 tumors from PC-treated C57BL/6 mice quantified multiple markers — including CD8, cleaved caspase-3, Ki-67, Foxp3, TUNEL, PD-L1, CD86, CD206, CD11c, and NK1.1 — across 3 representative tumor regions. (**V**) Positive staining was quantified using ImageJ (NIH) and statistically analyzed with GraphPad Prism. The data shown are the mean ± SEM. Statistical differences were determined by 1-way ANOVA with Dunnett’s multiple-comparison test for all panels except **B** and **G** (analyzed by 2-way ANOVA) and **I**–**K** (analyzed by unpaired 2-tailed Student’s *t* test). *P < 0.05, **P < 0.01, ***P < 0.001, and ****P < 0.0001.

To evaluate the antitumor efficacy of PC in vivo, subcutaneous tumor models were established in immunocompetent C57BL/6 mice and nude mice via syngeneic MC38 colon adenocarcinoma cells and Lewis lung carcinoma cells, respectively. In the immunocompetent C57BL/6 mouse model bearing MC38 subcutaneous tumors, the antitumor effects of PC were initially evaluated in a dose-dependent manner at 25 and 50 mg/kg. The results showed that PC substantially inhibited tumor growth, with inhibition rates of 38.1% and 61.1% at 25 and 50 mg/kg, respectively (Figure 2, A–F). During the treatment period, no substantial changes in body weight were observed (Figure 2G), indicating good systemic tolerability of PC. Based on these dose efficacy results, a dose of 50 mg/kg was selected for administration to T cell–deficient nude mice. Compared with the control group, PC did not have a substantial antitumor effect on the nude mice (Figure 2, H–J), and the body weight curves completely overlapped with those of the control group (there was no statistically substantial difference between the groups, *P* > 0.05; Figure 2K). In addition, similar results were obtained in the lung cancer model (Supplemental Figure 2, A–K). These findings suggest that the antitumor effect of PC is mediated through the activation of the immune system. To systematically validate the immune-dependent mechanism hypothesis, we performed multimodal molecular profiling on subcutaneous tumor tissues from PC-treated C57BL/6 mice. Western blotting analysis revealed dose-dependent downregulation of total PD-L1 protein expression in tumor tissues following 16 days of treatment with 25 mg/kg and 50 mg/kg PC (Figure 2L and Supplemental Figure 2L). Flow cytometry revealed reduced expression of immunosuppressive markers, including CD4^+^CD25^+^Foxp3^+^ regulatory T cells (Tregs) (17, 18) (Figure 2M and Supplemental Figure 2M) and CD11b^+^Gr-1^+^ myeloid-derived suppressor cells (MDSCs) (18, 19) (Figure 2N and Supplemental Figure 2N), alongside elevated granzyme B (GzmB) expression (Figure 2O and Supplemental Figure 2O), indicative of cytotoxic T cell activation (20). Additionally, PC treatment substantially increased the populations of NK1.1^+^ NK cells (21, 22) (Figure 2P and Supplemental Figure 2P) and B220^+^ B cells (23–25) (Figure 2Q and Supplemental Figure 2Q). Tumor-associated macrophages (26–28) polarized into pro-inflammatory M1 or immunosuppressive M2 phenotypes presented dose-dependent alterations: The expression of CD86^+^ and CD80^+^ M1 markers (25, 29) was positively correlated with the PC concentration (Figure 2, R and S, and Supplemental Figure 2, R and S), whereas the expression of CD206^+^ M2 markers (30) showed an inverse relationship (Figure 2T and Supplemental Figure 2T), suggesting PC-driven M1 polarization.

The immunohistochemistry results shown in Figure 2, U and V, and Supplemental Figure 2, U and V, verified the dose-dependent upregulation of CD8^+^ cytotoxic T cell infiltrates and cleaved caspase-3 (apoptosis marker) (31–33), which was concurrent with reduced Ki-67 (proliferation marker) (34) and Foxp3^+^ Tregs. TUNEL assays revealed that PC induced apoptosis in a dose-responsive manner. Furthermore, the level of PD-L1 decreased with PC treatment, whereas the number of M1 macrophages (CD86^+^), CD11c^+^ antigen-presenting cells (35), and NK1.1^+^ NK cells increased. Furthermore, hematoxylin and eosin (H&E) staining of major organs revealed no histopathological abnormalities in PC-treated mice (Supplemental Figure 3A), confirming systemic safety. Collectively, these results demonstrate that PC enhances antitumor immunity by suppressing PD-L1 expression and promoting lymphocyte infiltration while maintaining a favorable safety profile.

### PC prevents AOM/DSS-induced colon cancer.

**Figure 3:**
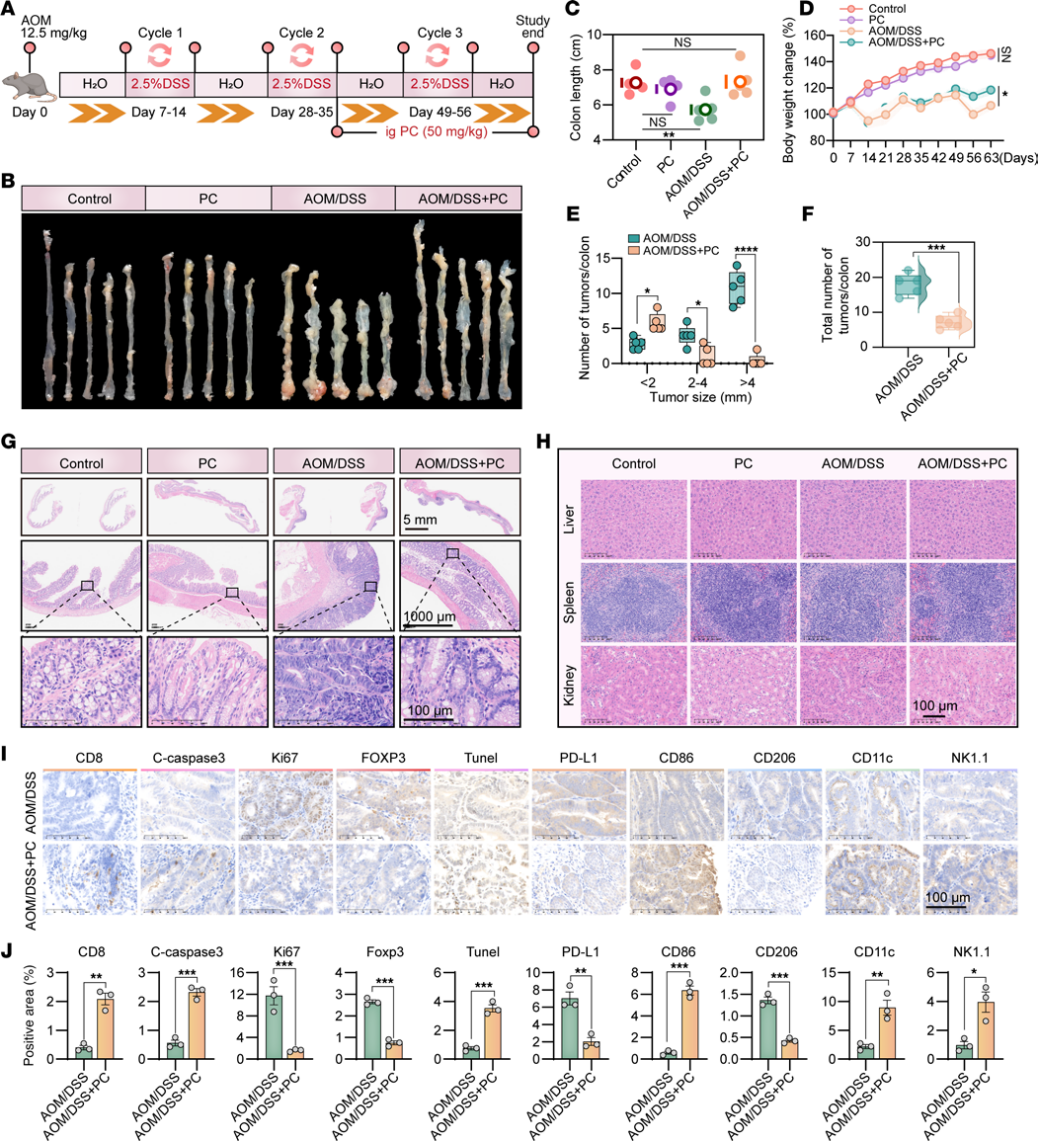
PC demonstrates a significant preventative effect against AOM/DSS-induced colon cancer. (**A**) Flowchart of the AOM/DSS model in male C57BL/6 mice. *n* = 5 mice per group. ig, itragastric. (**B**) Representation of colonic morphology in control, PC (50 mg/kg), AOM/DSS, and AOM/DSS+PC (50 mg/kg) groups. (**C**) Statistical analysis of colon length in control, PC, AOM/DSS, and AOM/DSS+PC groups. 1-way ANOVA with Dunnett multiple comparisons test. (**D**) Body weight changes in mice from control, PC, AOM/DSS, and AOM/DSS+PC groups. 2-way ANOVA with Tukey’s multiple comparison test. (**E**) Statistical analysis of tumor size (< 2 mm^3^, 2–4 mm^3^, > 4 mm^3^) in the colons of mice from AOM/DSS and AOM/DSS+PC groups. 2-way ANOVA with Šídák multiple comparisons test. (**F**) Total number of colonic tumors in AOM/DSS and AOM/DSS+PC groups. (**G**) H&E staining of colon tissues from control, PC, AOM/DSS, and AOM/DSS+PC groups was performed to observe histological changes. (**H**) H&E staining of liver, spleen, and kidney tissues from control, PC, AOM/DSS, and AOM/DSS+PC groups. (**I**) Immunohistochemical analysis of tumor-associated markers (CD8, C-caspase-3, Ki-67, Foxp3, TUNEL, PD-L1, F4/80, CD86, CD206, CD11c, and NK1.1) in colon tissues from AOM/DSS and AOM/DSS+PC groups. For each tumor sample, 3 representative regions of interest (ROIs) were analyzed. The ROIs were selected to focus on viable tumor areas while systematically avoiding obvious necrotic regions, large blood vessels, and tissue folds to ensure an accurate assessment of staining within the tumor parenchyma. Positive markers were then quantified using ImageJ software. (**J**) Quantitative analysis of immunohistochemical markers from **I**. The data shown are the mean ± SEM. (**F** and **J**) Unpaired 2-tailed Student’s *t* test. *P < 0.05, **P < 0.01, ***P < 0.001, and ****P < 0.0001.

To evaluate the therapeutic potential of PC, a well-established system mimicking human inflammation-associated colorectal carcinogenesis through sequential mutagenic (azoxymethane, AOM) and inflammatory (dextran sodium sulfate, DSS) challenges was developed (36) (Figure 3A). Eight-week-old male C57BL/6 mice received intraperitoneal AOM (12.5 mg/kg) followed by 3 cycles of 2.5% DSS (7 days/cycle) interspersed with regular water intervals. PC treatment (50 mg/kg, oral gavage) commenced at the conclusion of the second cycle. After the experiment, comparative analysis of representative colons revealed preserved colon length in the AOM/DSS+PC group compared with the blank control group (*P* > 0.05), whereas substantial shortening was detected in the untreated AOM/DSS group (*P* < 0.05), indicating that PC attenuated DSS-induced inflammation (Figure 3, B and C). Additionally, PC administration mitigated body weight loss (Figure 3D) and reduced the tumor burden across size categories (< 2 mm^3^, 2–4 mm^3^, >4 mm^3^) (Figure 3E), culminating in decreased total tumor multiplicity (Figure 3F). Histopathological evaluation demonstrated marked suppression of neoplastic progression in PC-treated colons (Figure 3G), without hepatosplenic or renal toxicity (Figure 3H). Immunohistochemical profiling revealed that PC dose-dependently modulated tumor immunity, increasing CD8^+^ T cell infiltration, cleaved caspase-3 (apoptosis), TUNEL^+^ cells, M1 macrophages (CD86^+^), CD11c^+^ antigen-presenting cells, and NK1.1^+^ NK cells, coupled with downregulated Ki-67, Foxp3^+^ Tregs, PD-L1, and M2 macrophages (CD206^+^) (Figure 3, I and J). These findings collectively demonstrate that PC counteracts AOM/DSS-driven colon carcinogenesis through immune system potentiation.

### PC and anti–CTLA-4 exert synergistic inhibition of tumor growth.

**Figure 4:**
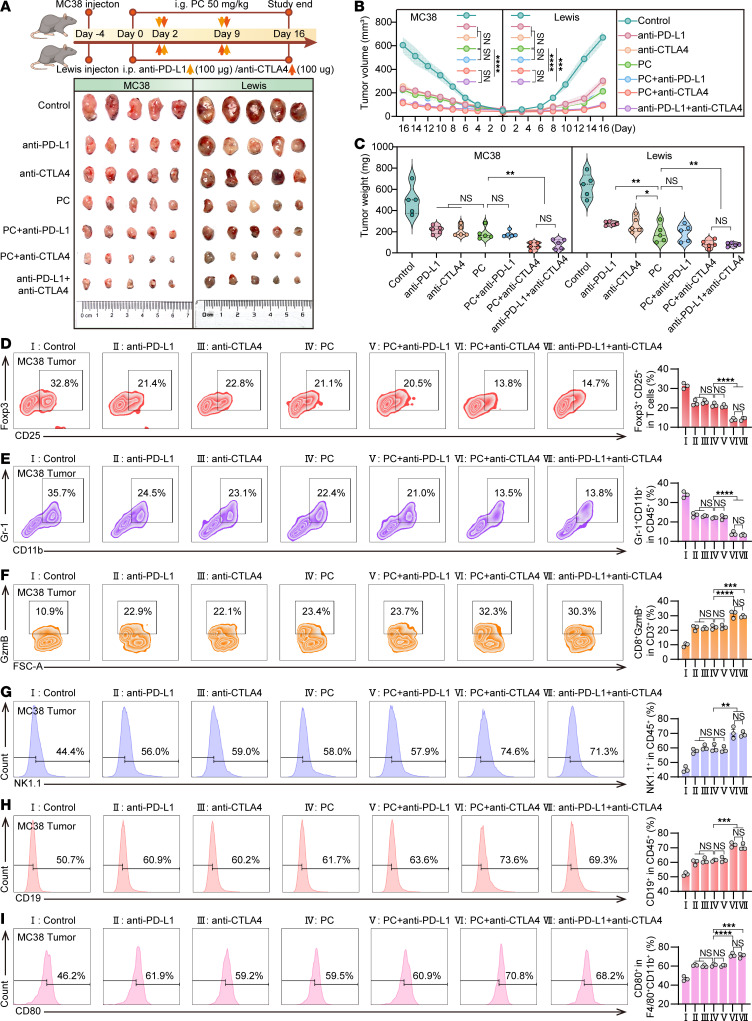
PC combined with anti–CTLA-4 therapy demonstrates enhanced efficacy in the treatment of colon and lung cancers. C57BL/6 mice received subcutaneous transplants of either 1 × 10^7^ MC38 colon cancer cells or 3 × 10^7^ Lewis lung cells. Upon reaching a tumor volume of 50 mm^3^, the mice were randomized into 7 treatment groups. The groups received the following treatments: PBS (vehicle control), anti–PD-L1 (100 μg), anti–CTLA-4 (100 μg), PC (50 mg/kg), PC + anti–PD-L1 antibody, PC + anti–CTLA-4 antibody, or anti–PD-L1 antibody + anti–CTLA-4 antibody. *n* = 5 mice per group. Antibodies were administered via intraperitoneal injection at 1-week intervals. PC was administered daily by oral gavage until sample collection. (**A**) A flowchart illustrating the MC38 or Lewis transplantation tumor model, along with representative images of solid tumors from each treatment group. (**B**) Tumor growth curves for each treatment group in the MC38 or Lewis transplantation tumor model were shown. (**C**) Tumor weight statistics for each group of MC38- and Lewis-transplanted tumor mice. (**D**–**I**) Flow cytometry analysis of the proportions of tumor-associated lymphocyte markers in tumor tissues from MC38-transplanted tumor-bearing mice in each group and their statistical analysis. **D**–**I** represent the expression levels of CD4^+^CD25^+^Foxp3^+^, CD11b^+^Gr-1^+^, CD8^+^GzmB^+^, NK1.1^+^, CD19^+^, and CD80^+^, respectively, in the tumor tissues of each group. The data shown are the mean ± SEM. Statistical differences were determined by 2-way ANOVA test. *P < 0.05, **P < 0.01, ***P < 0.001, and ****P < 0.0001.

Clinical trials have demonstrated improved efficacy of anti–PD-1/PD-L1 antibodies when they are administered in combination with CTLA-4 inhibitors (37–39). To assess the potential of PC as a PD-L1 inhibitor, we established colon cancer (MC38) and lung cancer (Lewis) graft tumor models in C57BL/6 mice. The experimental groups received various treatments, including PBS, anti–PD-L1 (100 μg), anti–CTLA-4 (100 μg), PC (50 mg/kg), PC + anti–PD-L1, PC + anti–CTLA-4, and anti–PD-L1 + anti–CTLA-4. PC monotherapy resulted in comparable tumor growth inhibition to anti–PD-L1 or anti–CTLA-4 monotherapy in both models (Figure 4, A–C, and Supplemental Figure 4, A–N). While PC + anti–PD-L1 showed no additive effects versus single agents, both PC + anti–CTLA-4 and anti–PD-L1 + anti–CTLA-4 combinations demonstrated superior tumor control relative to monotherapies (Supplemental Figure 4O), without inducing substantial weight fluctuations. These findings suggest that PC can be effectively combined with anti–CTLA-4 therapy for enhanced treatment of colon and lung cancer. Tumor tissues from each group were analyzed by flow cytometry. The results revealed that mice subjected to PC + anti–CTLA-4 and anti–PD-L1 + anti–CTLA-4 combination therapies exhibited markedly lower frequencies of CD4^+^CD25^+^Foxp3^+^ Tregs (Figure 4D and Supplemental Figure 5A) and MDSCs (CD11b^+^Gr-1^+^) (Figure 4E and Supplemental Figure 5B) within the tumor microenvironment compared with other treatment groups. Conversely, these combination groups presented substantially elevated levels of cytotoxic markers (GzmB^+^) (Figure 4F and Supplemental Figure 5C), NK cells (NK1.1^+^) (Figure 4G and Supplemental Figure 5D), B cells (CD19^+^, Figure 4H; and B220^+^, Supplemental Figure 5E), and M1-polarized macrophages (CD86^+^, Supplemental Figure 5F; CD80^+^, Figure 4I and Supplemental Figure 5G; and CD11c^+^, Supplemental Figure 6A). In contrast, the expression of the M2 macrophage marker CD206^+^ was minimal in these groups (Supplemental Figure 5H and Supplemental Figure 6B). Immunohistochemical analysis further highlighted the profound antitumor effects of the combination therapies. Tumor tissues from the PC + anti–CTLA-4 and anti–PD-L1 + anti–CTLA-4 groups displayed substantial increases in CD8^+^ T cells, cleaved caspase-3, and TUNEL-positive cells, indicating enhanced cytotoxicity and apoptosis. Concurrently, there were pronounced reductions in Ki-67^+^ proliferating cells and Foxp3^+^ Tregs (Supplemental Figure 6, C and D, and Supplemental Figure 7, A and B). Overall, these findings demonstrate that PC can be effectively substituted for anti–PD-L1 in combination with anti–CTLA-4 to elicit synergistic antitumor responses.

### PC downregulates the expression of PD-L1 via the ubiquitin/proteasome pathway.

**Figure 5:**
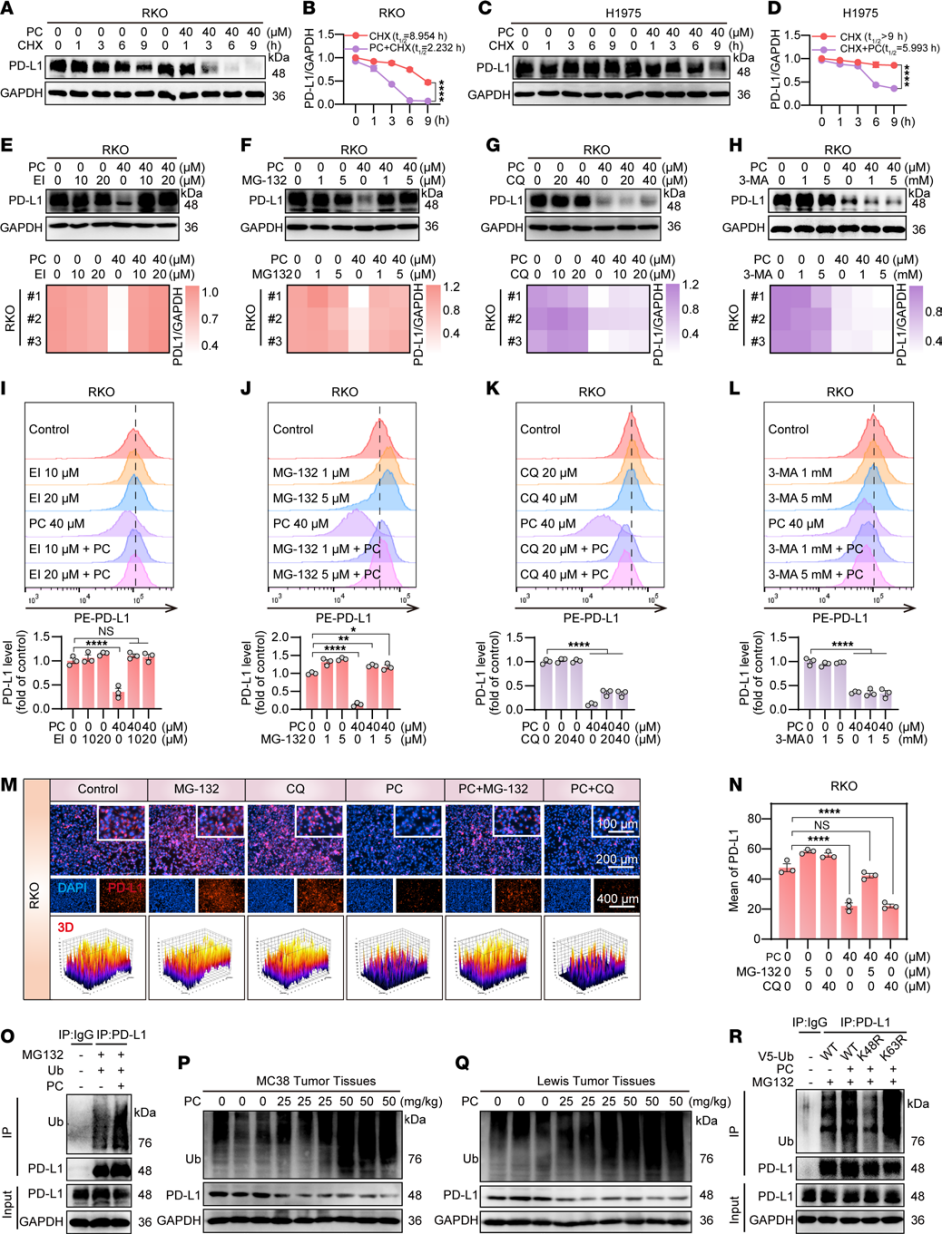
PC promotes K48-linked polyubiquitination of PD-L1 for its degradation. (**A**–**D**) RKO (**A**) and H1975 (**C**) cells were cotreated with 60 μg/mL CHX and 40 μM PC. The PD-L1 protein expression was detected by Western blot at 0, 1, 3, 6, and 9 hours posttreatment. Quantification is shown in **B** and **D**. (**E**–**L**) RKO cells were treated with different concentrations of the following compounds and 40 μM PC: eeyarestatin I (EI; 10, 20 μM), MG132 (1, 5 μM), chloroquine (CQ; 20, 40 μM), or 3-methyladenine (3-MA; 1, 5 mM). The expression of PD-L1 was subsequently examined via Western blotting (**E**–**H**) and flow cytometry (**I**–**L**), followed by statistical analysis. (**M** and **N**) PD-L1 expression was detected by immunofluorescence in RKO cells cotreated with 40 μM PC and either 5 μM MG-132 or 40 μM CQ. Blue fluorescence indicates cell nuclei, whereas red fluorescence indicates PD-L1 levels on the cell membrane. Scale bars of 100 μm, 200 μm, and 400 μm were used. (**N**) Quantitative analysis. (**O**) PD-L1 was immunoprecipitated from PC-treated RKO cell lysates, and the level of ubiquitination was assessed via Western blotting with a ubiquitin (Ub) antibody. (**P** and **Q**) C57BL/6 mice subcutaneously transplanted with MC38 (**P**) or Lewis (**Q**) cells were treated with different doses of PC. Western blotting with an anti-Ub antibody was used to detect PD-L1 ubiquitination in tumor tissues. (**R**) RKO cells expressing WT ubiquitin (WT-Ub), K48R-Ub, or K63R-Ub were treated with PC. After immunoprecipitation with an anti–PD-L1 antibody, PD-L1 ubiquitination was analyzed via Western blotting with an anti-Ub antibody. The data shown are the mean ± SEM of triplicate experiments. Statistical differences were determined by 1-way ANOVA with Dunnett’s multiple-comparison test. *P < 0.05, **P < 0.01, and ****P < 0.0001.

To investigate the mechanisms driving PC-induced PD-L1 downregulation, we first evaluated PD-L1 transcriptional dynamics in RKO cells via RT-qPCR following either dose-dependent (0 μM, 10 μM, 20 μM, or 40 μM) or time-dependent (0 hours, 3 hours, 6 hours, 9 hours, 12 hours, or 24 hours) PC treatment. Notably, these analyses revealed only modest transcriptional suppression (≤50%) of PD-L1 (Supplemental Figure 8A), suggesting that posttranscriptional regulation predominates. To assess protein-level regulation directly, we subjected RKO and H1975 cells to combined treatment with PC and the translational inhibitor cycloheximide (CHX). PC substantially accelerated the degradation of PD-L1 protein, reducing the protein half-life to approximately 2.2 hours in RKO cells and 6.0 hours in H1975 cells (Figure 5, A–D). Collectively, these findings suggest that PC degrades PD-L1 primarily at the protein level.

Intracellular protein homeostasis is maintained through the degradation of excess or misfolded proteins following synthesis, which is regulated primarily by the ubiquitin/proteasome pathway and the autophagy/lysosome pathway (40). To identify the specific pathway and location of PD-L1 degradation by PC, we treated RKO cells with PC in combination with various inhibitors: eeyarestatin I (EI; an ER-associated protein synthesis inhibitor), MG132 (a proteasome inhibitor), chloroquine (CQ; a lysosomal inhibitor), and 3-methyladenine (3-MA; an autophagy inhibitor). Western blotting analysis demonstrated that only EI and MG132 effectively rescued PD-L1 protein levels from PC-mediated degradation (Figure 5, E–H, and Supplemental Figure 8, B and C). This rescue effect was corroborated by flow cytometric (Figure 5, I–L) and IF assay (Figure 5, M and N) quantification of membrane PD-L1 expression, where EI and MG132 treatments restored the PD-L1 surface abundance to levels that were not substantially different from those in the control group. In contrast, lysosomal (CQ) and autophagic (3-MA) inhibitors had no substantial protective effects. These results indicate that PC primarily degrade PD-L1 on the ER through the ubiquitin/proteasome pathway.

To verify whether PC promotes PD-L1 ubiquitination, immunoprecipitation assays were performed using RKO cells. Western blotting analysis revealed a marked increase in ubiquitin chains associated with PD-L1 following PC treatment (Figure 5O). Additionally, we examined the ubiquitination levels in tumor tissues from C57BL/6 mice with subcutaneous transplantation of MC38 and Lewis cells and treatment with different doses of PC. As shown in Figure 5, P and Q, the ubiquitination levels in tumor tissues increased with increasing doses of PC, accompanied by correspondingly decreased PD-L1 levels. K48 and K63 are the 2 most common types of polyubiquitin chain linkages. K48-linked polyubiquitination is the primary pathway for PD-L1 degradation (41, 42). In contrast, K63 ubiquitination does not directly lead to PD-L1 degradation but may be involved in PD-L1 endocytosis and intracellular transport, thereby affecting its cell surface expression (43–45). To determine the specific ubiquitination sites mediated by PC on PD-L1, site-specific ubiquitin-mutant plasmids (Ub-K48R/K63R) were used. PD-L1 immunoprecipitation captured ubiquitinated complexes, and Western blotting analysis (Figure 5R) revealed that in the Ub-K63R mutant group, a substantial ubiquitination signal was still detected after PC treatment, whereas the Ub-K48R mutation completely blocked PC-induced ubiquitination. In summary, PC achieves targeted degradation of the PD-L1 protein by precisely regulating the assembly of K48-specific polyubiquitin chains.

### PC promotes PD-L1 ubiquitination and degradation by targeting SYVN1.

**Figure 6:**
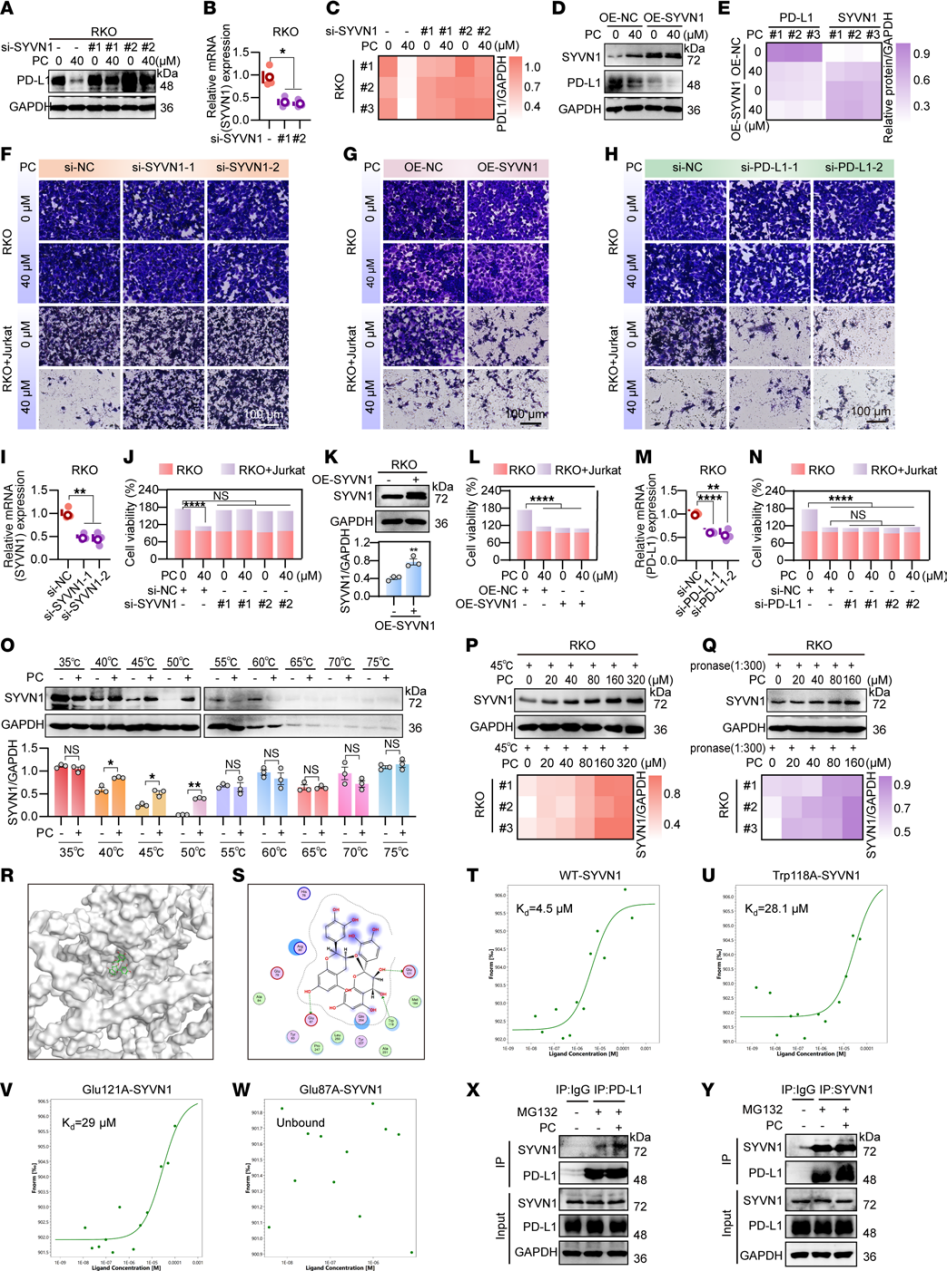
PC downregulates PD-L1 expression via targeting SYVN1. (**A**–**C**) Following SYVN1 knockdown in RKO cells, Western blotting detected PD-L1 protein levels after PC treatment (**A**). si, siRNA. SYVN1 knockdown efficiency was validated by RT-qPCR (**B**). (**C**) Quantification of **A**. (**D**) Western blotting analysis of PD-L1 protein expression in RKO cells overexpressing SYVN1 and cocultured with PC. (**E**) Quantification of **D**. (**F**–**H**) RKO cells with SYVN1 knockdown (**F**), SYVN1 overexpression (**G**), or PD-L1 knockdown (**H**) were cocultured with PD-1–overexpressing Jurkat cells (activated with PHA/PMA) at a 9:1 E:T ratio for 24 hours, in the presence or absence of PC, to assess tumor cell killing. The remaining RKO cells were stained with crystal violet and imaged (Cytation 5; scale bar: 100 μm). (**I**) SYVN1 knockdown efficiency was measured by RT-qPCR. (**J**) Quantification of **F**. (**K**) SYVN1 overexpression was verified by Western blotting and quantified. (**L**) Quantification of **G**. (**M**) PD-L1 knockdown efficiency was measured by RT-qPCR. (**N**) Quantification of **H**. (**O**–**Q**) PC binding to SYVN1 was demonstrated by CETSA at graded temperatures (**O**) and a fixed 45°C with different PC doses (**P**) and by the drug affinity responsive target stability assay under various PC concentrations (1:300 ratio) (**Q**). (**R** and **S**) Molecular docking of PC with SYVN1. (**T**–**W**) MST assay of GFP-tagged SYVN1 cells overexpressing WT SYVN1 (**M**) or SYVN1 mutants (Trp118A, Glu121A, and Glu87A) (**N**–**P**). (**X** and **Y**) Immunoprecipitation experiments confirmed that PC enhances the interaction between the PD-L1 and SYVN1 proteins. Cell lysates were co-precipitated using antibodies against PD-L1 (**X**) or SYVN1 (**Y**). The data shown are the mean ± SEM of triplicate experiments. (**B**, **I**, **K**, **M**) Significance was determined using Student’s *t* test; (**O**) 2-way ANOVA with Šídák multiple comparisons test; All other panels: 1-way ANOVA with Dunnett multiple comparisons test. *P < 0.05, **P < 0.01, and ****P < 0.0001.

Previous studies have shown that PD-L1 ubiquitination is dynamically regulated by E3 ubiquitin ligases such as SPOP (46), STUB1 (47), and SYVN1 (48, 49) and is counteracted by deubiquitinating enzymes (DUBs) such as USP22 (50, 51) and OTUB1 (52). To identify the key E3 ligases or DUBs that mediate PC-induced PD-L1 degradation, we systematically silenced candidate E3 ligases — ARIH1 (53), β-TrCP, MARCH8 (54), STUB1, SPOP, and SYVN1 — via siRNA or overexpressed DUBs — USP22 and OTUB1 — in RKO cells and then assessed the impact of PC on PD-L1 levels (Supplemental Figure 9, A and F). As shown in Figure 6, A–C, only SYVN1 knockdown counteracted the degradation of PD-L1 by PC in RKO cells. Additionally, SYVN1 overexpression did not substantially increase PC degradation of PD-L1 (Figure 6, D and E). Consistently, Figure 6, F–N, indicate that the PC-mediated increase in the cytotoxicity of Jurkat cells to RKO cells was reversed by SYVN1 knockdown. Moreover, neither overexpressing SYVN1 nor knocking down PD-L1 further enhanced this cytotoxic effect. In summary, these results indicate that PC enhances in vitro immune responses by downregulating PD-L1 through targeting SYVN1. These findings suggest that PC may target SYVN1 to increase its stability, thereby degrading PD-L1.

To verify this hypothesis, we performed a cellular thermal shift assay (CETSA), which is primarily used to confirm the binding of drugs to target proteins (55, 56). We incubated RKO cell lysates with 0 μM or 200 μM PC and found that PC substantially reversed the degradation of the SYVN1 protein at 40°C, 45°C, and 50°C (Figure 6O). Next, we incubated the lysates with different concentrations of PC at 45°C for the same duration and observed that the stability of the SYVN1 protein increased with increasing PC concentration (Figure 6P). This effect was also evident in the presence of proteases (Figure 6Q). Molecular docking analysis via the Molecular Operating Environment (MOE) platform identified Trp118, Glu121, and Glu87 as potential binding residues at the PC-SYVN1 interface (Figure 6, R and S). To validate these predictions, GFP-tagged WT SYVN1 and point mutants (W118A, E121A, and E87A) were expressed in HEK293T cells. Microscale thermophoresis (MST) binding assays revealed a PC-WT SYVN1 dissociation constant (*K_D_*) of 4.5 μM (Figure 6T). While the W118A and E121A mutations exhibited comparable binding affinities to WT (Figure 6, U and V), the E87A mutation completely abolished the PC-SYVN1 interaction (Figure 6W). These structural and functional data establish that Glu87 is the critical binding site for PC on SYVN1.

**Figure 7:**
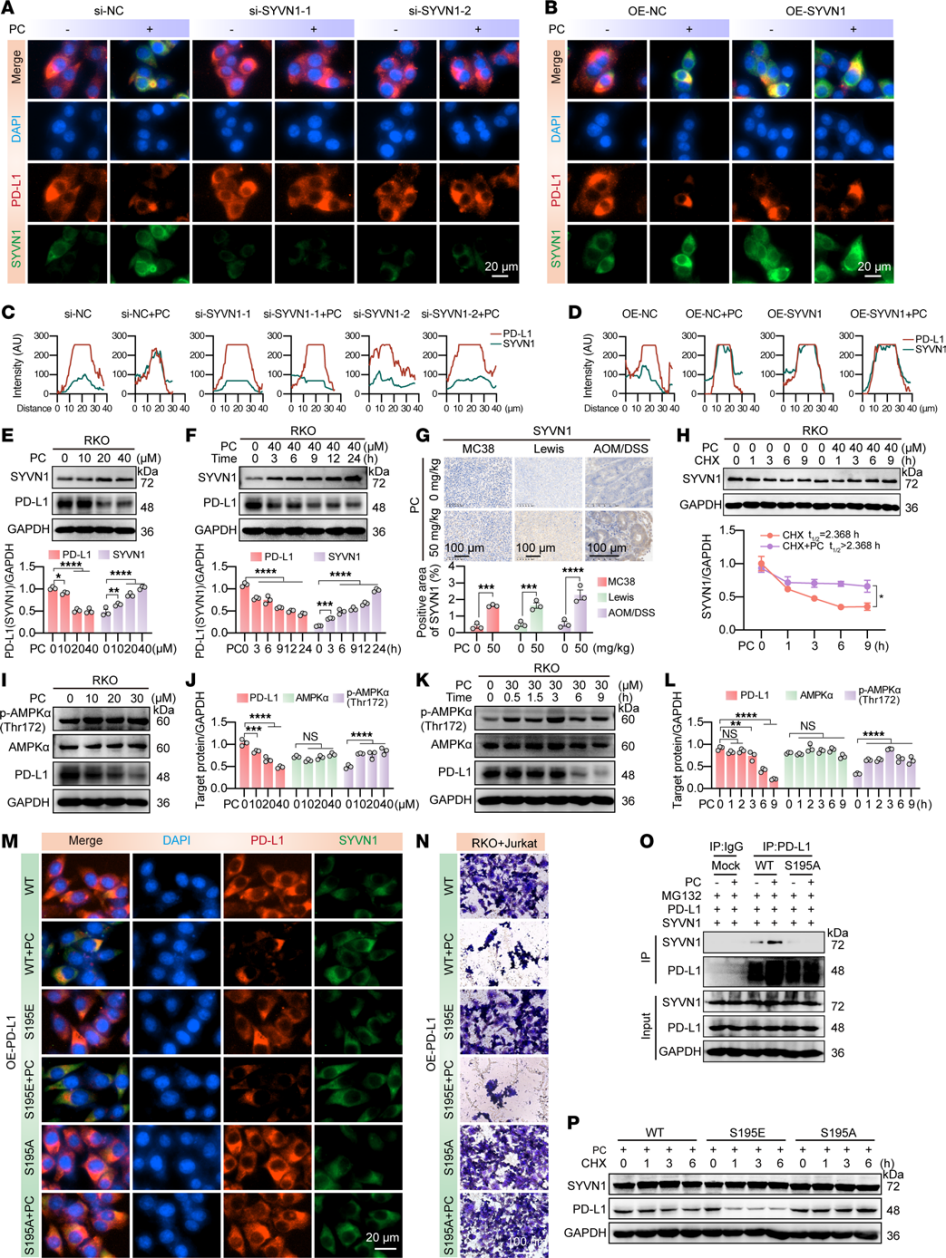
PC targets SYVN1 while promoting AMPK phosphorylation and thus induces PD-L1 ubiquitination-mediated degradation. (**A**–**D**) Immunofluorescence images showing colocalization of PD-L1 (red) and SYVN1 (green) in RKO cells with SYVN1 knockdown (**A**) or overexpression (**B**), cocultured with 40 μM PC. Nuclei are stained blue. Scale bar: 20 μm. (**C** and **D**) Quantitative analysis of **A** and **B**, respectively. (**E** and **F**) Western blot analysis of SYVN1 and PD-L1 protein levels in RKO cells treated with PC at different concentrations (**E**) or for different durations (**F**), with quantification and statistical analysis. (**G**) Immunohistochemical staining and quantitative analysis of SYVN1 expression in tumor tissues from C57BL/6 mice subcutaneously transplanted with MC38 or Lewis cells and in intestinal tissues from PC-treated AOM/DSS model mice. (**H**) Western blot determination of SYVN1 protein half-life in RKO cells treated with CHX and PC. (**I**–**L**) Western blot analysis of phosphorylated and total AMPKα in PC-treated RKO cells under varying concentrations (**I**) or time points (**K**); **J** and **L** show quantitation of **I** and **K**, respectively. (**M**) Immunofluorescence colocalization of SYVN1 and PD-L1 in cells overexpressing WT, S195A, or S195E PD-L1, with or without PC. Scale bar: 20 μm. (**N**) Crystal violet staining and imaging (Cytation 5) of residual tumor cells after coculture of Jurkat cells with RKO cells overexpressing WT, S195A, or S195E PD-L1, followed by PC treatment. Scale bar: 100 μm. (**O**) Co-IP for SYVN1–PD-L1 interaction after PC treatment with indicated PD-L1 variants. (**P**) PD-L1 Western blot in CHX and PC cotreated RKO cells expressing the specified PD-L1 variants. The data shown are the mean ± SEM of triplicate experiments. Statistical differences were determined by 2-way ANOVA with Dunnett’s multiple-comparison test. *P < 0.05, **P < 0.01, ***P < 0.001, and ****P < 0.0001.

To further investigate whether PC promotes the interaction between SYVN1 and PD-L1, we conducted immunoprecipitation experiments in RKO cells using antibodies against PD-L1 and SYVN1. The results as shown in Figure 6, X and Y, indicated that PC enhanced the interaction between SYVN1 and PD-L1. This finding was further supported by IF colocalization experiments, which also demonstrated that PC strengthened the interaction between SYVN1 and PD-L1. However, SYVN1 overexpression did not further enhance this interaction (Figure 7, A–D), whereas SYVN1 knockdown reversed it. Additionally, cell experiments and immunohistochemistry revealed that SYVN1 expression increased with increasing PC concentration (Figure 7, E–G). Further RNA experiments indicated that this increase was not due to transcription (Supplemental Figure 10A), but rather that PC increased SYVN1 stability by extending its half-life (Figure 7H). These findings suggest that PC enhances SYVN1 protein stability in RKO cells.

### PC promotes the proteasomal degradation of PD-L1 by targeting LKB1-mediated AMPK phosphorylation.

Our findings indicate that PC promotes PD-L1 degradation within the ER. Studies have shown that activated AMPK induces the phosphorylation of PD-L1 at Ser195 (57). This phosphorylation triggers abnormal glycosylation of PD-L1, causing its accumulation in the ER and subsequent degradation via the E3 ligase SYVN1. Our experiments also demonstrated that PC activated AMPK in a dose-dependent manner. Together with PC-mediated targeting of SYVN1, these findings suggest dual mechanisms by which PC promotes abnormal PD-L1 glycosylation via AMPK activation and enhances PD-L1 degradation by targeting SYVN1 (Figure 7, I–L). Next, we constructed PD-L1 plasmids with WT, S195A (nonphosphomimetic), or S195E (phosphomimetic) mutations (57). IF revealed that the S195A mutation reversed PC-mediated PD-L1 degradation, whereas degradation persisted in the WT and S195E contexts (Figure 7M and Supplemental Figure 10B). In vitro cytotoxicity assays showed that only the S195A mutation abrogated PC-enhanced T cell–mediated killing of RKO cells (Figure 7N and Supplemental Figure 10C). Co-immunoprecipitation assays demonstrated that the S195A mutation abolished the PC-induced interaction between PD-L1 and SYVN1 (Figure 7O). Moreover, Western blotting analysis revealed that, upon cotreatment with CHX and PC, the S195E mutation accelerated PD-L1 degradation, whereas S195A counteracted this effect (Figure 7P and Supplemental Figure 10D).

**Figure 8:**
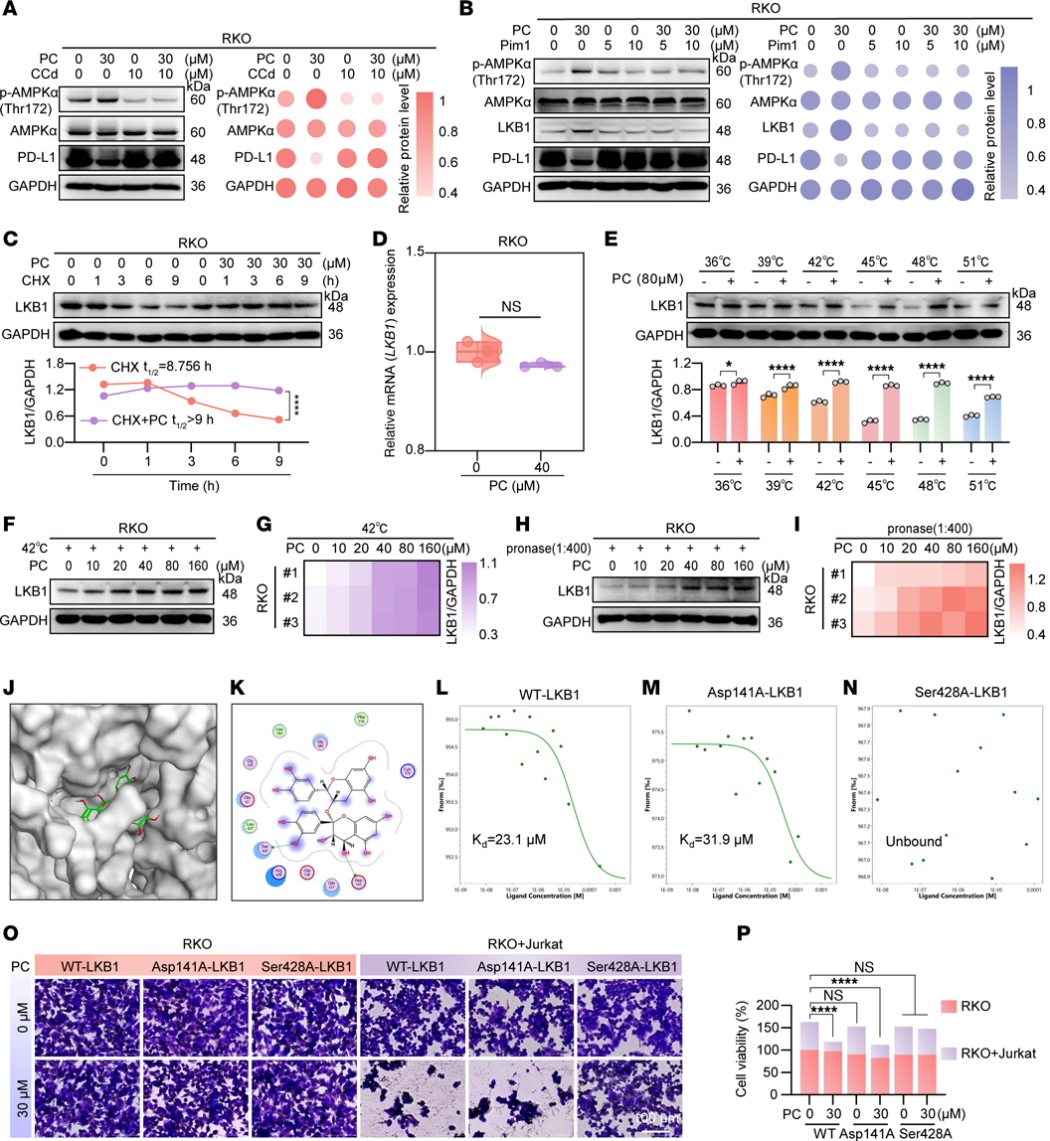
PC targets LKB1 to activate AMPK phosphorylation and downregulate PD-L1. (**A**) RKO cells were pretreated with 10 μM compound C dihydrochloride (CCd) (10 μM) or vehicle (2 hours), then 30 μM PC (9 hours). Immunoblotting for p-AMPKα (Thr172), AMPKα, PD-L1. (**B**) Western blot analysis of RKO cells treated with Pim1 (5, 10 μM; 2 hours) ± 30 μM PC (9 hours) for p-AMPKα (Thr172), AMPKα, LKB1, and PD-L1. (**C**) RKO cells were cotreated with CHX and PC or vehicle, and the LKB1 half-life was assessed by Western blotting. (**D**) LKB1 mRNA levels in RKO cells after 40 μM PC treatment for 9 hours (RT-qPCR). (**E**) CETSA was used to evaluate PC-induced thermal stabilization of LKB1 across a range of temperatures. (**F** and **G**) LKB1 thermal stability at 42°C under increasing PC concentrations and quantification of these results. (**H** and **I**) LKB1 proteolytic susceptibility was assessed by DARTS assay at PC-to-protein ratios of 1:400 (**H**), with quantification shown in **I**. (**J** and **K**) Molecular docking simulations illustrating potential binding modes between PC and LKB1. (**L**–**N**) MST analysis of the interaction between PC and GFP-tagged WT LKB1 (**L**) or its mutants (Asp141A, Ser428A) (**M** and **N**). (**O**) RKO cells expressing GFP-tagged WT or mutant LKB1 (Asp141A, Ser428A) were treated with or without 30 μM PC for 9 hours, followed by a 24-hour coculture with PHA/PMA-activated Jurkat T cells at a 9:1 ratio. Surviving cells were quantified by crystal violet staining and Cytation 5 imaging. (**P**) The quantitative plot of **O**. The data shown are the mean ± SEM. Statistical differences were determined by unpaired 2-tailed Student’s *t* test (**D**), 2-way ANOVA with Šídák multiple comparisons test (**E**), and 1-way ANOVA with Dunnett multiple comparisons test (all other panels). *P < 0.05, and ****P < 0.0001.

To further elucidate how PC triggers AMPK phosphorylation, we first verified whether PC-mediated PD-L1 downregulation depends on AMPK activation. Using the AMPK inhibitor compound C dihydrochloride, we verified that AMPK inhibition abrogates PC-induced PD-L1 reduction, establishing AMPK as a critical mediator (Figure 8A). We then investigated the upstream mechanism of AMPK activation. Drawing on published evidence implicating LKB1 as a key upstream kinase of AMPK (58, 59), we hypothesized that LKB1 may be involved. Consistent with these findings, the LKB1 inhibitor Pim1 abolished the effect of PC on PD-L1 downregulation (Figure 8B), suggesting that LKB1 acts upstream of AMPK in the PC-induced signaling pathway.

Subsequent mechanistic studies, as shown in Figure 8, C and D, revealed that PC prolongs the half-life of LKB1 protein without altering its mRNA levels, ruling out transcriptional regulation. CETSA (Figure 8, E–G) and streptavidin pulldown experiments (Figure 8, H and I) further demonstrated that PC enhances LKB1 thermal stability. Molecular docking analysis predicted potential binding sites between PC and LKB1 in Figure 8, J and K, which was further validated by MST indicating that Ser428 is the critical binding residue (Figure 8, L–N). Mutation of Ser428 not only disrupted the PC–LKB1 interaction but also abrogated the ability of PC to enhance the tumor-killing capacity of Jurkat cells (Figure 8, O and P). Collectively, these results define a coherent posttranslational regulatory axis wherein PC binds to and stabilizes LKB1, leading to AMPK activation, abnormal glycosylation of PD-L1, SYVN1 recruitment, and ultimately PD-L1 ubiquitination and degradation.

### LKB1 and SYVN1 in cancer immunotherapy and patient survival.

**Figure 9:**
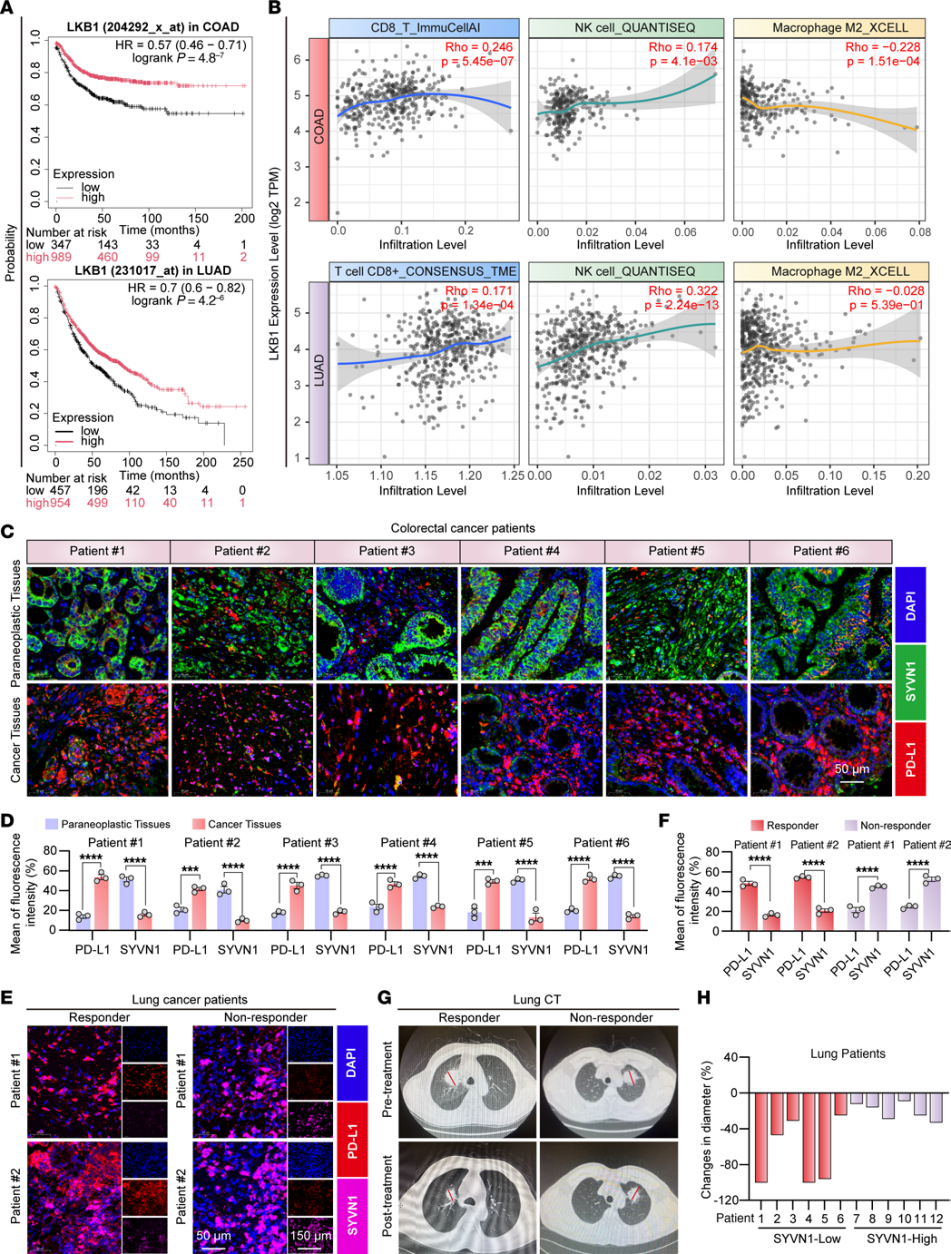
LKB1 and SYVN1 are implicated in cancer therapy and immune regulation. (**A**) Kaplan-Meier plotter analysis indicates that elevated LKB1 expression is associated with improved survival in patients with COAD and LUAD. (**B**) Analysis using the TIMER algorithm shows that LKB1 levels are positively associated with immune-related markers — specifically CD8^+^ T cells and NK cells — while exhibiting a negative correlation with M2 macrophages in patients with COAD and LUAD. (**C**) Fluorescence double-staining immunohistochemistry was used to detect PD-L1 and SYVN1 expression levels in paraneoplastic and colorectal cancer tissues from patients with colorectal cancer. Scale bar: 50 μm. (**D**) Quantitative statistical plot of **C**. (**E**) Representative images showing SYVN1 and PD-L1 expression in the lung tissues of patients with lung cancer. Scale bar: 50 μm. (**F**) Statistical quantification of the data in **E**. (**G**) Tumor image maps of 2 patients with lung cancer treated with an anti–PD-1 mAb showing the tumor size before and after treatment. (**H**) Patients with lung cancer who responded and did not respond to immunotherapy had different changes in tumor diameter. The data shown are the mean ± SEM of triplicate experiments. Statistical differences were determined by 2-way ANOVA with Šídák’s multiple-comparison test. ***P < 0.001, and ****P < 0.0001.

Analysis of the Kaplan-Meier survival curve (log-rank test, *P* < 0.05) indicated that higher LKB1 expression in colorectal cancer (COAD) and lung adenocarcinoma (LUAD) was associated with improved overall survival (Figure 9A). Furthermore, analysis of the TIMER database demonstrated that elevated LKB1 levels correlated with increased infiltration of CD8^+^ T cells and NK cells and decreased infiltration of M2 macrophages in these cancers (Figure 9B). These results suggest that high tumor LKB1 expression may activate antitumor immunity.

Previous studies have indicated that SYVN1 may inhibit PD-L1 expression in tumor tissues (60). To further investigate the relationship between SYVN1 expression and cancer treatment, we assessed the infiltration levels of tumor-infiltrating immune cells in patients with rectal cancer (READ), COAD, and LUAD via 2 independent algorithms. Consistent with previous findings, SYVN1 expression was positively correlated with the infiltration levels of B cells, CD8^+^ T cells, NK cells, macrophages, and dendritic cells across all datasets (Supplemental Figure 11, A–C).

We also analyzed PD-L1 and SYVN1 expression levels in paracancerous and cancerous tissues from patients with COAD. As depicted in Figure 9, C and D, cancer tissues presented substantially higher PD-L1 expression compared with paracancerous tissues, while SYVN1 expression showed the opposite trend. These results suggest that elevated SYVN1 expression in tumors may suppress PD-L1, thereby enhancing anticancer efficacy.

Furthermore, we evaluated SYVN1 and PD-L1 protein expression levels via IF staining in tumor biopsy tissues from patients with NSCLC treated with PD-1 monoclonal antibody (mAb) (Figure 9, E and F, and Supplemental Tables 1 and 2. The sources for all cell lines and reagents used are compiled in Supplemental Table 3, and the relevant antibodies are shown in Supplemental Table 4.) Patients were categorized as responders or nonresponders based on their response to PD-1 mAb therapy. Compared with nonresponders, responders had higher PD-L1 expression and lower SYVN1 expression in their biopsy tissues. These findings are in line with previous studies demonstrating that SYVN1 expression is negatively correlated with PD-L1 expression and that patients with elevated PD-L1 levels tend to respond better to immunotherapy.

Additionally, radiographic images from 2 representative lung cancer cases supported these findings (Figure 9G and Supplemental Tables 1 and 2). Analysis of tumor diameter changes in all patients with lung cancer (Figure 9H) revealed a negative correlation between tumor diameter reduction and SYVN1 expression. In conclusion, patients with high PD-L1 expression and low SYVN1 expression are more likely to benefit from PD-L1 checkpoint inhibitors.

## Discussion

In this study, we identified PC as a natural immune checkpoint modulator that destabilizes PD-L1 via an endoplasmic reticulum–associated degradation–dependent (ERAD-dependent) mechanism. First, PC binds to LKB1 and stabilizes it, which activates AMPK. Activated AMPK then induces PD-L1 phosphorylation at Ser195, and this phosphorylation impairs the normal glycosylation of PD-L1 while promoting its ER retention. Second, PC interacts with the E3 ubiquitin ligase SYVN1 to improve SYVN1’s protein stability. This increased stability strengthens PD-L1–SYVN1 binding and promotes K48-linked ubiquitination as well as proteasomal degradation of ER-retained PD-L1. This dual targeting mechanism effectively suppresses tumor growth in colorectal and lung cancer models, synergizes with CTLA-4 blockade, and attenuates colitis-associated colorectal cancer (CRC) in AOM/DSS-induced models. Our findings not only elucidate an ERAD/immune checkpoint axis but also highlight the therapeutic potential of small-molecule compounds such as PC in cancer immunotherapy.

The role of LKB1 as a master upstream kinase in regulating AMPK signaling and cellular metabolism is well established (59, 61). Our data revealed that PC directly binds to LKB1, stabilizing it and prolonging its protein half-life, which in turn drives AMPK-mediated PD-L1 abnormal glycosylation. This LKB1/AMPK/PD-L1 axis represents a critical posttranslational regulatory pathway that links metabolic sensing to immune evasion. Importantly, analysis of clinical databases indicates that high LKB1 expression in colorectal and lung adenocarcinomas is correlated with improved overall survival and enhanced infiltration of cytotoxic CD8^+^ T cells and NK cells, while suppressing M2 macrophage polarization. These findings position LKB1 not only as a critical functional target of PC but also as a potential predictive biomarker for the response to ICIs. In tumors with intact LKB1, PC exerts its effect by stabilizing the LKB1 protein, thereby inducing PD-L1 degradation. For tumors harboring LKB1 loss or mutation, our data suggest a potential therapeutic strategy of combining PC with LKB1 agonists to restore LKB1 function and reverse immune evasion, thereby offering a potentially novel avenue to overcome ICI resistance in LKB1-deficient tumors.

Small-molecule ICIs offer distinct advantages over mAbs, which have dominated clinical immunotherapy (62, 63). While antibodies such as anti–PD-L1/PD-1 agents exhibit high specificity, their large size limits tissue penetration, particularly in poorly vascularized or fibrotic tumors, and they cannot target intracellular pathways (64). In contrast, small molecules such as PC possess superior bioavailability, oral administration potential, and enhanced diffusion capacity (65), enabling them to access intracellular targets such as LKB1 and SYVN1 within the tumor microenvironment. Moreover, small molecules can be engineered for combination regimens with existing therapies without the pharmacokinetic challenges of antibody-based combinations (66–68). Recent advances in drug development have emphasized the potential of small molecules to overcome adaptive resistance mechanisms, such as compensatory upregulation of alternative checkpoints or aberrant glycosylation, which antibodies may not adequately address (69). The dual effects of PC on LKB1 stabilization and SYVN1 enhancement exemplify this next-generation approach, providing a multifaceted strategy to sustain PD-L1 degradation and amplify T cell responses. Importantly, the oral activity and favorable toxicity profile of PC in our models underscore its translational promise as a cost-effective and scalable alternative or adjunct to biologic agents.

The synergy between PC and CTLA-4 antibodies underscores the importance of cotargeting multiple immune checkpoints to achieve robust antitumor immunity. By degrading PD-L1 via the LKB1/AMPK/SYVN1 axis, PC mitigate the PD-1/PD-L1 inhibitory pathway, while CTLA-4 blockade augments T cell priming and activation. This combination resulted in enhanced CD8^+^ T cell infiltration, reduced Treg and MDSC populations, and M1 macrophage polarization in our models, recapitulating the benefits observed with anti–PD-L1/anti–CTLA-4 regimens. Furthermore, the efficacy of PC in the AOM/DSS-induced CRC model highlights its dual antiinflammatory and immunostimulatory properties (70), potentially addressing the unmet need for therapies for inflammation-driven cancers.

Despite these promising findings, our study has several limitations. Comprehensive pharmacokinetic and pharmacodynamic analyses are needed to optimize PC dosing and evaluate bioavailability in humans. The potential off-target effects of PC and impact on other immune checkpoints require further investigation. Additionally, future studies should validate LKB1 and SYVN1 as biomarkers for patient stratification in clinical trials.

In summary, our findings delineate a potentially unique small-molecule mechanism of immune checkpoint modulation wherein PC co-opt the ERAD pathway to direct PD-L1 for proteasomal destruction. We establish that PC orchestrates this process through dual targeting: It stabilizes the tumor suppressor LKB1 to initiate an AMPK phosphorylation cascade that flags PD-L1 for ER retention while concurrently increasing the protein stability of SYVN1 to execute its ubiquitination and degradation. This concerted action results in robust, T cell–dependent antitumor immunity and exhibits synergistic efficacy with CTLA-4 blockade. The substantial activity of PC in a model of inflammation-driven colorectal carcinogenesis, coupled with its favorable toxicity profile, underscores its potential as a therapeutic agent. Our work not only identifies PC as a promising candidate for clinical translation but also validates the targeting of the LKB1/AMPK/SYVN1 axis as a viable strategy to overcome immune evasion, particularly for cancers reliant on PD-L1 stabilization.

## Methods

### Sex as a biological variable.

This study utilized animal models of both sexes. The sex of the animals was specifically selected as a critical biological variable to optimize the reliability and reproducibility of each distinct disease model. Female mice were used for the subcutaneous tumor model to exclude potential confounding effects of androgen fluctuations and to prevent fighting injuries among male cohorts, thereby ensuring a more stable baseline for intervention assessment (71). In contrast, the AOM/DSS-induced colitis-associated tumor model employed male mice, as they demonstrate heightened sensitivity to the induction protocol, which consistently yields more severe colitis and robust tumor development, a standard practice in the field to ensure model success (72, 73). This deliberate experimental design maximizes the robustness of each model. Furthermore, the demonstration of PC’s efficacy across both models in both sexes indicates that the therapeutic findings is not influenced by sex.

This study utilized human tissue samples obtained from both male and female donors. However, sex was not considered as a biological variable in the experimental design or data analysis. The samples were pooled and analyzed without stratification by sex. Consequently, while the findings are derived from a mixed-sex cohort, we are unable to draw specific conclusions regarding potential sex-based differences. The generalizability of the results to both sexes is assumed but was not explicitly tested in this investigation.

### Western blotting and immunoprecipitation.

Cell lysates were subjected to BCA assay for protein quantification. Protein isolates were then separated by SDS-PAGE, transferred to PVDF membranes (electrophoresed at 80 V and electrotransferred at 250 mA), blocked with 5% skim milk in TBS-Tween for 1 hour, incubated overnight with primary antibody at 4°C, incubated with secondary antibody for 1 hour at room temperature, and visualized using the ChemiDoc imaging system (Bio-Rad, catalog 1708280 (6, 60). For immunoprecipitation (IP) (51), cells were lysed in IP lysis buffer with 1% PMSF for 30 minutes, then centrifuged at 12,000*g* (Eppendorf) at 4°C for 15 minutes, and the resulting lysates were quantified. A total of 500 μg of protein lysate was incubated with anti–PD-L1 or HRD1 antibodies overnight at 4°C, followed by pulldown with protein A/G magnetic beads for 3 hours at 4°C. The beads were washed with lysis buffer, resuspended in 2× loading buffer, heated at 95°C for 10 minutes, and analyzed by Western blotting. The relevant antibodies are shown in Supplemental Table 4.

### T cell killing assay.

RKO or H1975 cells (3 × 10^6^ per well) were plated in 12-well plates. After 9 hours (RKO) or 12 hours (H1975) of exposure to PC, PHA (1 mg/mL)/PMA (50 ng/mL)–activated, PD-1–overexpressing Jurkat cells (courtesy of the Kongming Wu group, Tongji Hospital, Tongji Medical College, Huazhong University of Science and Technology, Wuhan, China) were added at a 9:1 effector-to-target ratio and cocultured for an additional 24 hours. The surviving tumor cells were fixed with 4% paraformaldehyde, stained with 0.5% crystal violet, and imaged using a Cytation 5 instrument (Bio-Rad).

### CETSA.

RKO cells (≥80% confluence) from 4 dishes were lysed on ice for 30 minutes with IP lysis buffer containing protease inhibitors. After centrifugation at 12,000*g* (Eppendorf) at 4°C for 15 minutes, the supernatant (total protein) was collected, divided into 2 equal aliquots, and then incubated with either 200 μM PC or DMSO at 4°C for 1 hour. These samples were then divided into 60 μL aliquots and heated at various temperatures for 3 minutes. After cooling, samples were centrifuged at 12,000*g* (Eppendorf) for 10 minutes at 4°C, the supernatant was collected, and target protein levels were analyzed via Western blotting (55, 74).

### Molecular docking and MST.

The chemical structure of PC was obtained from the PubChem database (https://pubchem.ncbi.nlm.nih.gov/), and its energy was minimized using the MOE software (48). The crystal structure of HRD1 was retrieved from the literature and imported into MOE (49, 60, 75). Molecular docking simulations were performed using the DOCK module in MOE, and the binding mode with the highest docking score was selected as the final covalent binding structure. Based on this structure, GFP-HRD1 and mutant plasmids were constructed. Specifically, the synthesized cDNA was inserted into the pcDNA3.1 vector to generate an expression vector encoding pcDNA3.1-GFP-HRD1. The mutant plasmid was created via site-directed mutagenesis PCR. The resulting plasmid was sequenced to confirm the correct mutation. The plasmid was subsequently transfected into HEK293T cells (Supplemental Table 3), which were collected after 48 hours. Cell lysates were prepared by adding 1% protease inhibitor to the IP lysate. The lysed protein solution was then collected and analyzed via Monolith NT.115 MST device (NanoTemper) (60).

### Animal experiments.

For the subcutaneous tumor model (76–78), 8-week-old C57BL/6 mice were subcutaneously inoculated with MC38 cells (1 × 10^7^) or Lewis cells (3 × 10^7^). When average tumor volume reached 50 mm^3^, mice were treated with different PC concentrations via gavage or intraperitoneal anti–PD-L1 (100 μg) or anti–CTLA-4 (100 μg). Antibody groups were injected every 6 days (2 total doses); PC group received daily gavage. Tumor volume and body weight were measured every 2 days to ensure tumors ≤ 1,600 mm^3^. About 2 weeks after treatment start, mice were euthanized. Major organs (heart, liver, spleen, lung, kidney) and tumors were fixed in 4% paraformaldehyde and sent to Shanghai Yangming Biotech for H&E staining and immunohistochemical analysis.

For the AOM/DSS model (79–81), 8-week-old male C57BL/6 mice were used to establish AOM/DSS-induced CRC. Before the experiment, mice were weighed, labeled, grouped, and confirmed healthy. Mice received intraperitoneal AOM (12.5 mg/kg), then conventional housing with regular water for 1 week before DSS. Sterile 2.5% DSS replaced water for 1 week to induce colitis, then regular water for 2-week recovery. Three DSS-recovery cycles (1-week DSS + 2-week recovery) were done to mimic human ulcerative colitis carcinogenesis. PC (50 mg/kg) gavage started at the end of the second cycle; mice were weighed weekly to monitor health. After 3 cycles, mice were sacrificed when obvious CRC lesions appeared. Colons were harvested for H&E (to assess histopathology and lesion extent), immunohistochemistry (to detect cancer markers), and length/weight measurements (to evaluate tumor burden).

### Tumor-infiltrating lymphocyte detection.

Tumor tissues were minced into small pieces and subjected to enzymatic digestion for 1 hour at 37°C in a shaker, using a digestion solution containing type IV collagenase and DNase I (51, 76, 78, 81). The digested tissue was then filtered through a 40 μm cell strainer to remove any undigested tissue debris. The samples were centrifuged at 200*g* for 4 minutes at room temperature, using an Eppendorf centrifuge and resuspended in PBS to obtain the tumor-infiltrating lymphocyte suspension. Next, the cells were incubated with a panel of surface marker antibodies, including CD45, CD3, CD4, CD8, CD25, CD11b, F4/80, CD19, CD161, and Gr-1, for 30 minutes at 4°C. The cells were then washed with PBS and centrifuged (Eppendorf) at 200*g* for 4 minutes at room temperature to remove the unbound antibodies. The cells were subsequently treated with a nucleolytic reagent for 30 minutes at 4°C to permeabilize the nuclei. This was followed by staining with intracellular antibodies targeting GzmB, Foxp3, CD86, and CD206 for an additional 30 minutes at 4°C. After these steps, the cells were ready for analysis by flow cytometry (Beckman Coulter) to detect the expression of the relevant markers.

### Clinical tissue samples and bioinformatics analysis.

Human colon cancer and adjacent tissues were obtained from Longhua Hospital of Shanghai University of Traditional Chinese Medicine. Lung cancer samples from patients treated with neoadjuvant immunotherapy were obtained from Beijing Shijitan Hospital, Capital Medical University, Beijing, China. All the samples were collected after patients provided informed consent. The clinical information is summarized in Supplemental Tables 1 and 2. In addition, tumor-infiltrating immune cell levels in patients with READ, COAD, and LUAD were calculated via the TIMER and MCPcounter algorithms (82–84).

### Statistics.

Graphical summaries and flowcharts of the animal experiments were generated using BioRender and Adobe Illustrator software, respectively. Quantitative image analysis was conducted using ImageJ-win64 software, and statistical analyses were performed using GraphPad Prism 10 software. Data are presented as mean ± SEM. Independent samples 2-tailed *t* tests were used for comparisons between 2 groups, and 1-way ANOVA or 2-way ANOVA was employed for comparisons between multiple groups. Bioinformatics analyses were performed within the R 4.1.1 environment. Statistical significance was defined as P < 0.05.

### Study approval.

All animals in this study were purchased from Shanghai Ji Hui Laboratory Animal Feeding Co., Ltd., and all animal experiments followed ethical guidelines of and were approved by the Department of Laboratory Animal Science, Shanghai University of Traditional Chinese Medicine (approval: PZSHUTCM2310160001). The human samples used in this research were obtained and analyzed in accordance with the guidelines approved by the Ethics Committee of Beijing Shijitan Hospital under the approval number sjtkyll-lx-2022(35). All participants provided written informed consent.

### Data availability.

The data supporting the findings of this study are available within the article and its supplement. Additional methods are in Supplemental Methods. Source data for all graphs and quantitative image analyses have been provided as a Supporting Data Values Excel file.

## Author contributions

S Liu, WZ, QW, Hanchen Xu, and X Liu participated in conceptualization, original draft, methodology, review and editing, funding acquisition, and supervision. M Xu analyzed the data, carried out the experiments, generated the figures, and wrote the paper. X Lin, Hanchi Xu, HH, XX, QZ, DY, ST, Hanchen Xu, M Xie, LL, XT, X Li, S Li, SX, and YT participated in part of the experiments.

## Funding support

- National Natural Science Foundation of China (82374086, 82574633, 82141203, 82104459).
- National Key Research and Development Program of China (2022YFC3502000).
- Shanghai Municipal Science and Technology Major Project (ZD2021CY001).
- Three-year Action Plan for Shanghai TCM Development and Inheritance Program [ZY (2021-2023)-0401].
- Innovation Team and Talents Cultivation Program of National Administration of Traditional Chinese Medicine (ZYYCXTDD-202004).
- Science and Technology Commission of Shanghai Municipality (20YF1458700).
- Organizational Key Research and Development Program of Shanghai University of Traditional Chinese Medicine (2023YZZ02).
- Chenguang Program of Shanghai Education Development Foundation and Shanghai Municipal Education Commission (23CGA45, SS.T.).
- CAMS Innovation Fund for Medical Sciences (2023-I2M-3-009).
- Key Project at Central Government Level: The Ability Establishment of Sustainable Use for Valuable Chinese Medicine Resources (2060302-2305-02).

## Supplementary Material

Supplemental data

Unedited blot and gel images

Supporting data values

## Version 1 02/02/2026

Electronic publication
