# Expression of Concern for Proanthocyanidins enhance antitumor immunity by promoting ubiquitin-proteasomal PD-L1 degradation via stabilization of LKB1 and SYVN1

**DOI:** 10.1172/JCI213313

**Published:** 2026-10-15

**Authors:** Mengting Xu, Xuwen Lin, Hanchi Xu, Hongmei Hu, Xinying Xue, Qing Zhang, Dianping Yu, Saisai Tian, Mei Xie, Linyang Li, Xiaoyu Tao, Xinru Li, Simeng Li, Shize Xie, Yating Tian, Xia Liu, Hanchen Xu, Qun Wang, Weidong Zhang, Sanhong Liu

Original citation: J Clin Invest. 2026;136(3):e197592. https://doi.org/10.1172/JCI197592

Citation for this expression of concern: J Clin Invest. 2026;136(20):e213313. https://doi.org/10.1172/JCI213313

The authors recently alerted the Editors to technical artifacts in the data in Figure 2M, Figure 4D, and Supplemental Figure 5A that were identified in response to an institutional mandate for a post-publication audit of the data. Although the authors have indicated that independent lines of investigation support the article’s conclusions, the Editors are issuing this Expression of Concern to alert readers to the issues. We have requested institutional oversight into this matter and will alert readers to the outcome when the institutional evaluation is complete.

## Version 1 10/15/2026

Electronic publication

