# Supplementary material for: Proanthocyanidins enhance antitumor immunity by promoting ubiquitin-proteasomal PD-L1 degradation via stabilization of LKB1 and SYVN1
Source: J Clin Invest. 2026 Feb 2;136(3):e197592. doi: 10.1172/JCI197592 (PMC12867145; doi:10.1172/JCI197592)
Supplement: Supplemental data [file jci-136-197592-s033.pdf]

Supplementary Materials for

Proanthocyanidins enhance antitumor immunity by promoting ubiquitin-proteasomal PD-L1 degradation via stabilization of LKB1 and SYVN1

Mengting Xu[1,2], Xuwen Lin[3], Hanchi Xu[2], Hongmei Hu[1,2], Xinying Xue[3], Qing Zhang[2], Dianping Yu[2], Saisai Tian[4], Mei Xie[3], Linyang Li[2], Xiaoyu Tao[2], Xinru Li[2], Simeng Li[2], Shize Xie[4], Yating Tian[4], Xia Liu[4*], Hanchen Xu[1*], Qun Wang[2*], Weidong Zhang[4,5*], Sanhong Liu[1,2*]

Corresponding to: Sanhong Liu (liush@shutcm.edu.cn), Weidong Zhang (wdzhangy@hotmail.com), Qun Wang (qunwang0523@163.com), Hanchen Xu (hanson0702@126.com), Xia Liu (lxflying@aliyun.com)

This PDF file includes:

Figures S1 to S11

Tables S1 to S6

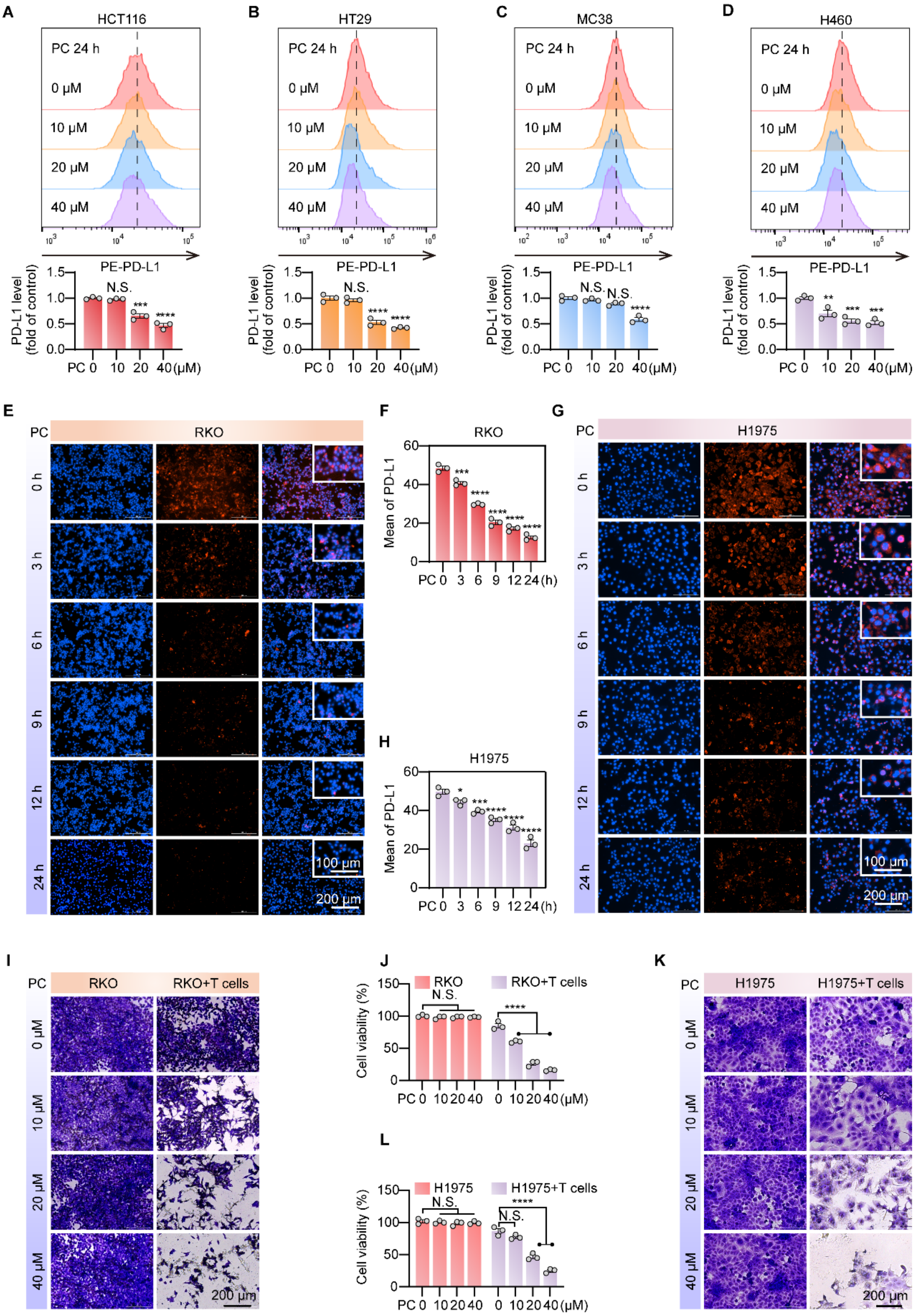

Figure S1. PC significantly downregulates PD-L1 expression. (A-D) Flow cytometry was performed to assess the impact of various PC concentrations on PD-L1 surface expression in HCT116 (A), HT29 (B), MC38 (C), and H460 (D) cell lines. **(E-**

H) Immunofluorescence analysis was conducted to examine PD-L1 expression on RKO (E) and H1975 (G) cell membranes at different time points. PD-L1 is indicated by red fluorescence, and nuclei are stained blue; scale bar = 200 μm. (F) and (H) represent quantitative analyses of (E) and (G), respectively. **(I-L)** PBMCs were cultured for seven days in CTSTM AIIM VTM SFM (Gibco) supplemented with 1000 U/mL recombinant human IL-2 and human CD3/CD28/CD2 T cell activator (Stemcell Technologies) to obtain activated T cells. RKO (I) and H1975 (K) cells were seeded, treated with the indicated conditions of our compound PC for 24 h, and then co-cultured with the activated primary T cells for 24 h at an effector-to-target ratio of 3:1. After washing away debris and T cells, the remaining viable tumor cells were stained with crystal violet and imaged via a Cytation 5. (J) and (L) are quantitative plots representing the data from (I) and (K), respectively. The data shown are the mean ± SEM of triplicate experiments. Statistical differences were determined by One-Way ANOVA with Dunnett's multiple-comparison test. *p < 0.05, **p < 0.01, ***p < 0.001, and ****p < 0.0001. N.S., not significant.

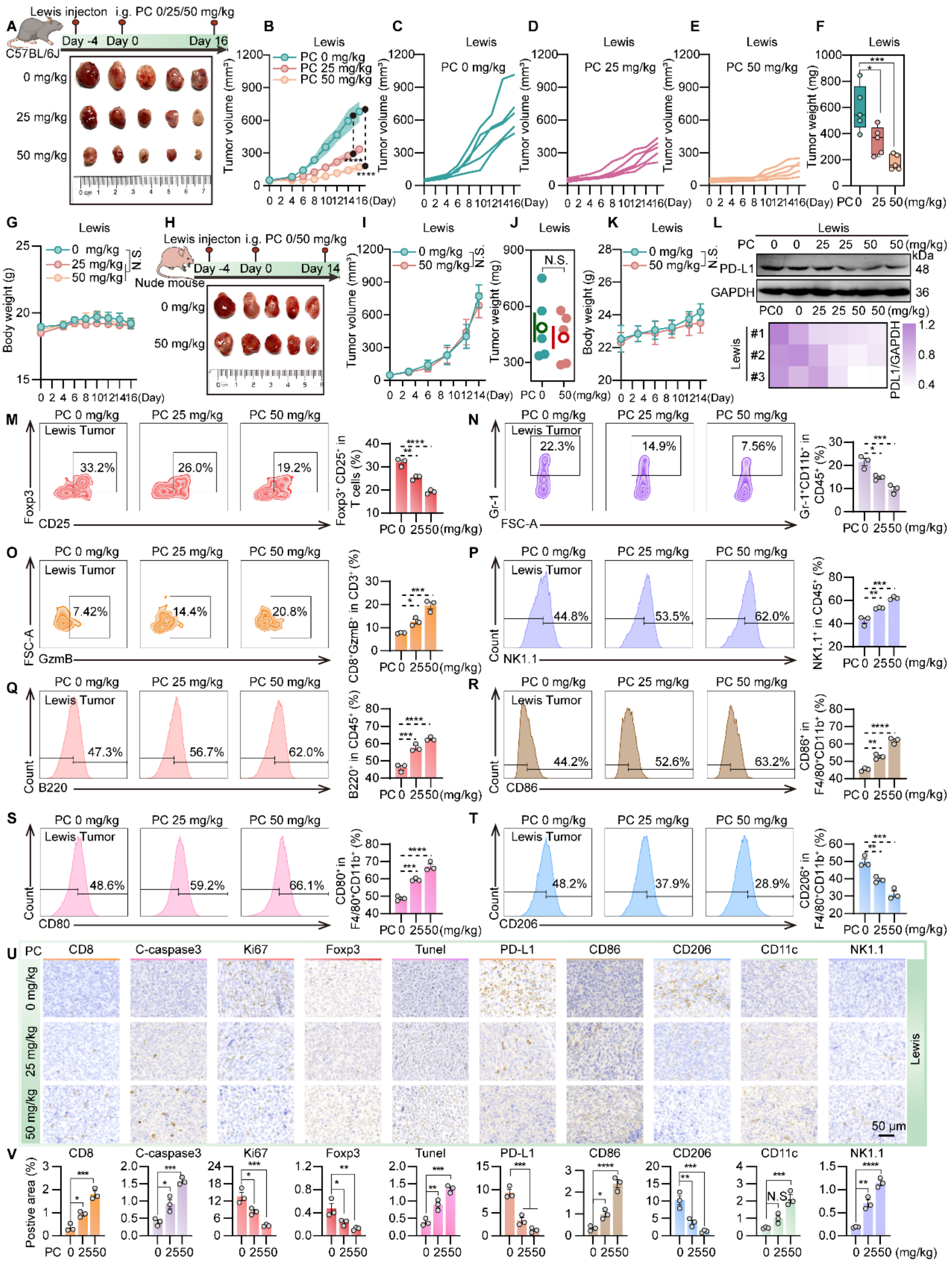

Figure S2. PC attenuates Lewis tumor growth in C57BL/6 mice. C57BL/6 mice or nude mice were subcutaneously implanted with $3×10^7$ Lewis lung cancer cells. Upon reaching a tumor volume of approximately 50 mm^3, the compound of interest (PC) was administered via oral gavage as a treatment. n = 5 mice per group. The tissue samples were harvested approximately two weeks after the initiation of treatment. **(A)**

Experimental flowchart and tumor schematic in C57BL/6 mice. **(B-E)** Tumor growth curves in C57BL/6 mice treated with different doses of PC, specifically showing 0 (C), 25 (D), and 50 (E) mg/kg. **(F)** Statistical analysis of subcutaneous tumor weights in C57BL/6 mice. **(G)** C57BL/6 mice body weight changes. **(H-K)** In nude mice bearing Lewis subcutaneous tumors (H), 50 mg/kg PC showed no effect on tumor growth (I), tumor weight (J), or body weight (K) versus the 0 mg/kg control. Further analyses were conducted on C57BL/6 mice tumors: **(L)** Western blot detection of PD-L1 protein expression; **(M-T)** Flow cytometry analysis of tumor-infiltrating lymphocytes identified the following populations: Tregs (M, $CD4^+CD25^+Foxp3^+$), MDSCs (N, $CD11b^+Gr\text{-}1^+$ in $CD45^+$), cytotoxic T cells (O, $CD8^+GzmB^+$), NK cells (P, $NK1.1^+$), B cells (Q, $B220^+$), M1 macrophages (R, S; $CD86^+$ and $CD80^+$), and M2 macrophages (T, $CD206^+$). **(U and V)** Immunohistochemical analysis of Lewis tumors from PC-treated C57BL/6 mice quantified multiple markers—including CD8, cleaved caspase-3, Ki-67, Foxp3, TUNEL, PD-L1, CD86, CD206, CD11c, and NK1.1—across three representative tumor regions. (V) Positive staining was quantified using ImageJ and statistically analyzed with GraphPad Prism. The data shown are the mean ± SEM. Statistical differences were determined by ANOVA with Dunnett's multiple-comparison test; and unpaired 2-tailed Students' t test. *$p < 0.05$, **$p < 0.01$, ***$p < 0.001$, and ****$p < 0.0001$. N.S., not significant.

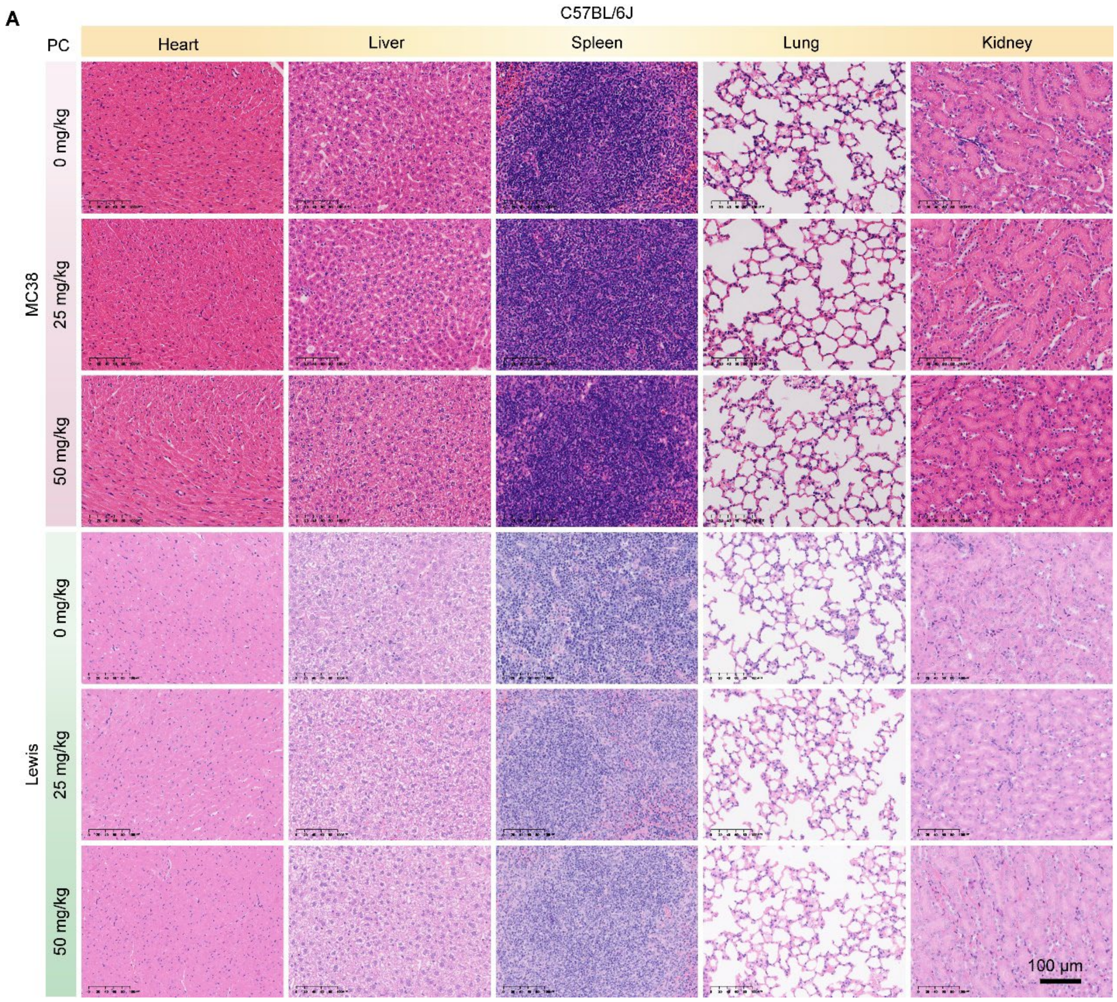

Figure S3. PC does not damage the viscera of C57BL/6 mice. MC38 colon cancer cells or Lewis lung cancer cells were injected subcutaneously into C57BL/6J (female) mice treated orally with different doses of PC (n = 5 mice per group). H&E staining of the heart, liver, spleen, lungs and kidneys of different groups of C57BL/6J mice.

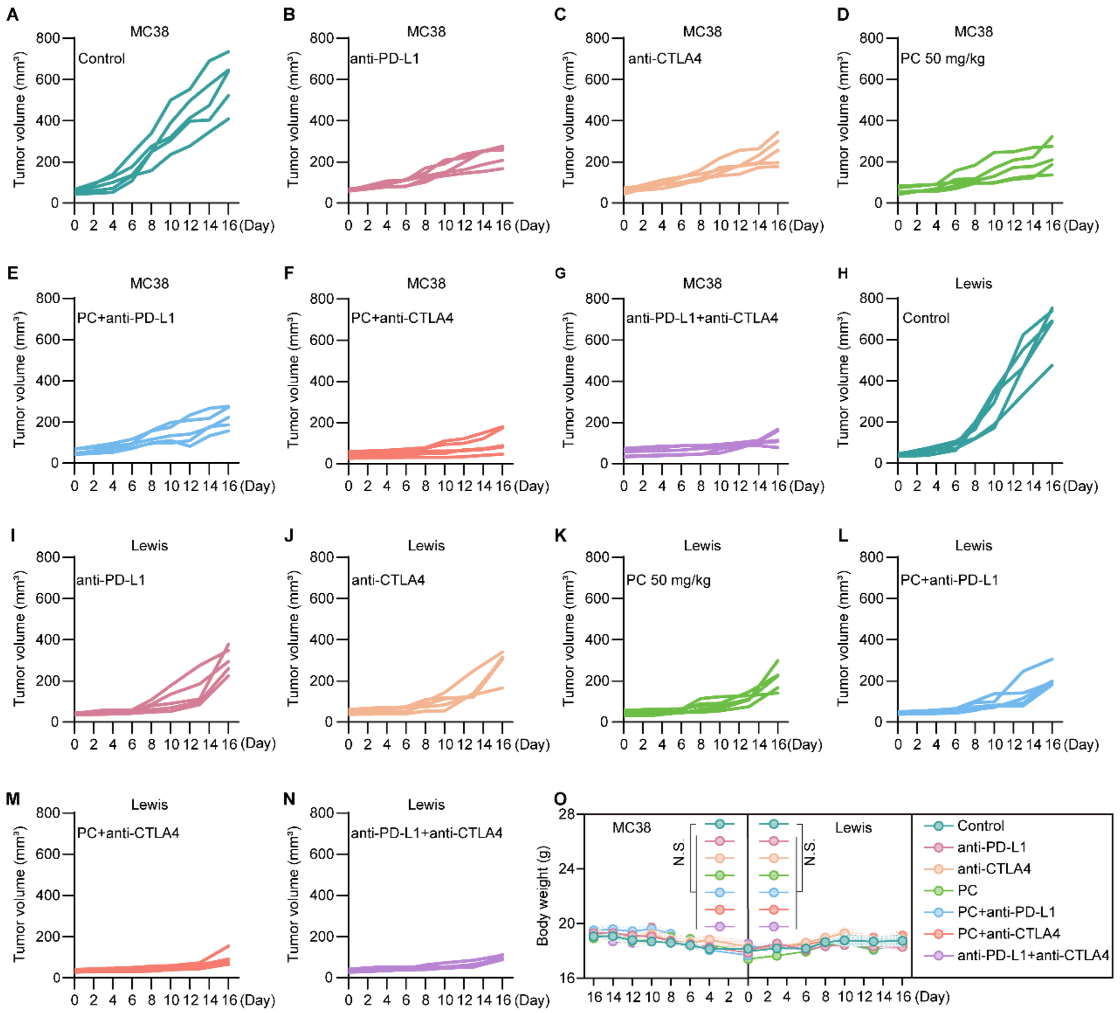

Figure S4. PC combined with anti-CTLA4 enhances tumor growth inhibition in colon and lung cancer. Statistical growth curves of tumors from C57BL/6 mice subcutaneously transplanted with MC38 (1×10^7 cells) or Lewis (3×10^7 cells) after treatment with different drugs. n = 5 mice per group. **(A-G)** Tumor growth curves of C57BL/6 mice subcutaneously transplanted with MC38 or Lewis cells and treated with PBS (A), anti-PD-L1 (B), anti-CTLA4 (C), PC (D), PC + anti-PD-L1 (E), PC + anti-CTLA4 (F), or anti-PD-L1 + anti-CTLA4 (G). **(H-N)** Tumor growth curves of C57BL/6 mice subcutaneously transplanted with MC38 cells and treated with PBS (H), anti-PD-L1 (I), anti-CTLA4 (J), PC (K), PC + anti-PD-L1 (L), PC + anti-CTLA4 (M), or anti-PD-L1 + anti-CTLA4 (N). (O-P) Body weight changes in MC38-transplanted (O) and Lewis-transplanted (P) mice treated with different regimens. The data shown are the mean ± SEM. Statistical differences were determined by Two-way ANOVA with

Dunnett's multiple-comparison test. *p < 0.05, **p < 0.01, ***p < 0.001, and ****p < 0.0001. N.S., not significant.

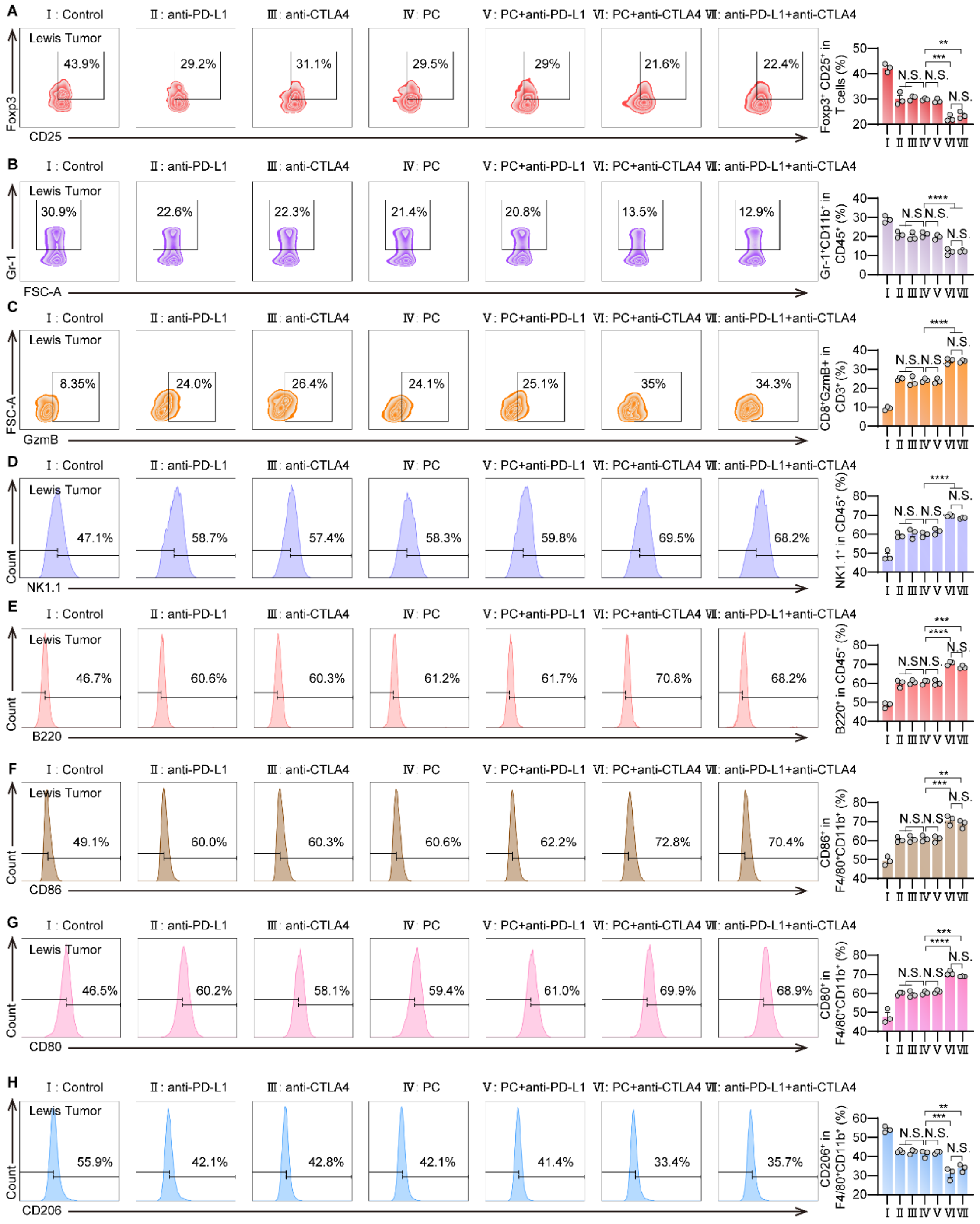

Figure S5. The combination of PC and anti-CTLA4 enhances tumor-associated lymphocyte infiltration in Lewis tumors. (A-H) Flow cytometry analysis of tumor-associated lymphocyte markers in tumor tissues from Lewis-transplanted mice treated with various regimens. Mice transplanted with Lewis (3×10^7 cells) were treated with

PBS, anti-PD-L1 (100 μg), anti-CTLA4 (100 μg), PC (50 mg/kg), PC + anti-PD-L1, PC + anti-CTLA4, or anti-PD-L1 + anti-CTLA4 (n = 5 mice per group). Panels (A) to (H) represent the proportions of $CD4^+$ $CD25^+$ $Foxp3^+$, $CD11b^+$ $Gr\text{-}1^+$, granzyme B^+, $NK1.1^+$, $B220^+$, $CD86^+$, $CD80^+$, and $CD206^+$ cells in tumor tissues from each treatment group, respectively. Data were statistically analyzed. The data shown are the mean ± SEM. Statistical differences were determined by Two-way ANOVA with Tukey's multiple-comparison test. *$p < 0.05$, **$p < 0.01$, ***$p < 0.001$, and ****$p < 0.0001$. N.S., not significant.

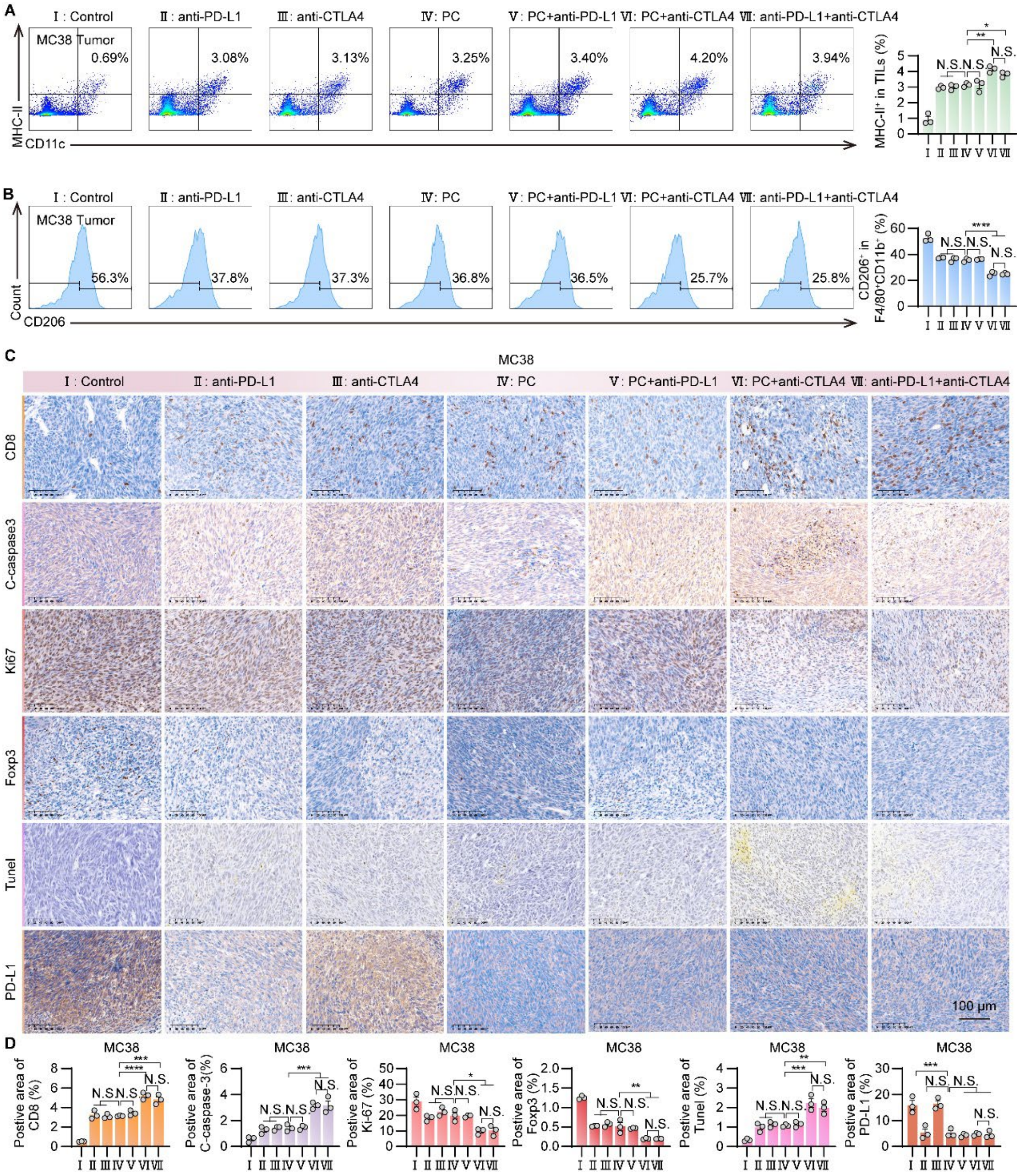

Figure S6. PC in combination with anti-CTLA4 significantly enhanced immune activation in colon cancer mice. C57BL/6 mice subcutaneously transplanted with MC38 (1×10^{7} cells) were treated with seven regimens: PBS, anti-PD-L1 (100 μg), anti-CTLA4 (100 μg), PC (50 mg/kg), PC + anti-PD-L1, PC + anti-CTLA4, and anti-PD-L1 + anti-CTLA4 (n = 5 mice per group). Tumor-associated lymphocyte markers in the tumors of each group were analyzed by flow cytometry and immunohistochemical staining. **(A)** Proportion of $CD11c^{+}MHCII^{+}$ cells in tumor tissues from each group. **(B)** Proportion of $CD206^{+}$ cells in tumor tissues from each group. **(C)** Immunohistochemical detection of CD8, C-caspase-3, Ki-67, Foxp3, TUNEL, and PD-L1 levels in tumors from each group. **(D)** Quantitative statistical analysis of the results shown in (C). The data shown are the mean ± SEM. Statistical differences were determined by Two-way ANOVA with Tukey's multiple-comparison test. *p < 0.05, **p < 0.01, ***p < 0.001, and ****p < 0.0001. N.S., not significant.

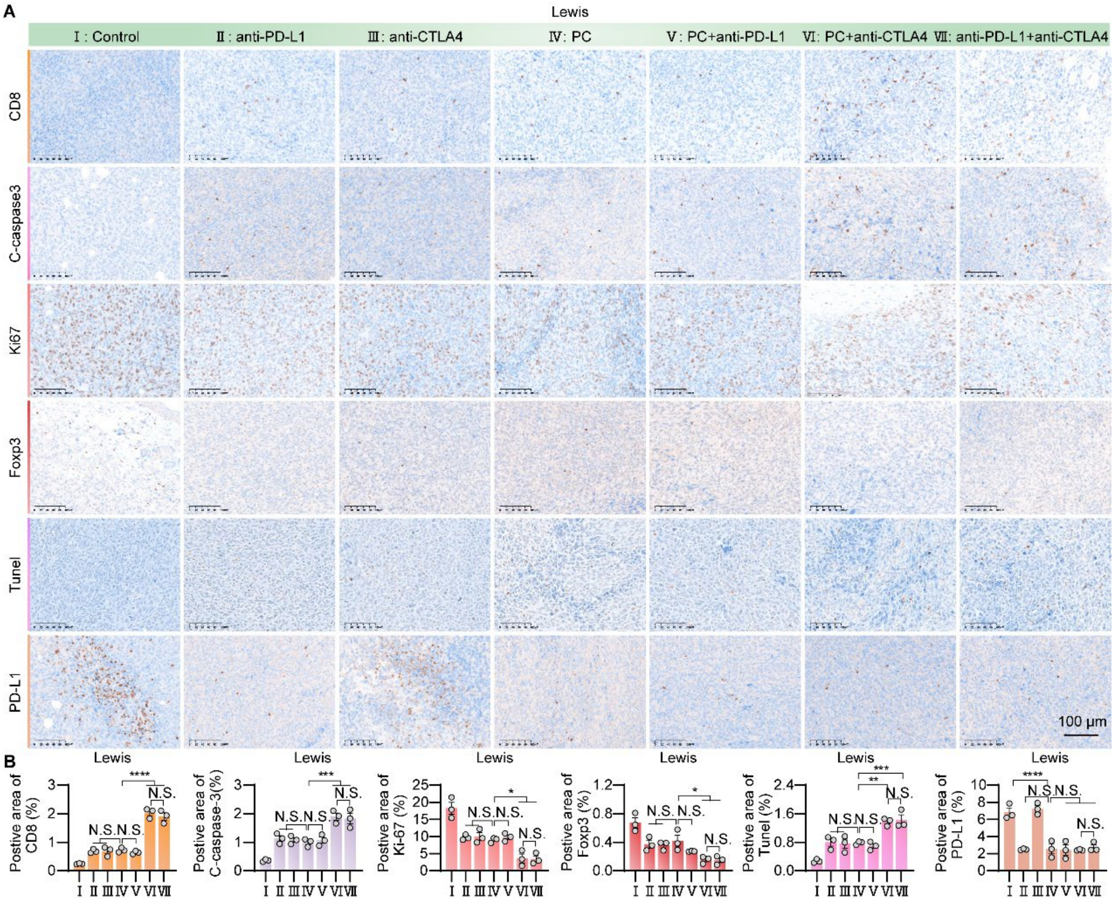

Figure S7. PC in combination with anti-CTLA4 significantly enhanced immune activation in lung cancer mice. C57BL/6 mice subcutaneously transplanted with Lewis (3×10^7 cells) were treated with seven regimens: PBS, anti-PD-L1 (100 µg), anti-CTLA4 (100 µg), PC (50 mg/kg), PC + anti-PD-L1, PC + anti-CTLA4, and anti-PD-L1 + anti-CTLA4 (n = 5 mice per group). Tumor-associated lymphocyte markers in the tumors of each group were analyzed by immunohistochemical staining. **(A)** Immunohistochemical detection of CD8, C-caspase-3, Ki-67, Foxp3, TUNEL, and PD-L1 levels in tumors from each group. **(B)** Quantitative statistical analysis of the results shown in (C). The data shown are the mean ± SEM. Statistical differences were determined by Two-way ANOVA with Tukey's multiple-comparison test. *p < 0.05, **p < 0.01, ***p < 0.001, and ****p < 0.0001. N.S., not significant.

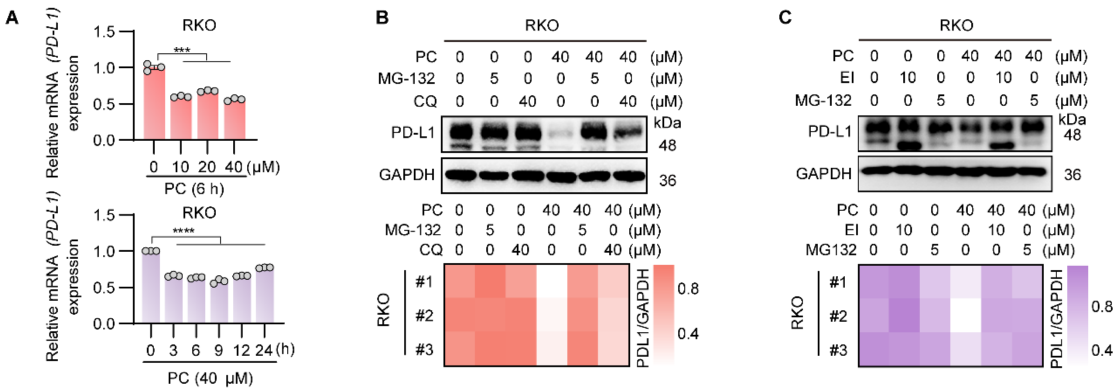

Figure S8. PC induces ubiquitinated degradation of PD-L1 in the endoplasmic reticulum. (A) PD-L1 mRNA levels in RKO cells were measured by RT-qPCR after treatment with different concentrations of PC for a fixed duration or with a fixed concentration of PC for varying durations. **(B)** The protein level of PD-L1 in RKO cells co-treated with 40 µM PC and either 5 µM MG-132 or 40 µM CQ was detected by Western blotting, and the results were quantitatively analyzed. **(C)** PD-L1 protein levels in RKO cells co-treated with 40 µM PC and either 10 µM eeyarestatin I or 5 µM MG-132 were detected via Western blotting, and the results were quantitatively analyzed. The data shown are the mean ± SEM of triplicate experiments. Statistical differences were determined by One-way ANOVA with Dunnett's multiple-comparison test. *p < 0.05, **p < 0.01, ***p < 0.001, and ****p < 0.0001. N.S., not significant.

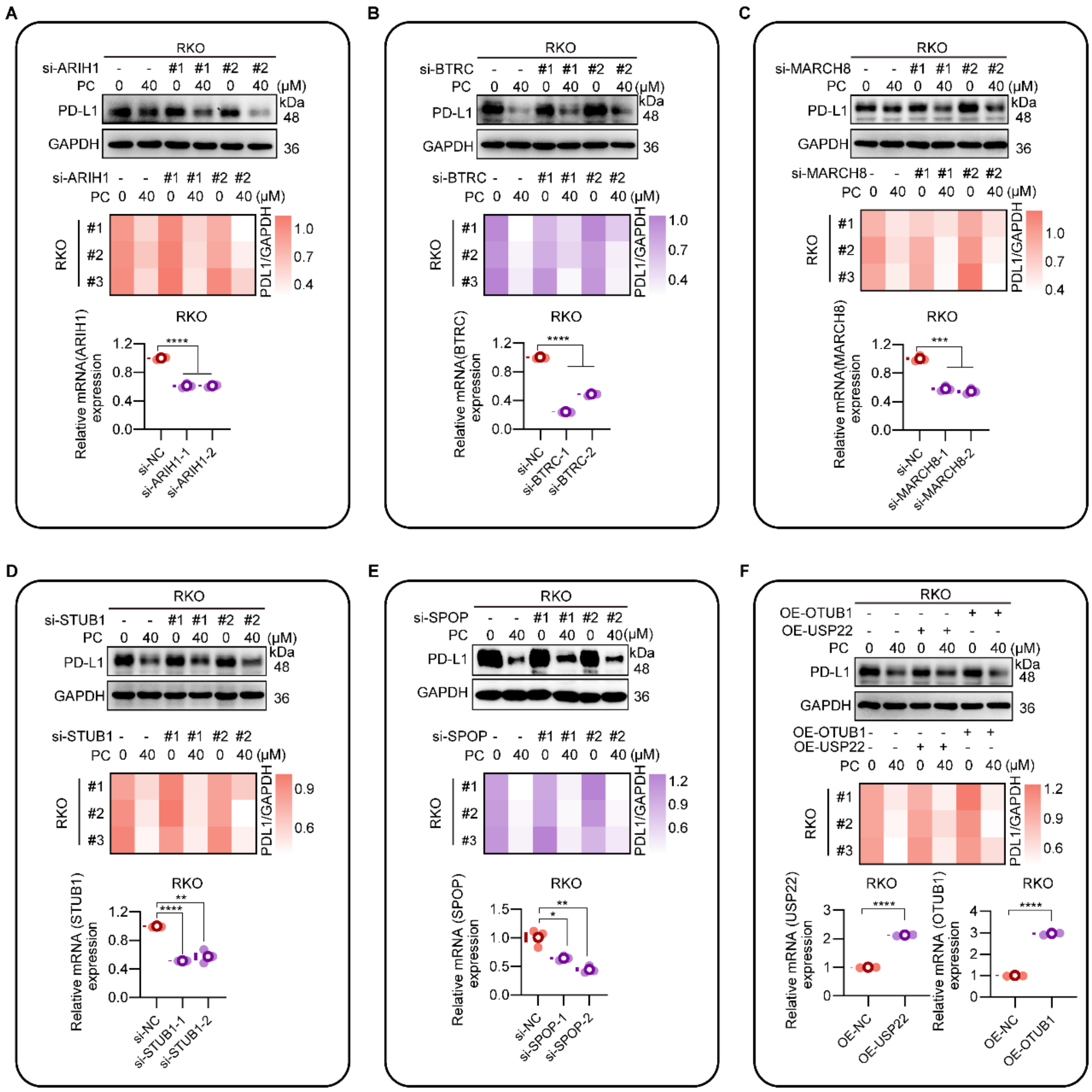

Figure S9. The degradation of PD-L1 by PC was specifically reversed only by SYVN1 knockdown. (A-E) In RKO cells, the expression of ARIH1 (A), BTRC (B), MARCH8 (C), STUB1 (D), and SPOP (E) was selectively silenced via siRNA. The knockdown efficiency was subsequently assessed via RT-qPCR. . Next, the siRNA-treated RKO cells were exposed to PC, and the PD-L1 protein level was determined via Western blotting. The blotting results were then quantified using ImageJ and GraphPad Prism 10 software. **(F)** To investigate whether PD-L1 remained downregulated in RKO cells overexpressing OTUB1 or USP22 after prolonged exposure to PC, we evaluated the knockdown efficiency of these proteins using RT-qPCR. The data shown are the mean ± SEM. Statistical differences were determined by One-way ANOVA with

Dunnett's multiple-comparison test; and unpaired 2-tailed Students' t test. *p < 0.05, **p < 0.01, ***p < 0.001, and ****p < 0.0001. N.S., not significant.

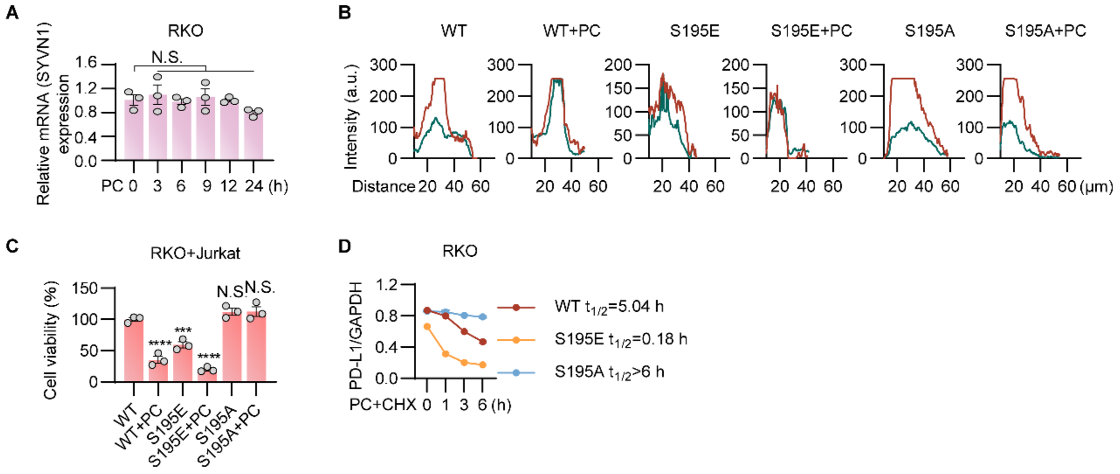

Figure S10. PC regulates AMPK-mediated phosphorylation of PD-L1 at position S195. (A) The mRNA levels of SYVN1 in RKO cells were measured by RT-qPCR after treatment with PC for various durations. **(B-D)** (B), (C), and (D) are the quantitative analyses of Figure 7M, Figure 7N, and Figure 7P, respectively**.** The data shown are the mean ± SEM. Statistical differences were determined by One-way ANOVA with Dunnett's multiple-comparison test. *p < 0.05, **p < 0.01, ***p < 0.001, and ****p < 0.0001. N.S., not significant.

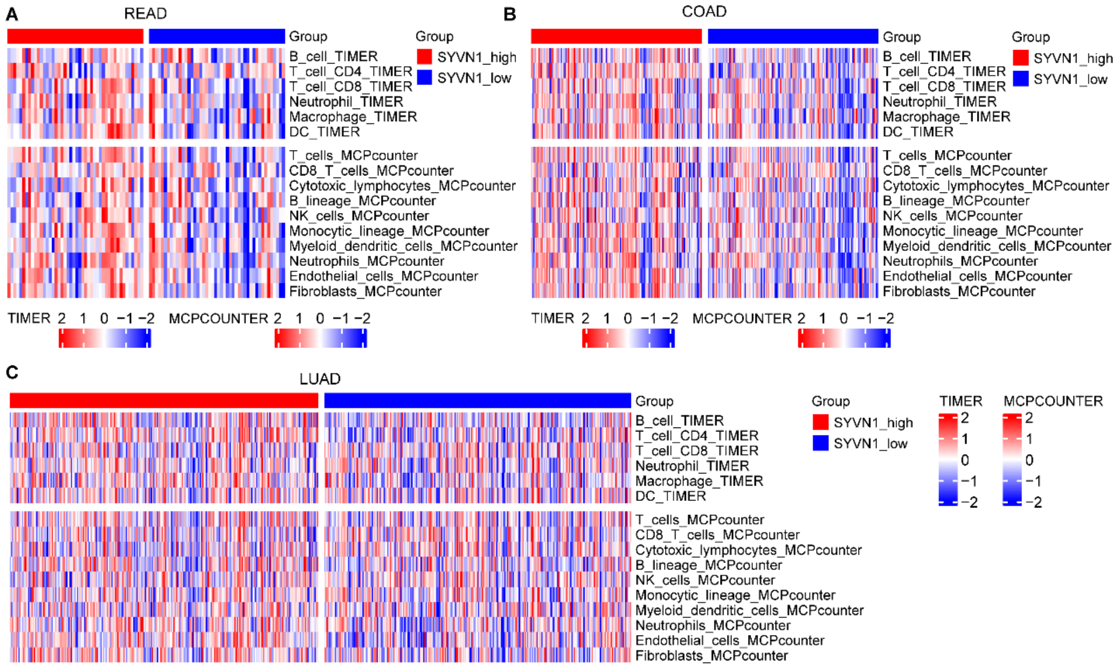

Figure S11. SYVN1 expression and immunotherapy response in cancer. (A-C) Tumor-infiltrating immune cell levels, as determined by the TIMER and MCPcounter algorithms, differed between the high and low SYVN1 expression groups in READ (A), COAD (B), and LUAD (C) patients.

Supplementary Methods

Cell culture and cytotoxicity assays

Cell culture details are as follows: RKO in MEM; HCT116 and HT29 in 5A; H1975, H460 and MC38 in DMEM, and Jurkat-PD-1 (courtesy of the Kongming Wu group, Tongji Hospital, Tongji Medical College, Huazhong University of Science and Technology) and Lewis in RPMI 1640. All media were supplemented with 10% FBS, 100 U/mL penicillin, and 100 mg/mL streptomycin, and cells were maintained at 37°C in 5% CO_2. Cell viability and proliferation were assessed using the Cell Counting Kit-8 and the EdU Cell Proliferation Kit. Refer to Supplementary Table 3 for comprehensive cell and reagent information.

Immunofluorescence co-localization

To detect protein expression or protein co-localization on the cell membrane (1), RKO cells were washed with PBS, fixed with 4% paraformaldehyde for 15 min at room temperature, blocked with 5% bovine serum albumin for 1 hour, and then incubated with primary antibody at 4℃ overnight, followed by incubation with secondary antibody for 1 hour at room temperature. After DAPI nuclear staining, cells were visualized using a Cytation 5 (Bio-Rad, USA). Relevant reagents and antibodies are listed in Supplementary Table 3 and Supplementary Table 4, respectively.

Flow cytometry

Cells were seeded in 12-well plates at 2.5×10^5 cells/well in 1 mL and incubated for 24 h. The medium was then removed, and the plates were washed three times with PBS. Fresh complete medium was added, followed by PC treatment to initiate the experiment. After

treatment, the supernatant was collected, and adherent cells were detached with 0.25% trypsin. Cells from the corresponding groups were pooled and centrifuged at 1200 rpm for 3 min, after which the supernatant was discarded. The cell pellet was resuspended and washed once with PBS. Cells were stained with an antibody diluted in PBS, incubated at 4°C in the dark for 30 min, washed once with 1 mL of PBS, centrifuged, and resuspended in 500 μL of PBS. Finally, the cells were transferred to flow tubes and analyzed via a flow cytometer (Beckman-Coulter, USA) (1-3).

RT-qPCR and transfection

Cells were collected, washed with PBS, and lysed in 1 mL of TRIzol for 5 min. Following addition of 200 μL of chloroform and 15-minute incubation, mixture was centrifuged at 4°C. Supernatant was transferred to a new tube, mixed with isopropyl alcohol, incubated for 15 min, and centrifuged at 4°C. After supernatant was discarded, RNA pellet was washed twice with 75% ethanol, followed by centrifugation and removal of the supernatant. The RNA was resuspended in DEPC water after ethanol evaporation, and its concentration was measured using a Cytation 5 (Bio-Rad, USA). cDNA was then synthesized using a reverse transcription kit, and target RNA levels were determined by LightCycler® 96 system (Roche, Basel, Switzerland), with the reagents listed in Supplementary Table 3. Cells were transfected with plasmids or siRNAs via Lipofectamine 2000, and the medium was changed 8 hours post-transfection. Cells were harvested 48 hours later for RT-qPCR to assess transfection efficiency and Western blotting to examine protein expression. siRNAs and primers were synthesized and validated by GenePharma in Shanghai, China (Supplementary Table 5 and 6).

Table S1. Clinicopathological characteristics of PD-1 mAb therapy in lung cancer, related to Figure 8

Patient No.	Response	Gender	Age	Tumor types	TNM stage	TRG	PS score	Changes in diameter (%)
1	CPR	Male	58	LUSC	T4N2M0	1	1	-100
2	CPR	Male	61	LUSC	T2N2M0	2	0	-47
3	MPR	Male	68	LUSC	T4N2M0	0	0	-31
4	CPR	Male	57	LUSC	T1N2M0	0	0	-100
5	MPR	Male	62	LUSC	T3N2N0	2	1	-96
6	CPR	Male	69	LUSC	T2N2M0	0	0	-25
7	Non-MPR	Famale	53	LUAD	T2N2M0	2	0	-12
8	Non-MPR	Male	69	LUSC	T4N2M0	3	1	-16
9	Non-MPR	Male	69	LUSC	T3N0M0	2	0	-29
10	Non-MPR	Male	55	LUAD	T2N0M0	2	1	-9
11	Non-MPR	Male	41	LUAD	T2N2bM0	2	0	-25
12	Non-MPR	Male	66	LUAC	T4N2M0	3	0	-33

PD-1, programmed death-1; CPR, Complete Pathologic Response; MPR, Major Pathologic Response; Non-MPR, Non-Major Pathologic Response; LUAD, Lung adenocarcinoma; LUSC, lung squamous-cell carcinoma; TRG, tumor regression grade; PS, performance status.

Table S2. Clinicopathological characteristics of PD-1 mAb therapy in lung cancer, related to Figure 8

Patient Characteristics	Responders	Non-responders
	(n=6)	(n=6)
Age (years) mean±SD	62.5±5.010	58.8±11.18
Male	6	5
Female	0	1
Response, n (%)		
CPR	4 (66.7%)	0
MPR	2 (33.3%)	0
Non-MPR	0	6 (100%)

PD-1, programmed death-1; CPR, Complete Pathologic Response; MPR, Major Pathologic Response; Non-MPR, Non-Major Pathologic Response. Patient with CPR and MPR were classified as responders, while patients with Non-MPR was classified as non-responders.

Table S3. Cell lines and Reagents

Cell lines and Reagents	Source
Human colon cancer cell RKO	ATCC
Human colon cancer cell HCT116	ATCC
Human colon cancer cell HT29	ATCC
Human Lung cancer cell H1975	ATCC
Human Lung cancer cell H460	ATCC
Mouse colon cancer cell MC38	ATCC
Mouse Lung cancer cell Lewis	ATCC
MEM	MeilunBio
DMEM	MeilunBio
RPMI1640	MeilunBio
McCOY's 5A	MeilunBio
PBS(1X)	MeilunBio
FBS	Biological Industries
Penicillin, Streptomycin, Glutamine (100X)	MeilunBio
0.25% Trypsin-EDTA Solution with Phenol Red	Beyotime
NP-40 Lysis Buffer	Beyotime
RIPA Lysis Buffer	Beyotime
Cell Complete Lysis Buffer for Western and IP	Beyotime
Phenylmethanesulfonyl fluoride (PMSF)	Beyotime
Cell Counting Kit-8	Beyotime
BeyoClick™ EdU Cell Proliferation Kit with AF488	Beyotime
Antifade Mounting Medium with DAPI	Beyotime
Lipofectamine™ 2000 Transfection Reagent	Invivogen
TRIzol™ Reagent	Takara
SYBR Green SupTaq HS	Accurate Biology
Evo M-MLV Reverse Transcription Kit	Accurate Biology
Proanthocyanidins	Selleck
MG-132	MCE
Chloroquine	MCE
3-Methyladenine	MCE
Cycloheximide	MCE
Eeyarestatin I	MCE
Dorsomorphin dihydrochloride	MCE
Pim1/AKK1-1N-1	MCE

Table S4. Antibody

Antibody	Source	Category No.
PD-L1/CD274 Monoclonal antibody	Proteintech	66248-1-Ig
Anti-PD-L1	Abcam	ab205921
HRD1	Proteintech	13473-1-AP
Ubiquitin	Abcam	ab7245
Anti-AMPK alpha Rabbit mAb	Epizyme	R011752
Phospho-AMPKα (Thr172) (40H9) Rabbit mAb	Cell Signaling Technology	2535T
LKB1 Polyclonal antibody	Proteintech	10746-1-AP
Anti-mouse PD-1 (CD279)	Invivogen	BE0146
Anti-mouse CTLA-4(CD152)	Invivogen	BP0032
GAPDH	Proteintech	60004-1-Ig
PE anti-human CD274	Biolegend	329706
PE anti-mouse CD274	Biolegend	124307
PE anti-mouse CD25	Biolegend	101904
Alexa Fluor® 700 anti-human/mouse Granzyme B Recombinant	Biolegend	372222
Alexa Fluor® 700 anti-mouse CD206 (MMR) Antibody	Biolegend	141734
APC anti-mouse CD80 Antibody	Biolegend	104714
Anti-Mouse CD3ε，APC-Cy7	Multi Sciences	70-F21003A06-100
Anti-Mouse CD8α，PerCP-Cy5.5	Multi Sciences	70-F2100804-100
Anti-Human/Mouse CD11b，mFluor 450	Multi Sciences	70-F41011b07-100
Anti-Mouse CD4，PE-Cy7	Multi Sciences	70-F2100405/2-100
Anti-Mouse Ly-6G(Gr-1)，FITC	Multi Sciences	70-F21LY6G01-100
Anti-Mouse Foxp3，APC	Multi Sciences	70-F21FP303-100
Anti-Mouse F4/80, PE-Cy7	Multi Sciences	70-F21480A05-100
Anti-Mouse MHC ClassII(I-A/I-E), FITC	Multi Sciences	70-F21IIAE01-100
Anti-Mouse CD11c, PE	Multi Sciences	70-F21011C02-100
Anti-Mouse CD19, PE	Multi Sciences	70-F2101902-100
Anti-Mouse NK1.1 (CD161), FITC	Multi Sciences	70-F2116101-100

Table S5. SiRNAs sequences

Name	Sense (5'-3')
si-PD-L1-1	TCAATTGTCATATTGCTAC
si-PD-L1-2	TTGACTCCATCTTTCTTCA
si-SPOP-1	CACAAGGCUAUCUUAGCAGCU
si-SPOP-2	CUCCUACAUGUGGACCAUCAA
si-MARCH8-1	GGACATTTCATGAGTCATT
si-MARCH8-2	GGAAGAGACTCAAGGCCTA
si-BTRC-1	GCGUUGUAUUCGAUUUGAUAA
si-BTRC-2	GCUGAACUUGUGUGCAAGGAA
si-STUB1-1	GCAGUCUGUGAAGGCGCACUU
si-STUB1-2	CCCAAGUUCUGCUGUUGGACU
si-ARIH1-1	CGAGAUAUUUCCCAAGAUUUU
si-ARIH1-2	CCAUGUUGUUAAAGUCCAAUA
si-SYVN1-1	CCAUGAGGCAGUUCAAGAAdTdT
si-SYVN1-2	UGUCUGGCCUUCACCGUUU

Table S6. Primer sequences

Primer name	Primer sequence (5'-3')
Human PD-L1	Forward: GGCATTTGCTGAACGCAT<br>Reverse: CAATTAGTGCAGCCAGGT
Human β-actin	Forward: ATTCCTATGTGGGCGACGAG<br>Reverse: CCAGATTTTCTCCATGTCGTCC
Human SPOP	Forward: GCCAGTGAAATACGAGTTAGGG<br>Reverse: CCTGGAGCGCTTAAAGGTCA
Human MARCH8	Forward: AGTGACATTCCACGTCATTGC<br>Reverse: GATCTCCTCAGCAGTACGGTC
Human BTRC	Forward: TGGCTCATCTGACAACACTATC<br>Reverse: CGAATACAACGCACCAATTCC
Human STUB1	Forward: CGAATACAACGCACCAATTCC<br>Reverse: TCAAGGAGCAGGGCAATCGTCT
Human ARIH1	Forward: GCATCTTCAGGTAGCACAAGGC<br>Reverse: ACTTTGATGGAAACCTGGAGAA
Human SYVN1	Forward: TGCGTAACATCCACACACTG<br>Reverse: CTTTGAGTTTGTATCTTGGATGCC
Human LKB1	Forward: TGTCGGTGGGTATGGACAC<br>Reverse: CCTTGCCGTAAGAGCCTTCC

References

1. Zhang R, Yang Y, Dong W, Lin M, He J, Zhang X, et al. D-mannose facilitates immunotherapy and radiotherapy of triple-negative breast cancer via degradation of PD-L1. Proc Natl Acad Sci U S A. 2022;119(8).
2. Lv C, Huang Y, Wang Q, Wang C, Hu H, Zhang H, et al. Ainsliadimer A induces ROS-mediated apoptosis in colorectal cancer cells via directly targeting peroxiredoxin 1 and 2. Cell Chem Biol. 2023;30(3):295–307.e5.
3. Liu Y, Liu X, Zhang N, Yin M, Dong J, Zeng Q, et al. Berberine diminishes cancer cell PD-L1 expression and facilitates antitumor immunity via inhibiting the deubiquitination activity of CSN5. Acta Pharm Sin B. 2020;10(12):2299–312.
