# Supplementary material for: Proanthocyanidins enhance antitumor immunity by promoting ubiquitin-proteasomal PD-L1 degradation via stabilization of LKB1 and SYVN1
Source: J Clin Invest. 2026 Feb 2;136(3):e197592. doi: 10.1172/JCI197592 (PMC12867145; doi:10.1172/JCI197592)

Figure 1B

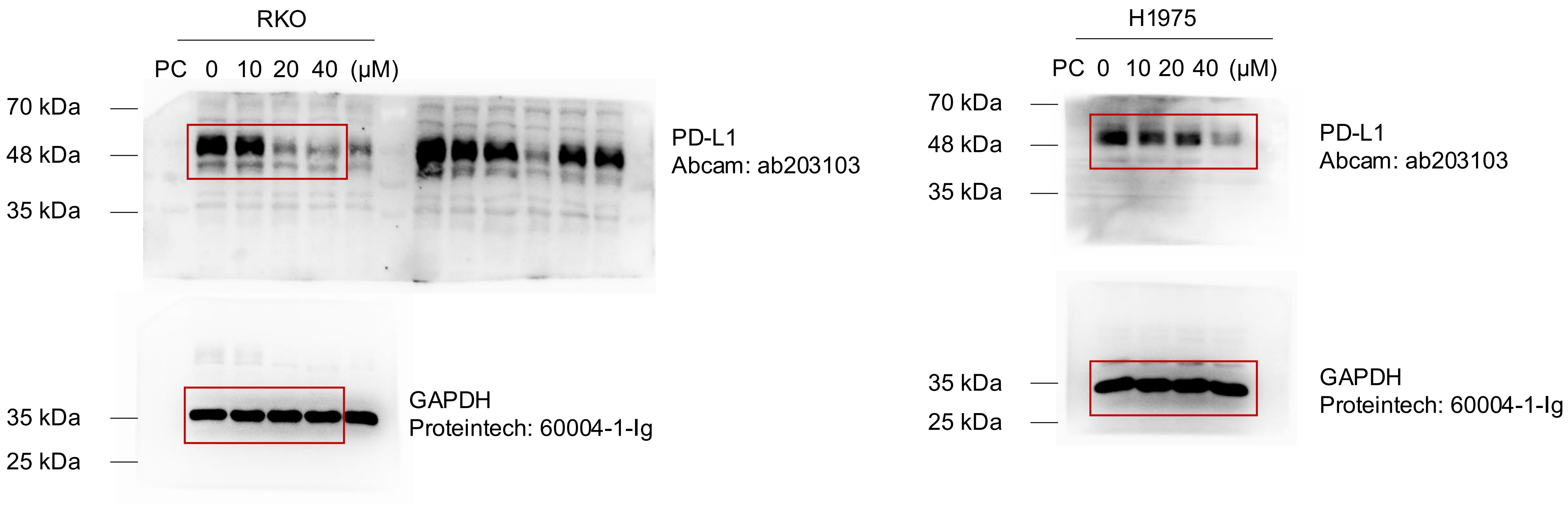

RKO
PC 0 10 20 40 (μM)
70 kDa
48 kDa
35 kDa
PD-L1
Abcam: ab203103
35 kDa
25 kDa
GAPDH
Proteintech: 60004-1-Ig
H1975
PC 0 10 20 40 (μM)
70 kDa
48 kDa
35 kDa
PD-L1
Abcam: ab203103
35 kDa
25 kDa
GAPDH
Proteintech: 60004-1-Ig

Figure 1D

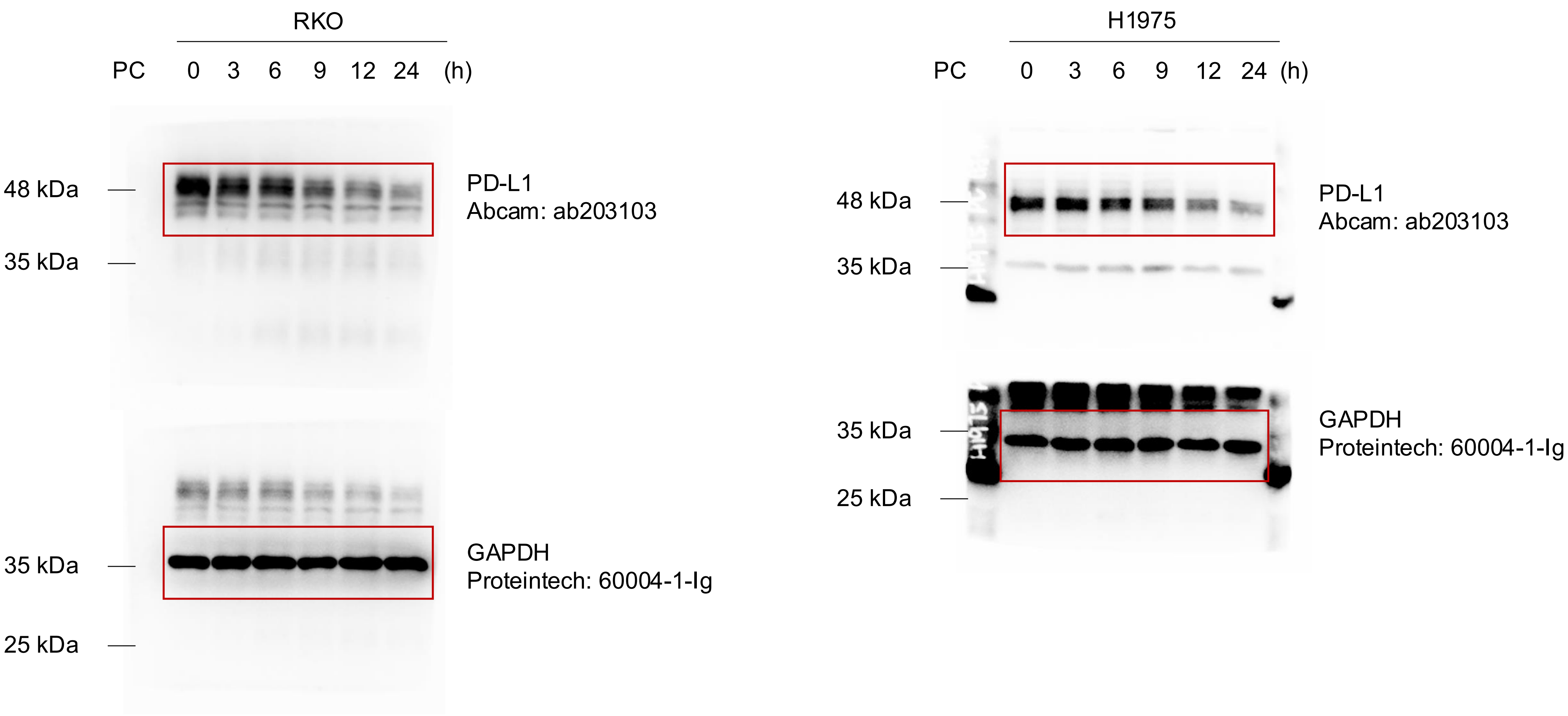
RKO
PC 0 3 6 9 12 24 (h)
48 kDa
35 kDa
PD-L1
Abcam: ab203103
35 kDa
25 kDa
GAPDH
Proteintech: 60004-1-Ig
H1975
PC 0 3 6 9 12 24 (h)
48 kDa
35 kDa
PD-L1
Abcam: ab203103
35 kDa
25 kDa
GAPDH
Proteintech: 60004-1-Ig

Figure 2L

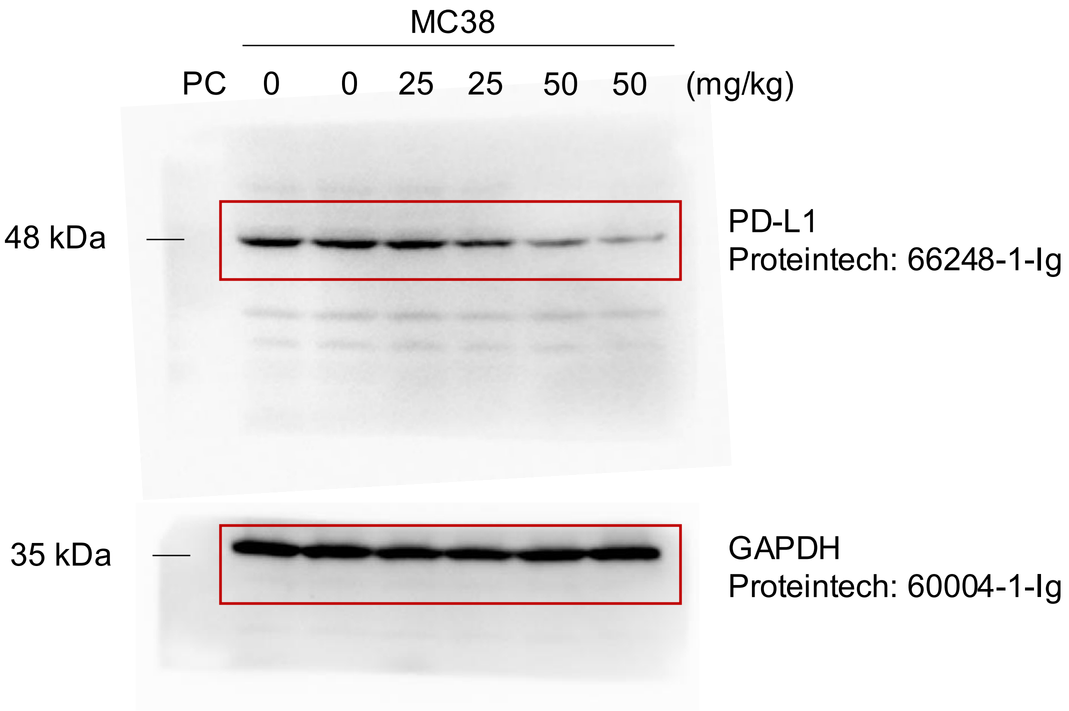
MC38
PC 0 0 25 25 50 50 (mg/kg)
48 kDa
PD-L1
Proteintech: 66248-1-Ig
35 kDa
GAPDH
Proteintech: 60004-1-Ig

Figure 5A

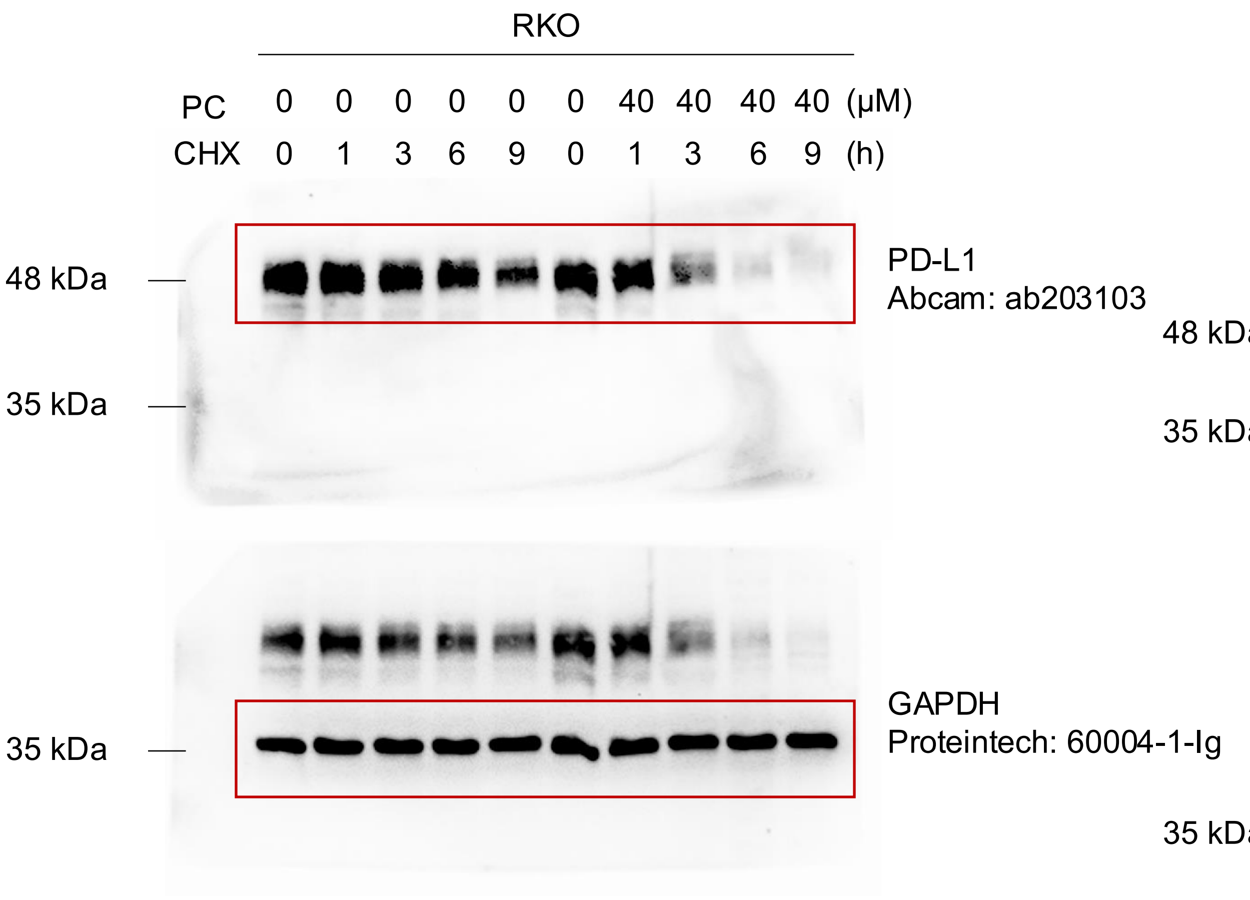

Figure 5D

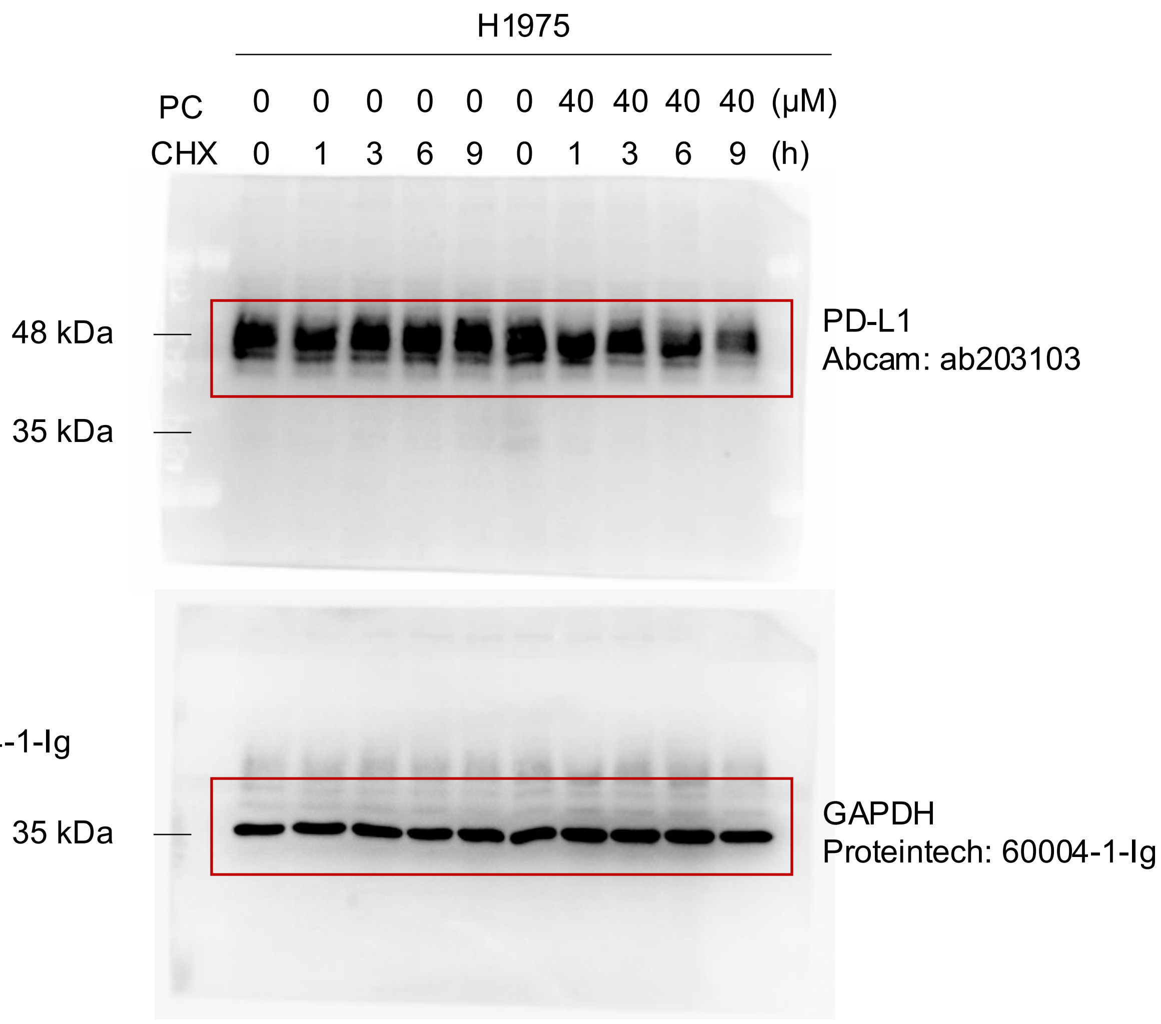

Figure 5E

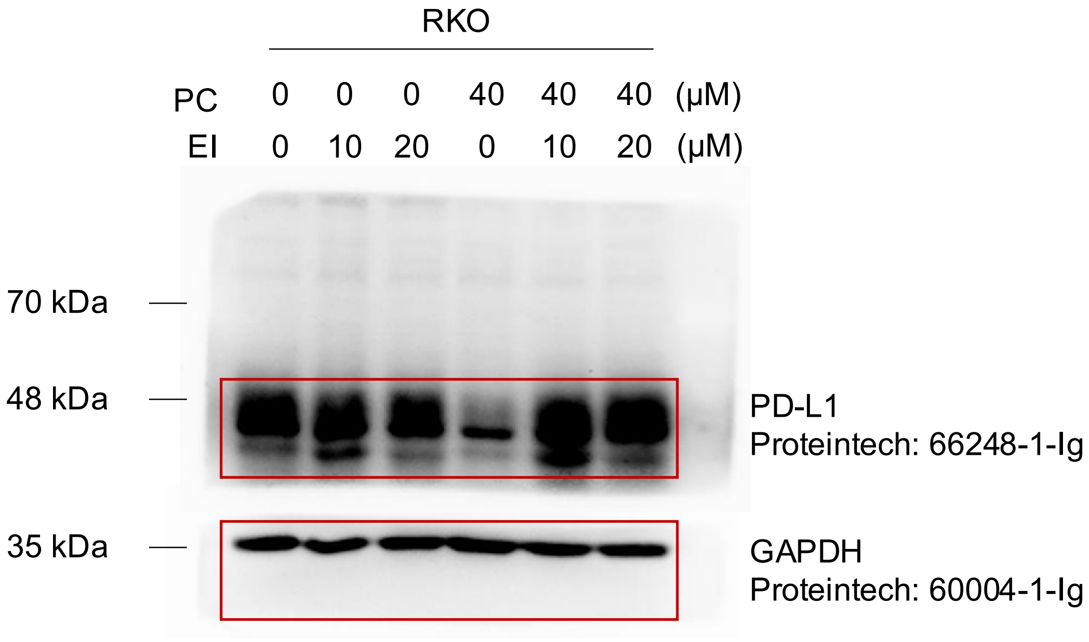

Figure 5F

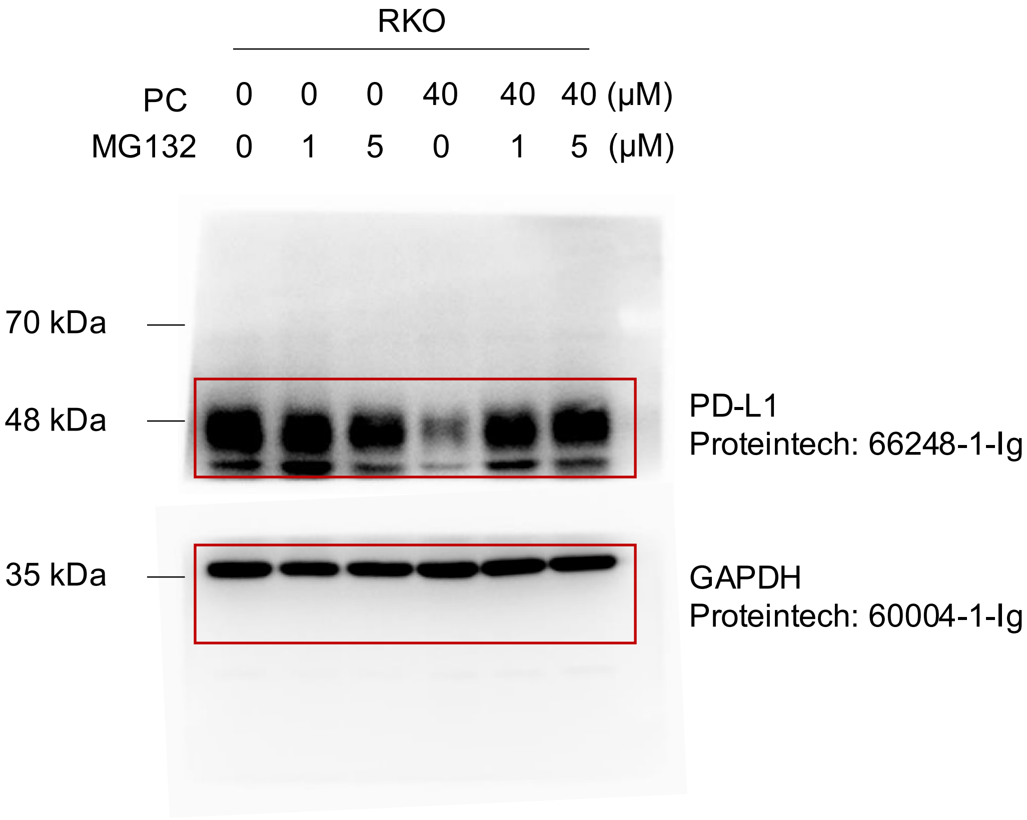

Figure 5G

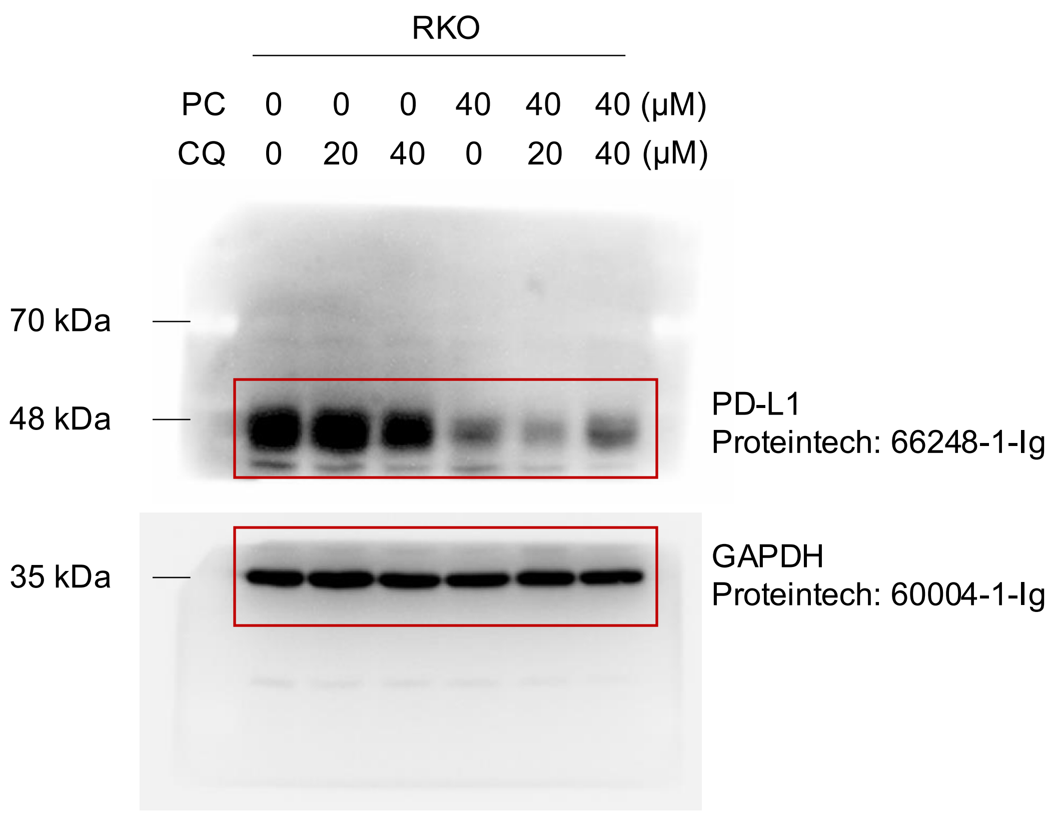

Figure 5H

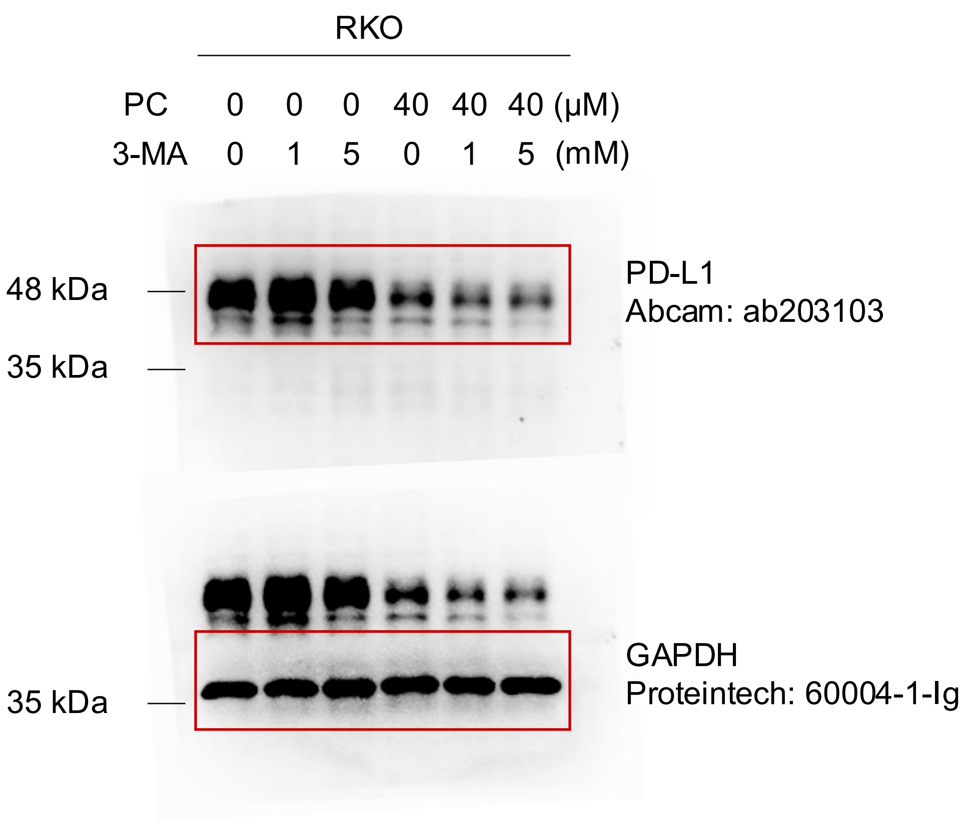

Figure 5O

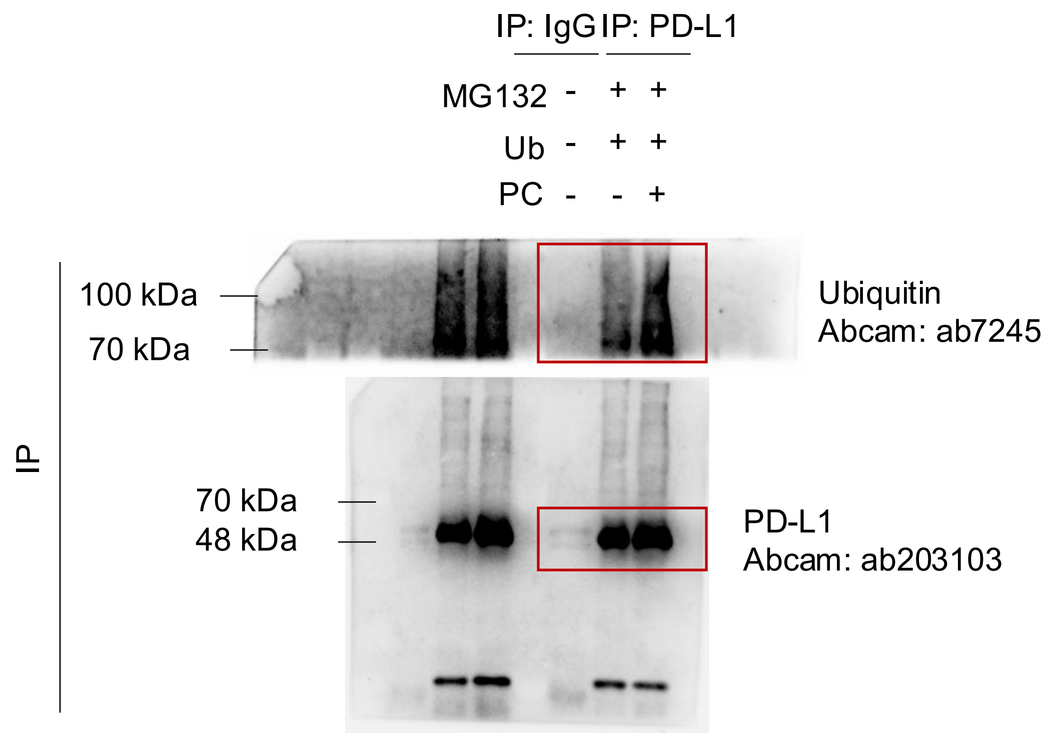
IP: IgG
IP: PD-L1
MG132 - + +
Ub - + +
PC - - +
IP
100 kDa
70 kDa
Ubiquitin
Abcam: ab7245
70 kDa
48 kDa
PD-L1
Abcam: ab203103

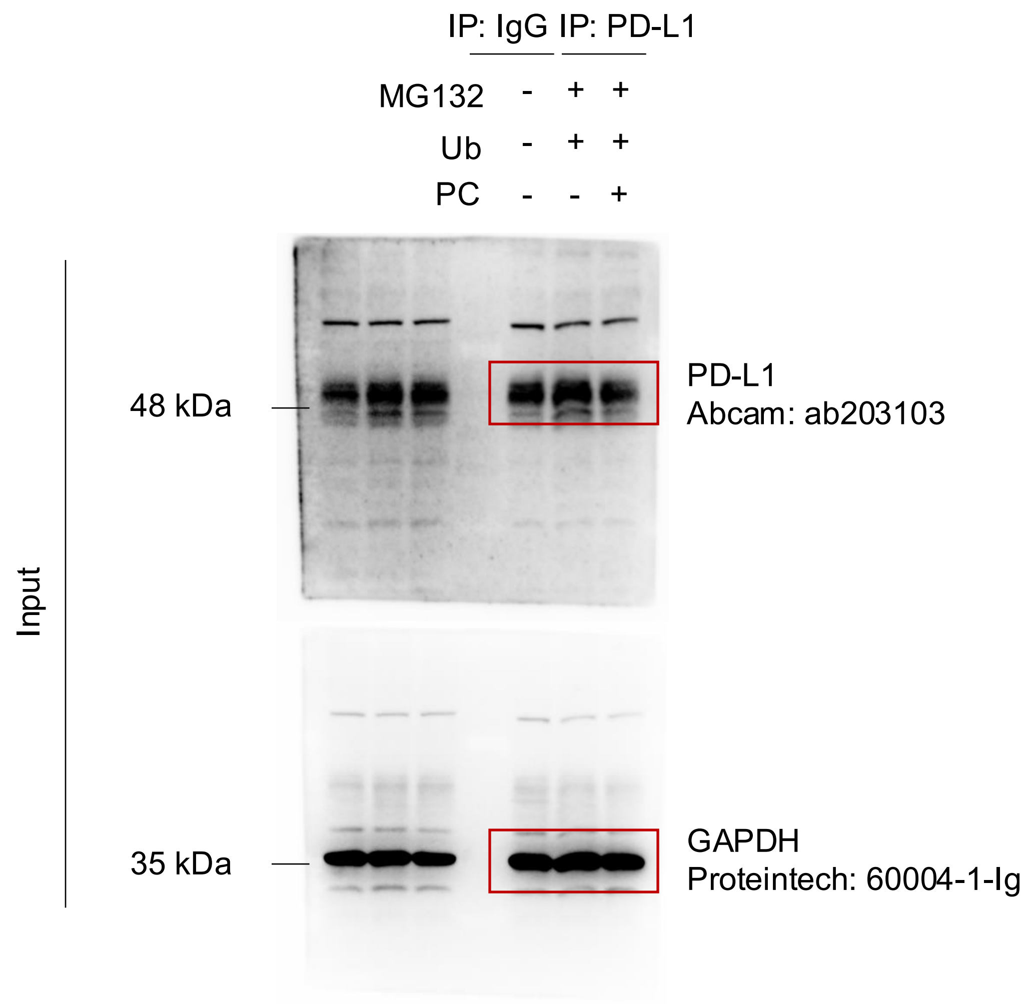
IP: IgG
IP: PD-L1
MG132 - + +
Ub - + +
PC - - +
Input
48 kDa
PD-L1
Abcam: ab203103
35 kDa
GAPDH
Proteintech: 60004-1-Ig

Figure 5P

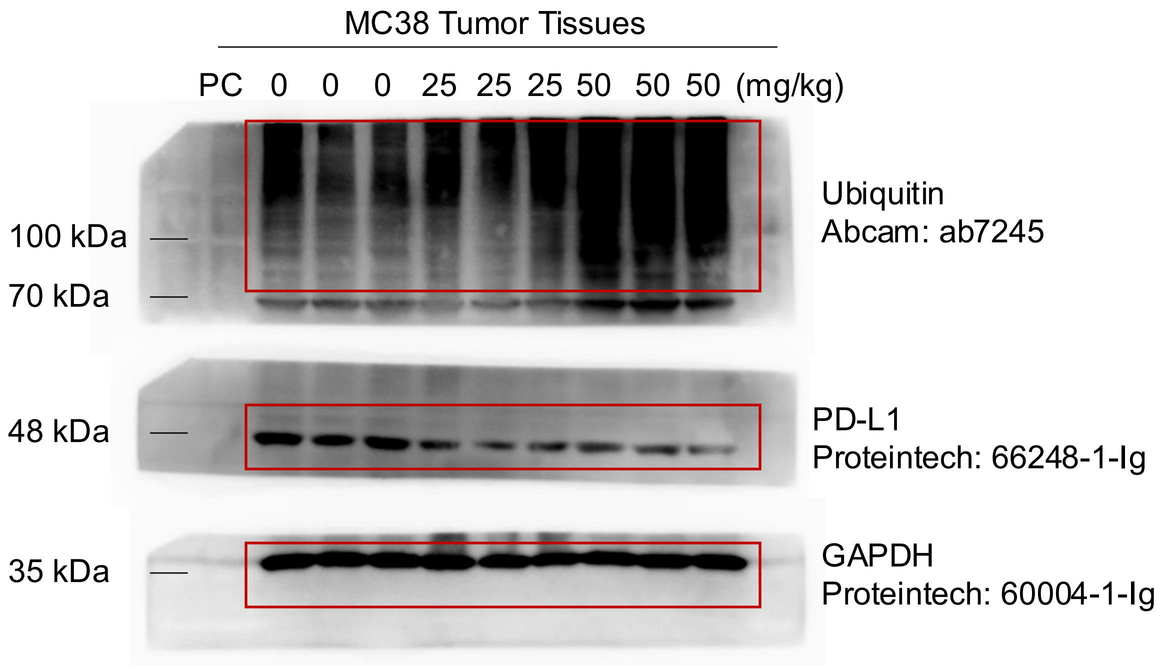

Figure 5Q

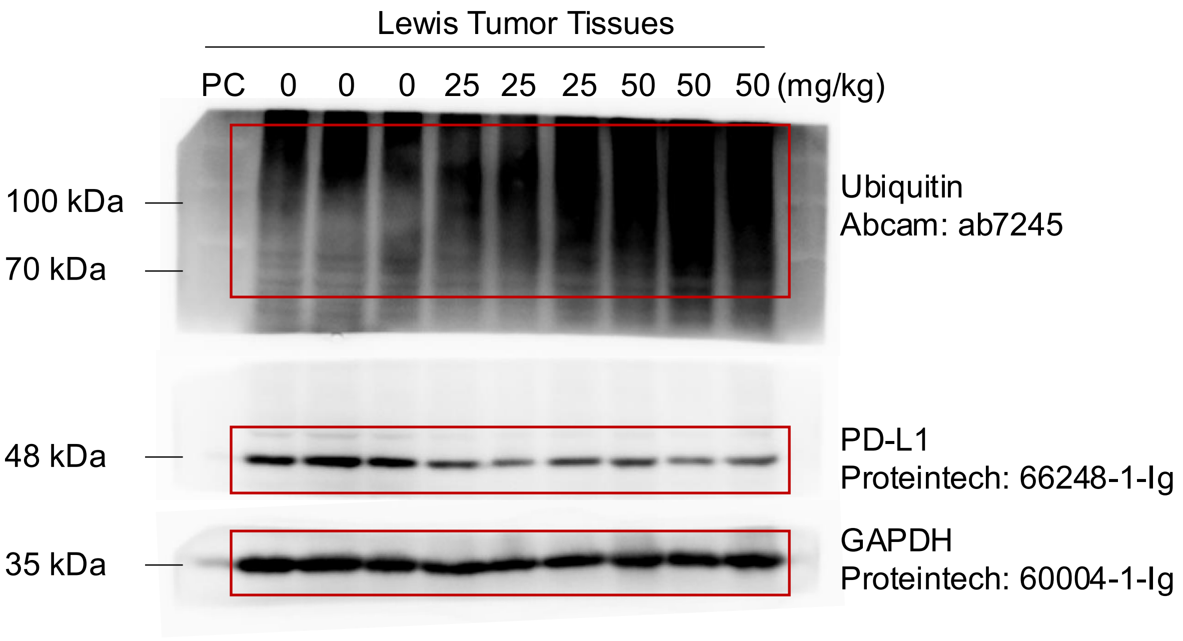

Figure 5R

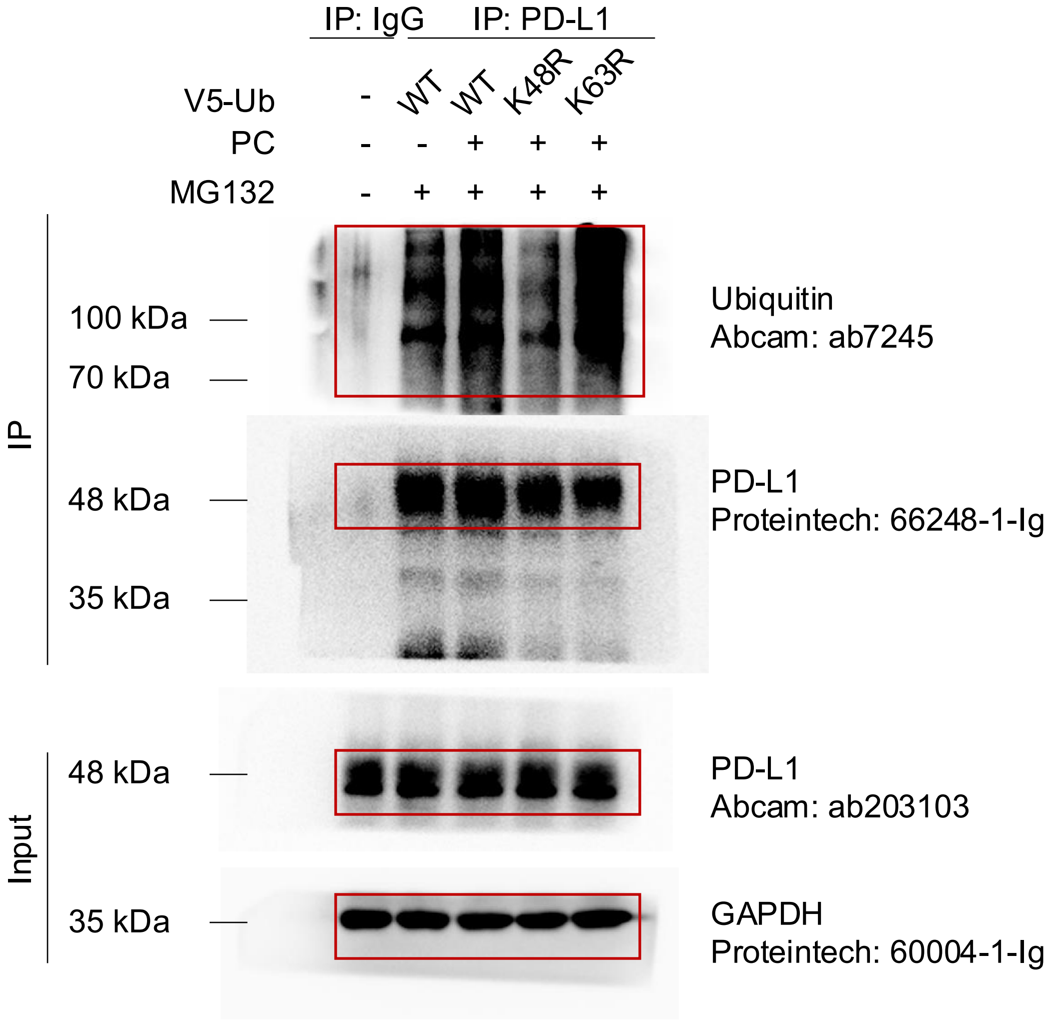

IP: IgG
IP: PD-L1
V5-Ub
-
WT
WT
K48R
K63R
PC
- - + + +
MG132
- + + + +
IP
Input
100 kDa
70 kDa
48 kDa
35 kDa
48 kDa
35 kDa
Ubiquitin
Abcam: ab7245
PD-L1
Proteintech: 66248-1-Ig
PD-L1
Abcam: ab203103
GAPDH
Proteintech: 60004-1-Ig

Figure 6A

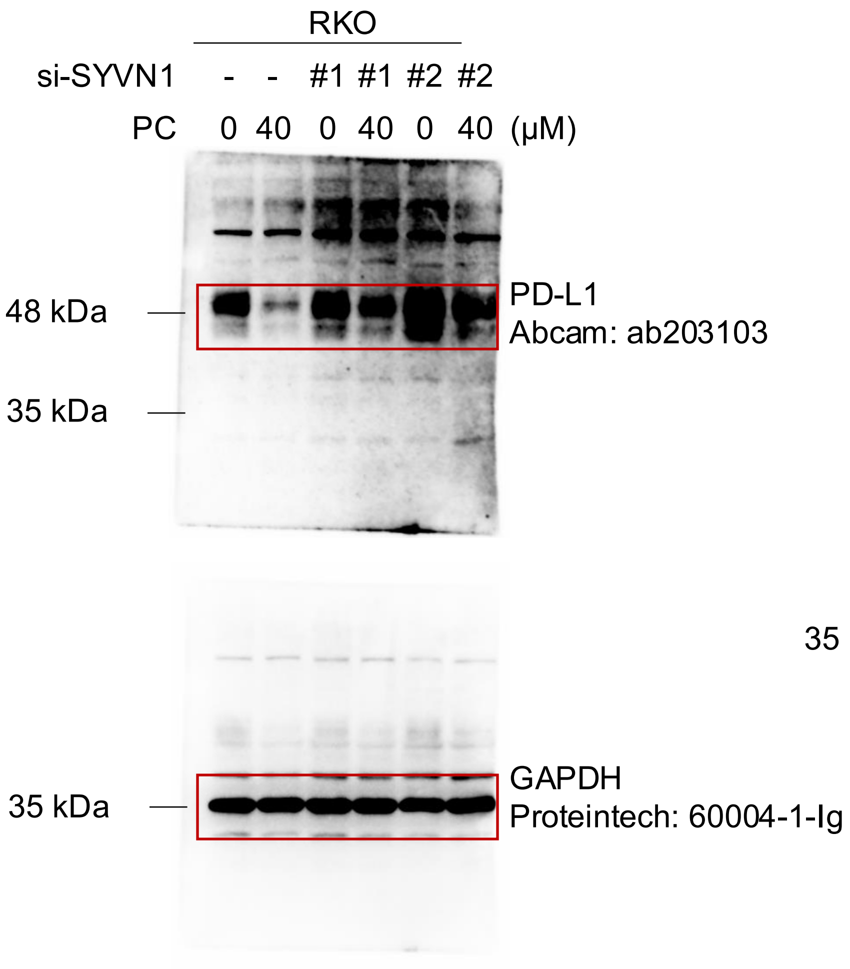

Figure 4D

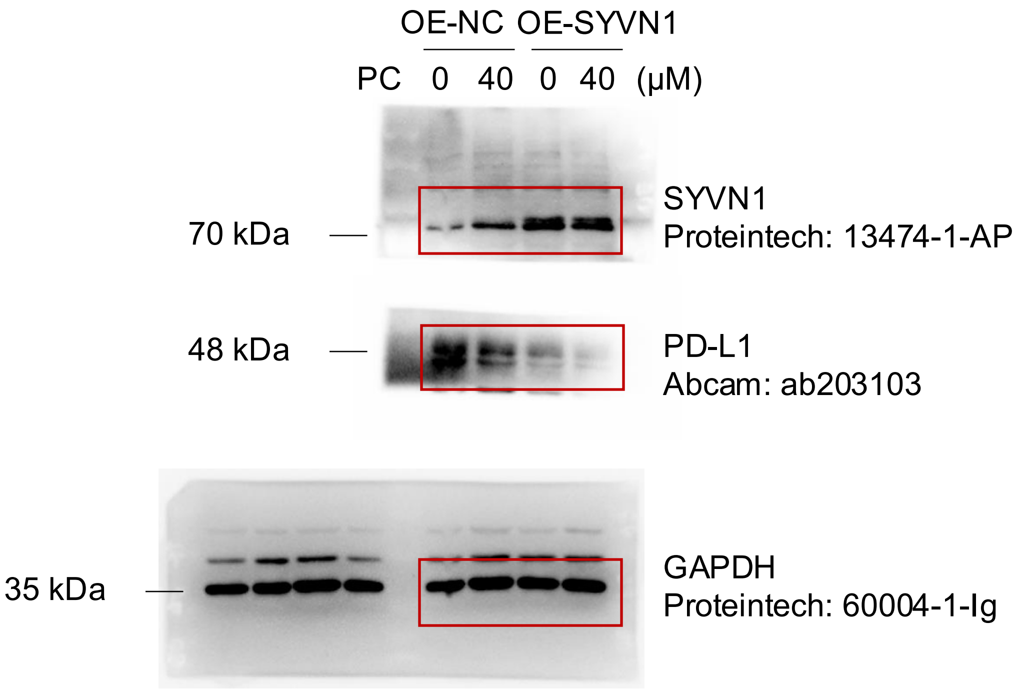

Figure 6K

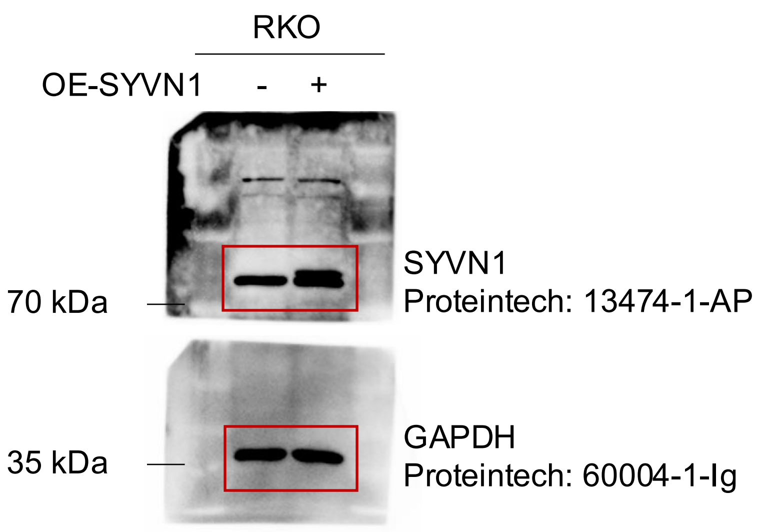
RKO
OE-SYVN1
-
+
70 kDa
SYVN1
Proteintech: 13474-1-AP
35 kDa
GAPDH
Proteintech: 60004-1-Ig

Figure 6O

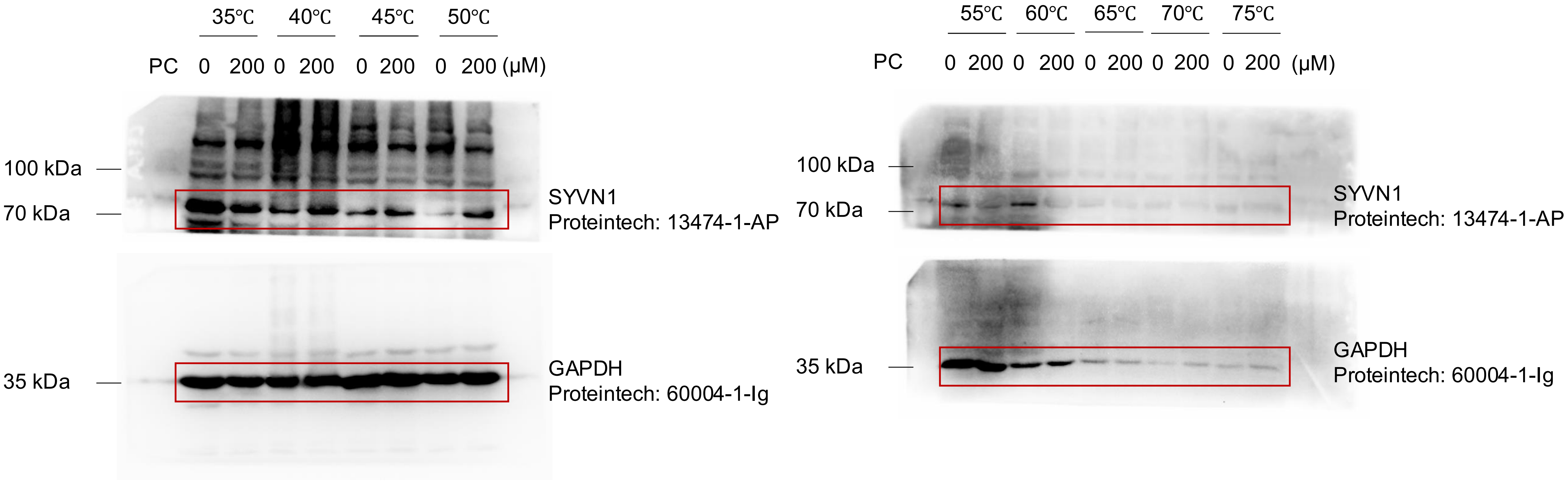
35℃ 40℃ 45℃ 50℃
PC 0 200 0 200 0 200 0 200 (μM)
100 kDa
70 kDa
SYVN1
Proteintech: 13474-1-AP
35 kDa
GAPDH
Proteintech: 60004-1-Ig
55℃ 60℃ 65℃ 70℃ 75℃
PC 0 200 0 200 0 200 0 200 0 200 (μM)
100 kDa
70 kDa
SYVN1
Proteintech: 13474-1-AP
35 kDa
GAPDH
Proteintech: 60004-1-Ig

Figure 6P

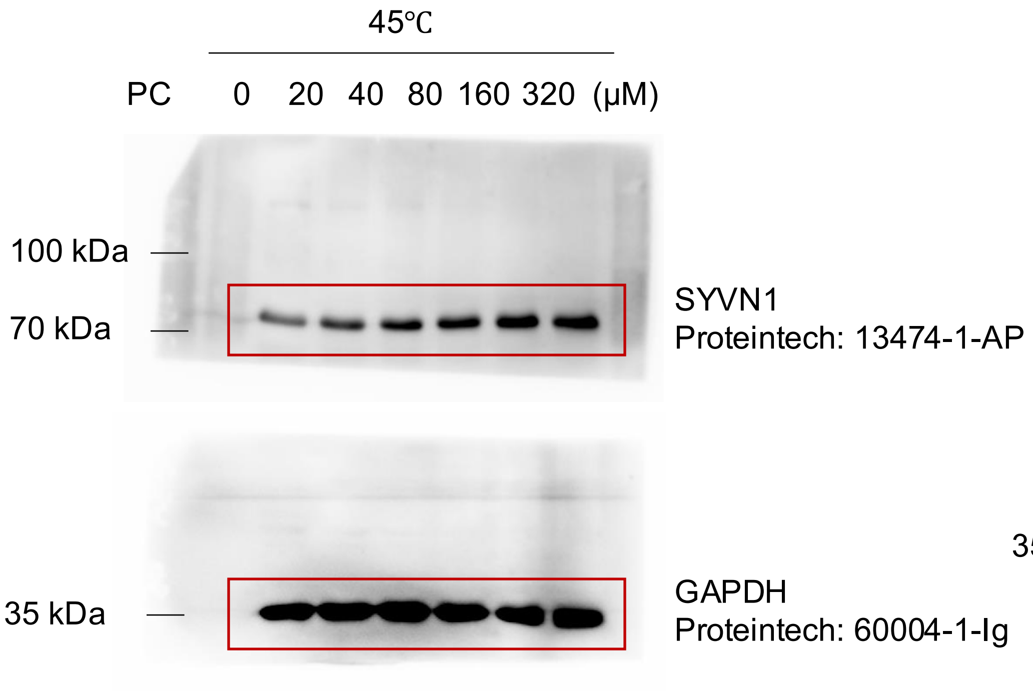

Figure 6Q

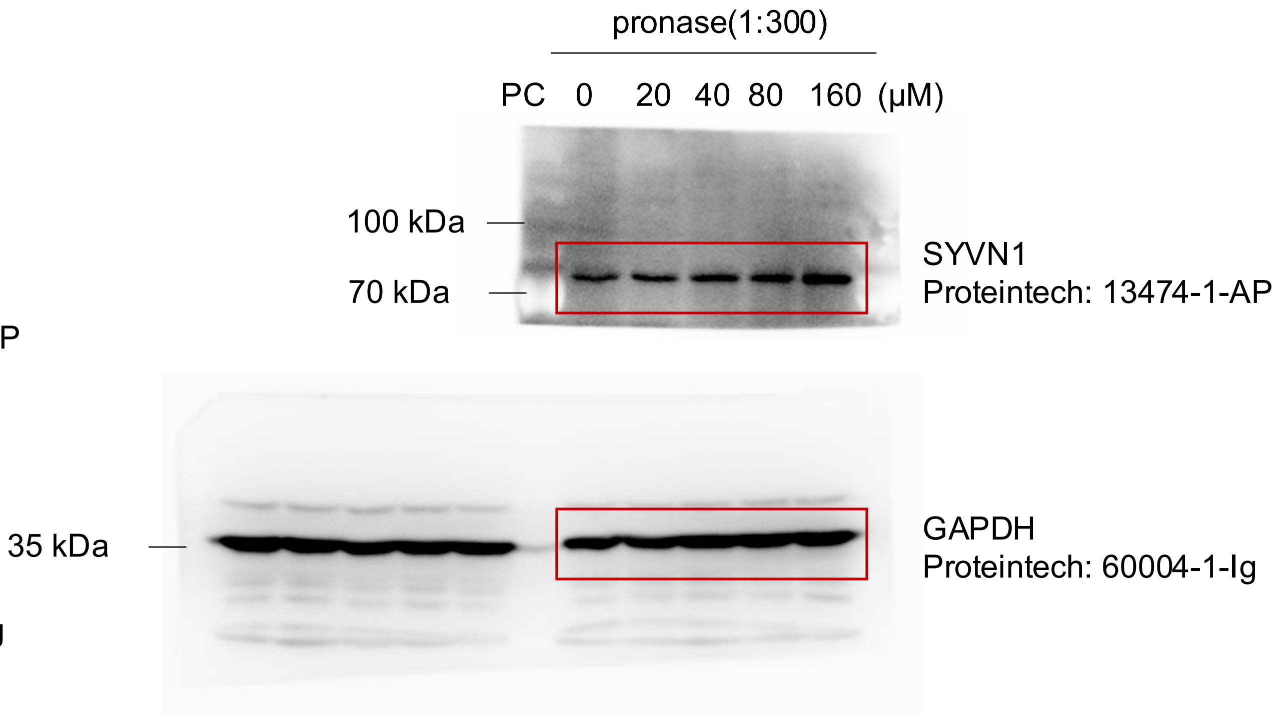

Figure 6X

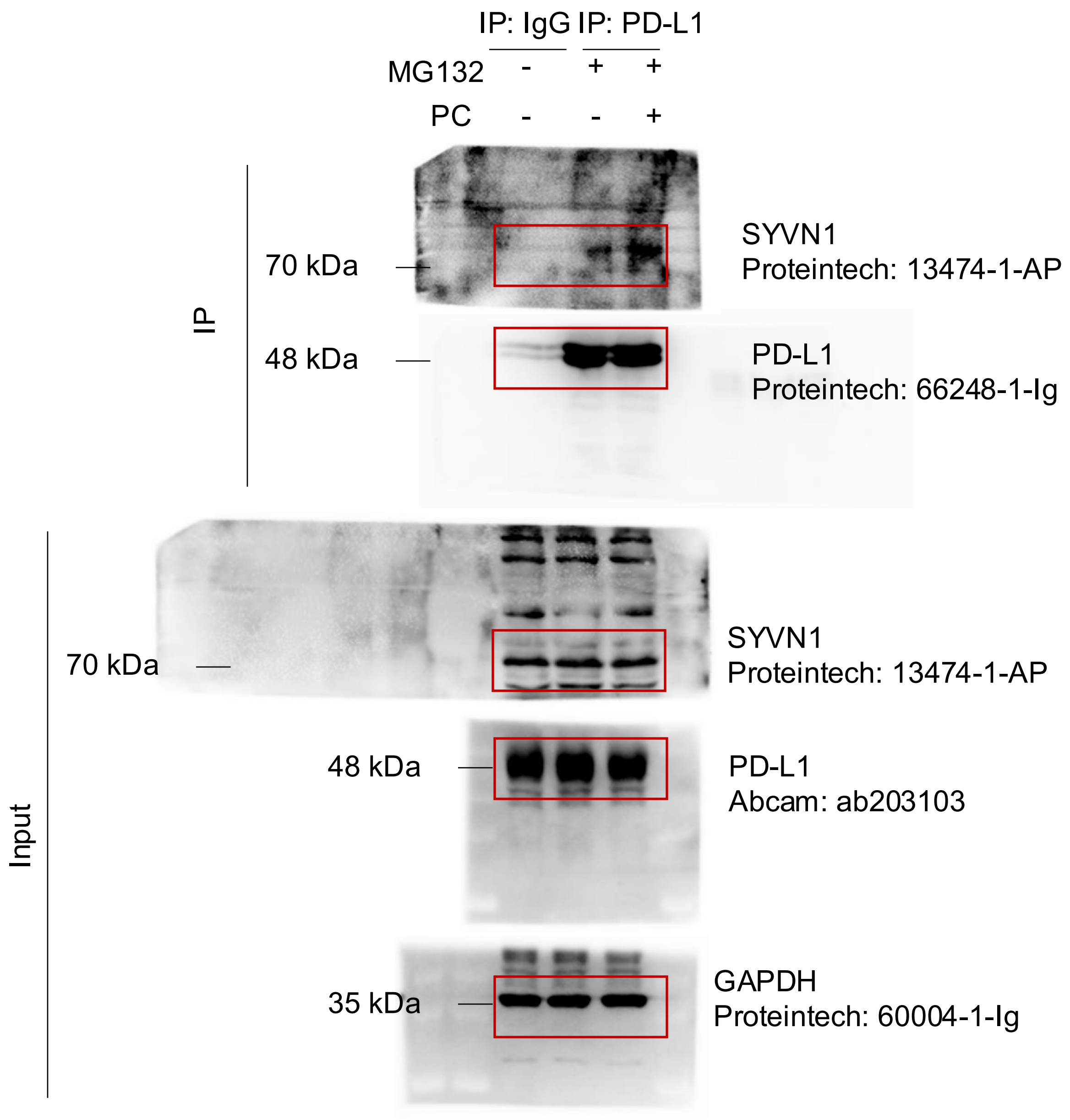

Figure 6Y

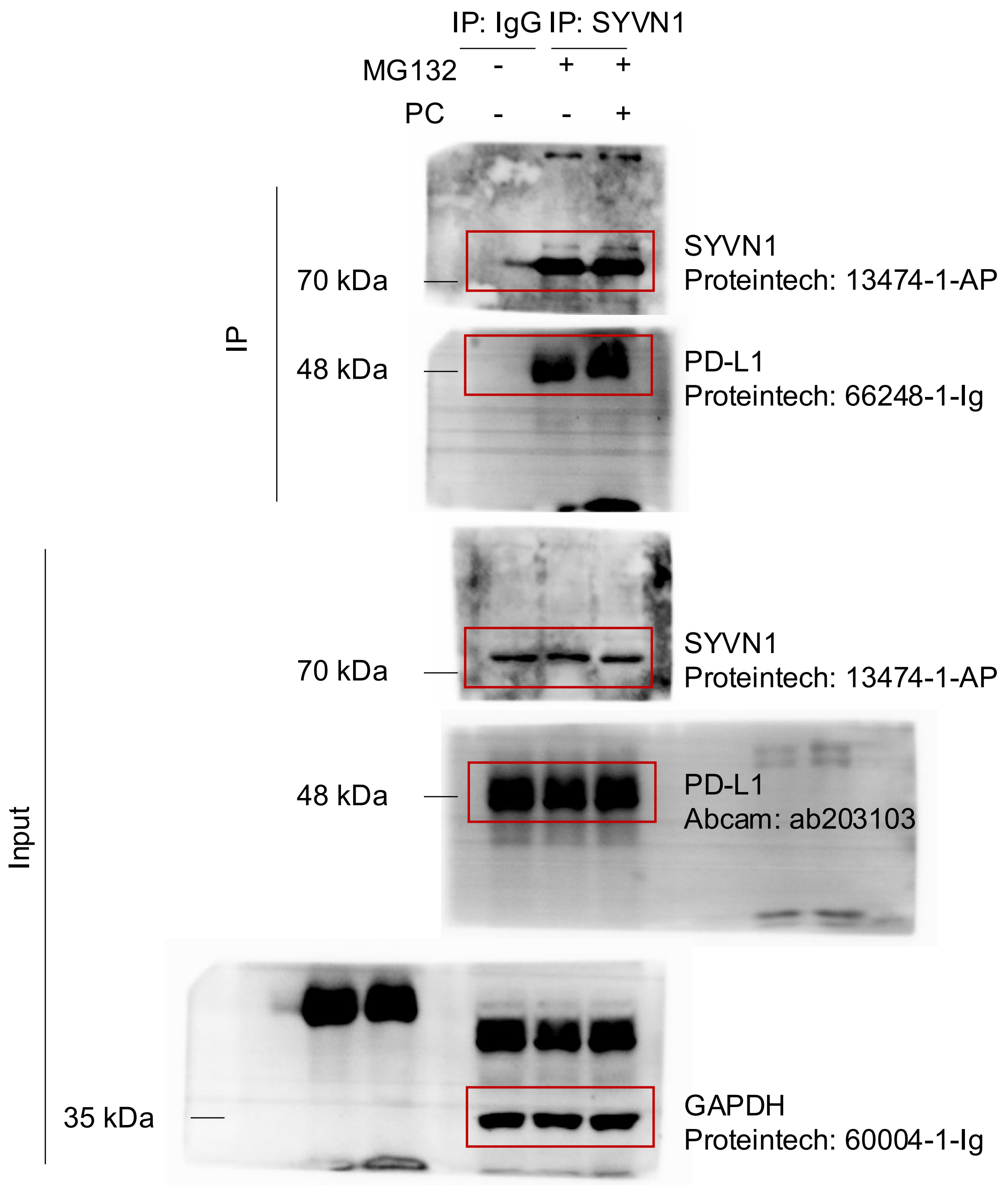

Figure 7E

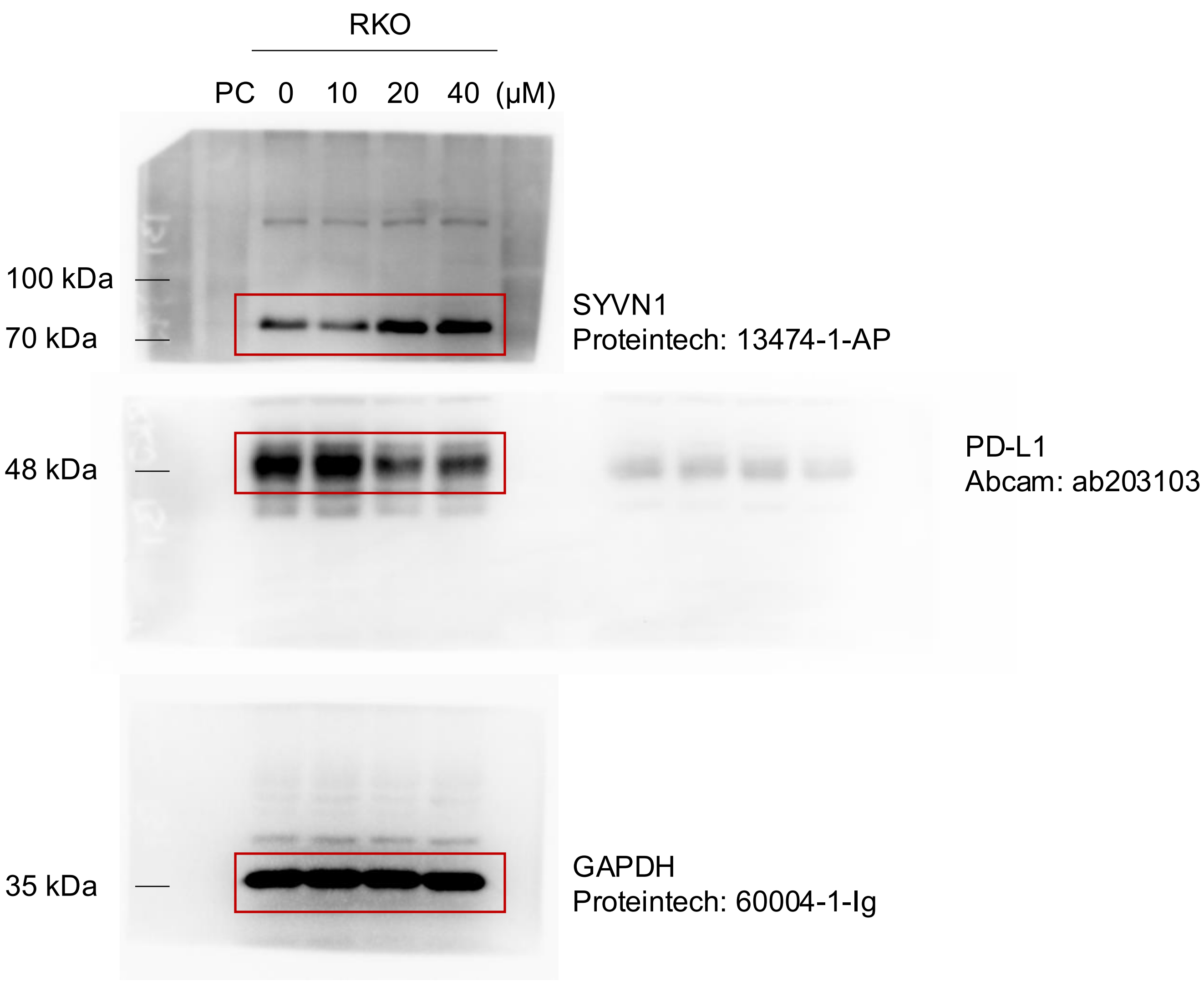

Figure 7F

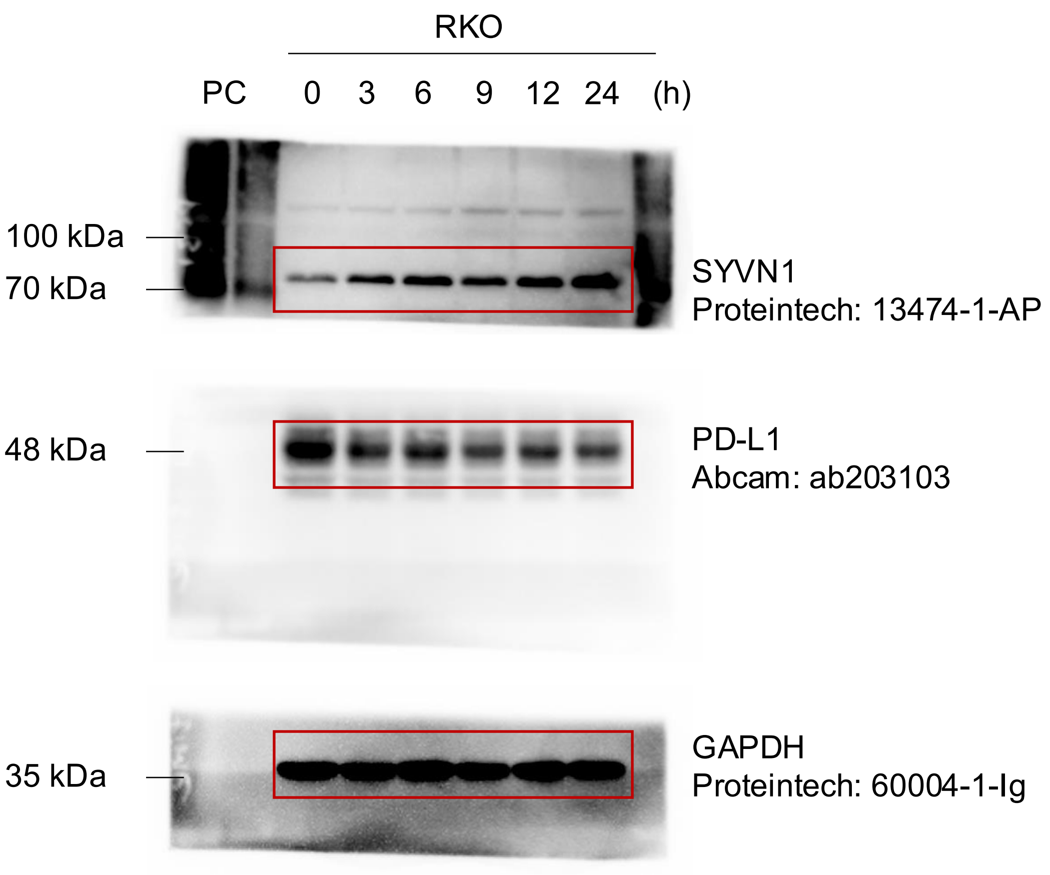

Figure 7H

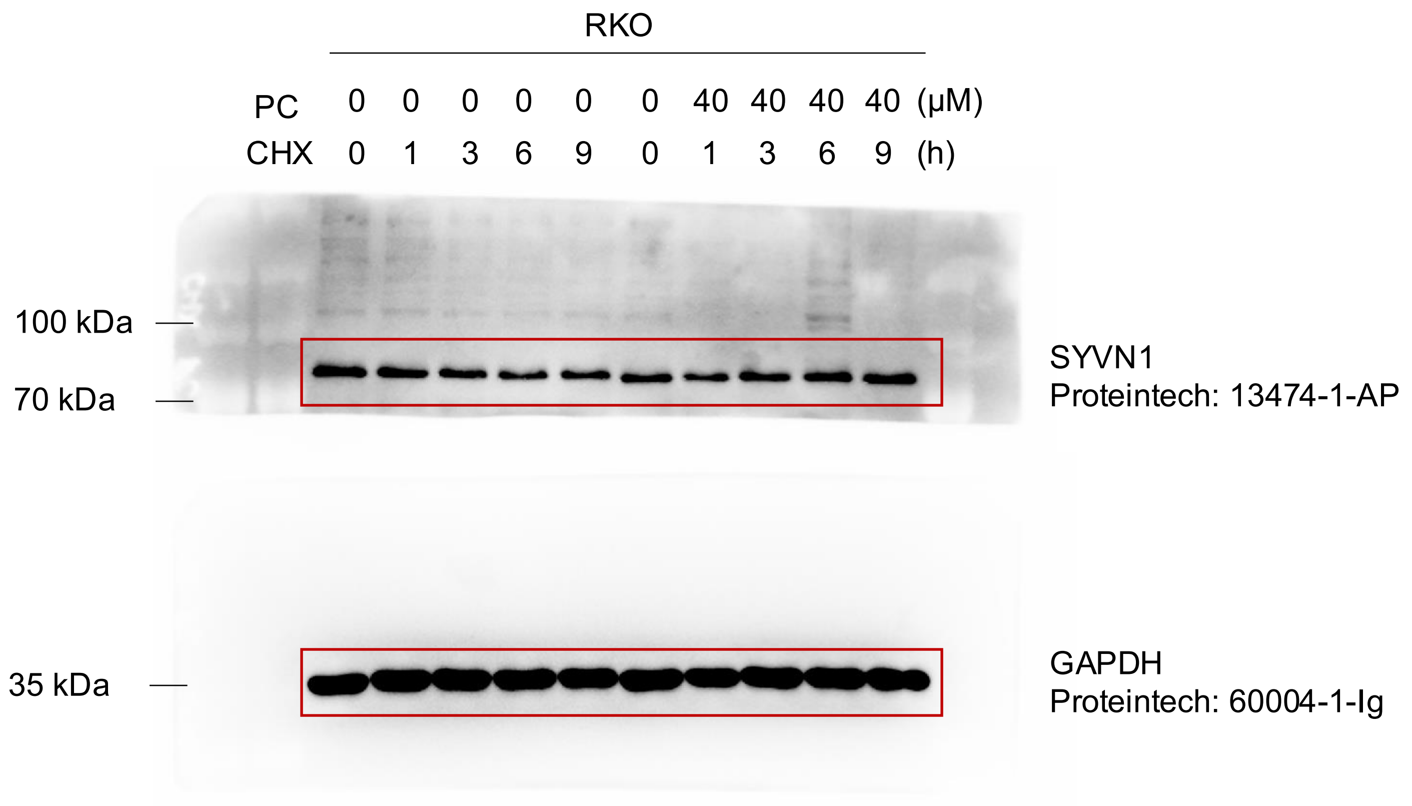
RKO
PC 0 0 0 0 0 0 40 40 40 40 (μM)
CHX 0 1 3 6 9 0 1 3 6 9 (h)
100 kDa
70 kDa
SYVN1
Proteintech: 13474-1-AP
35 kDa
GAPDH
Proteintech: 60004-1-Ig

Figure 7I

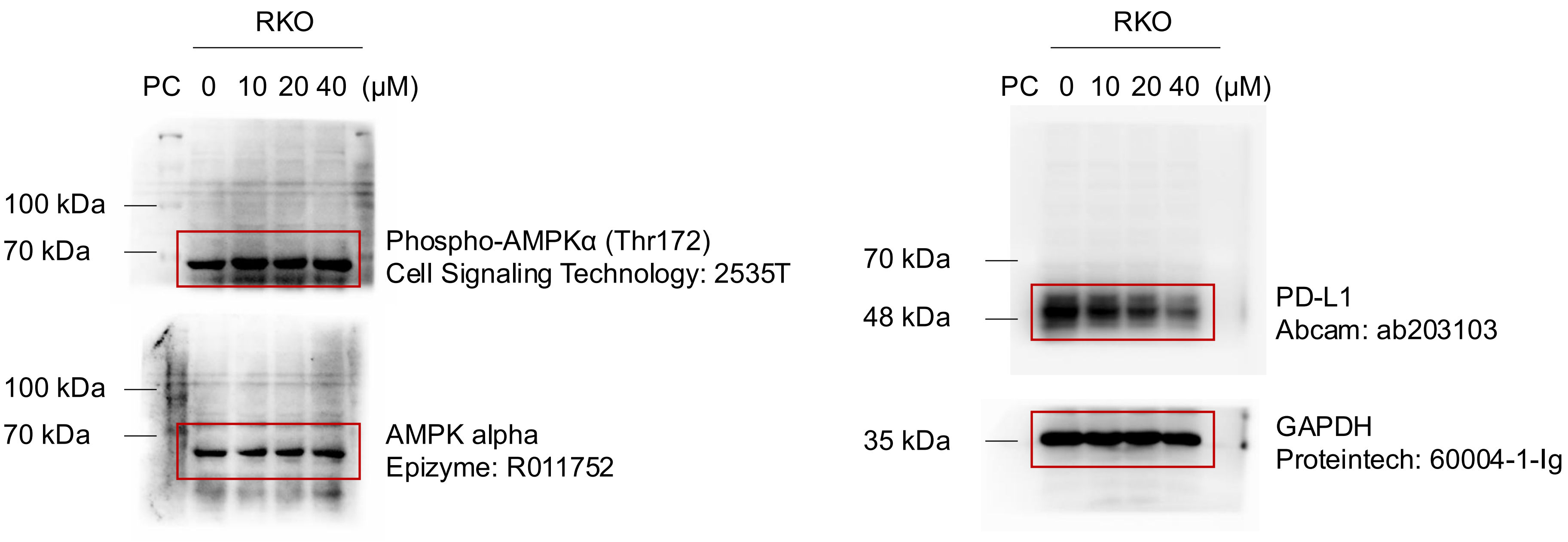
RKO
PC 0 10 20 40 (μM)
100 kDa
70 kDa
Phospho-AMPKα (Thr172)
Cell Signaling Technology: 2535T
100 kDa
70 kDa
AMPK alpha
Epizyme: R011752
RKO
PC 0 10 20 40 (μM)
70 kDa
48 kDa
PD-L1
Abcam: ab203103
35 kDa
GAPDH
Proteintech: 60004-1-Ig

Figure 7K

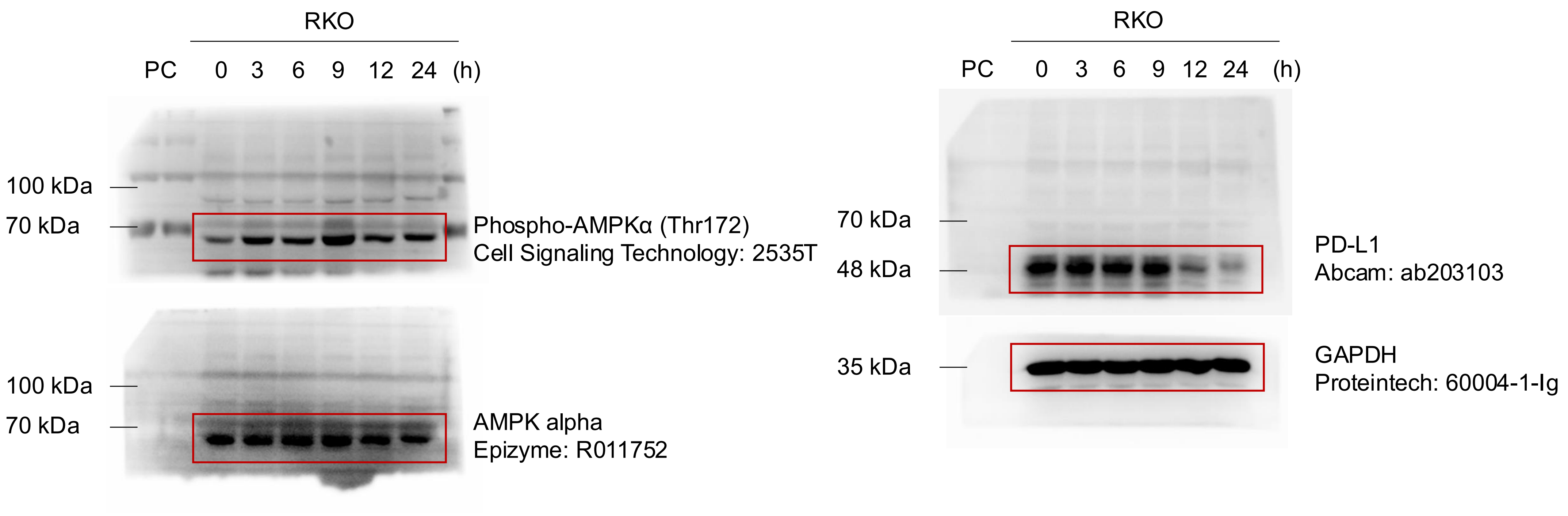
RKO
PC
0
3
6
9
12
24
(h)
100 kDa
70 kDa
Phospho-AMPKα (Thr172)
Cell Signaling Technology: 2535T
100 kDa
70 kDa
AMPK alpha
Epizyme: R011752
RKO
PC
0
3
6
9
12
24
(h)
70 kDa
48 kDa
PD-L1
Abcam: ab203103
35 kDa
GAPDH
Proteintech: 60004-1-Ig

Figure 7O

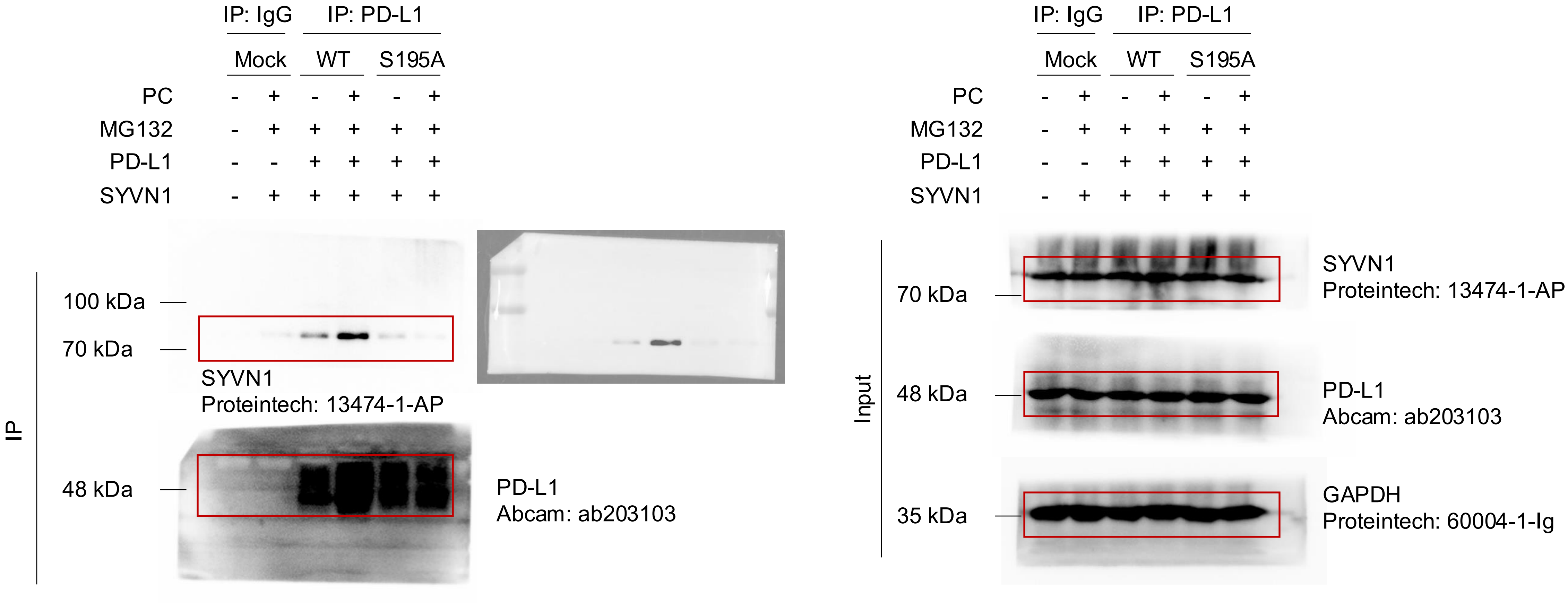
IP: IgG
IP: PD-L1
Mock
WT
S195A
PC
MG132
PD-L1
SYVN1
IP
100 kDa
70 kDa
SYVN1
Proteintech: 13474-1-AP
48 kDa
PD-L1
Abcam: ab203103
IP: IgG
IP: PD-L1
Mock
WT
S195A
PC
MG132
PD-L1
SYVN1
Input
70 kDa
SYVN1
Proteintech: 13474-1-AP
48 kDa
PD-L1
Abcam: ab203103
35 kDa
GAPDH
Proteintech: 60004-1-Ig

Figure 7P

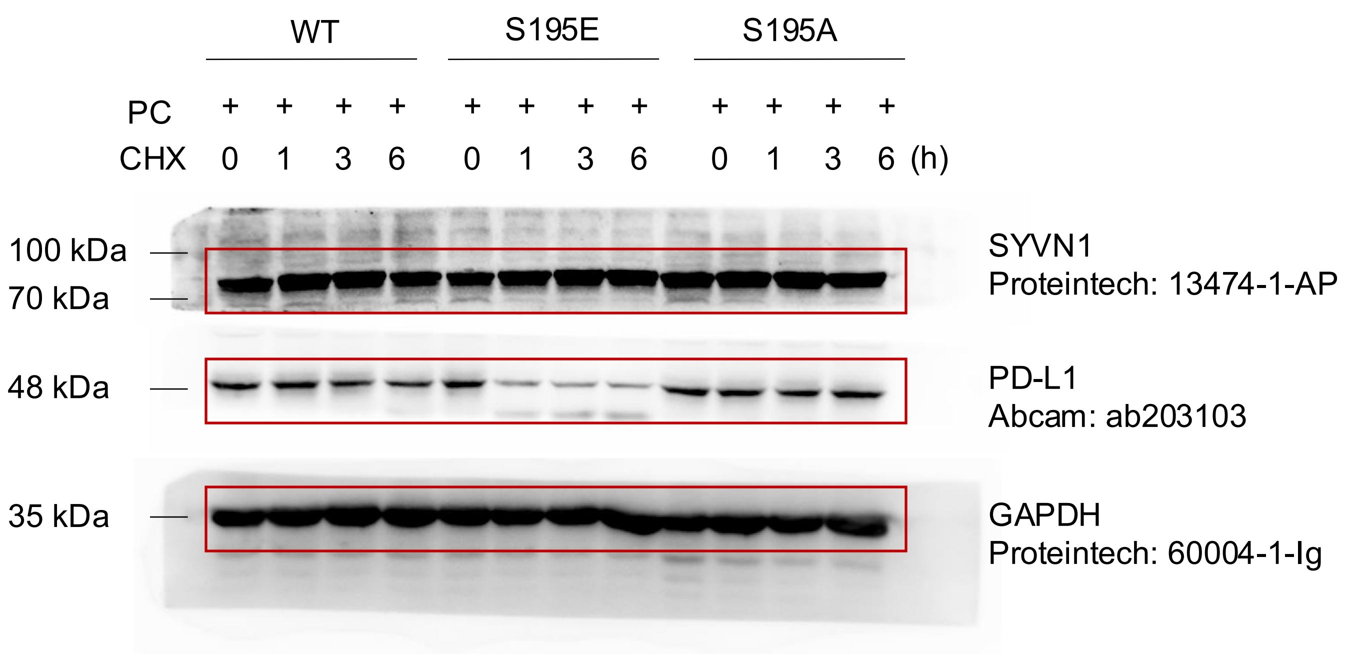
WT
S195E
S195A
PC + + + + + + + + + + + +
CHX 0 1 3 6 0 1 3 6 0 1 3 6 (h)
100 kDa
70 kDa
48 kDa
35 kDa
SYVN1
Proteintech: 13474-1-AP
PD-L1
Abcam: ab203103
GAPDH
Proteintech: 60004-1-Ig

Figure 8A

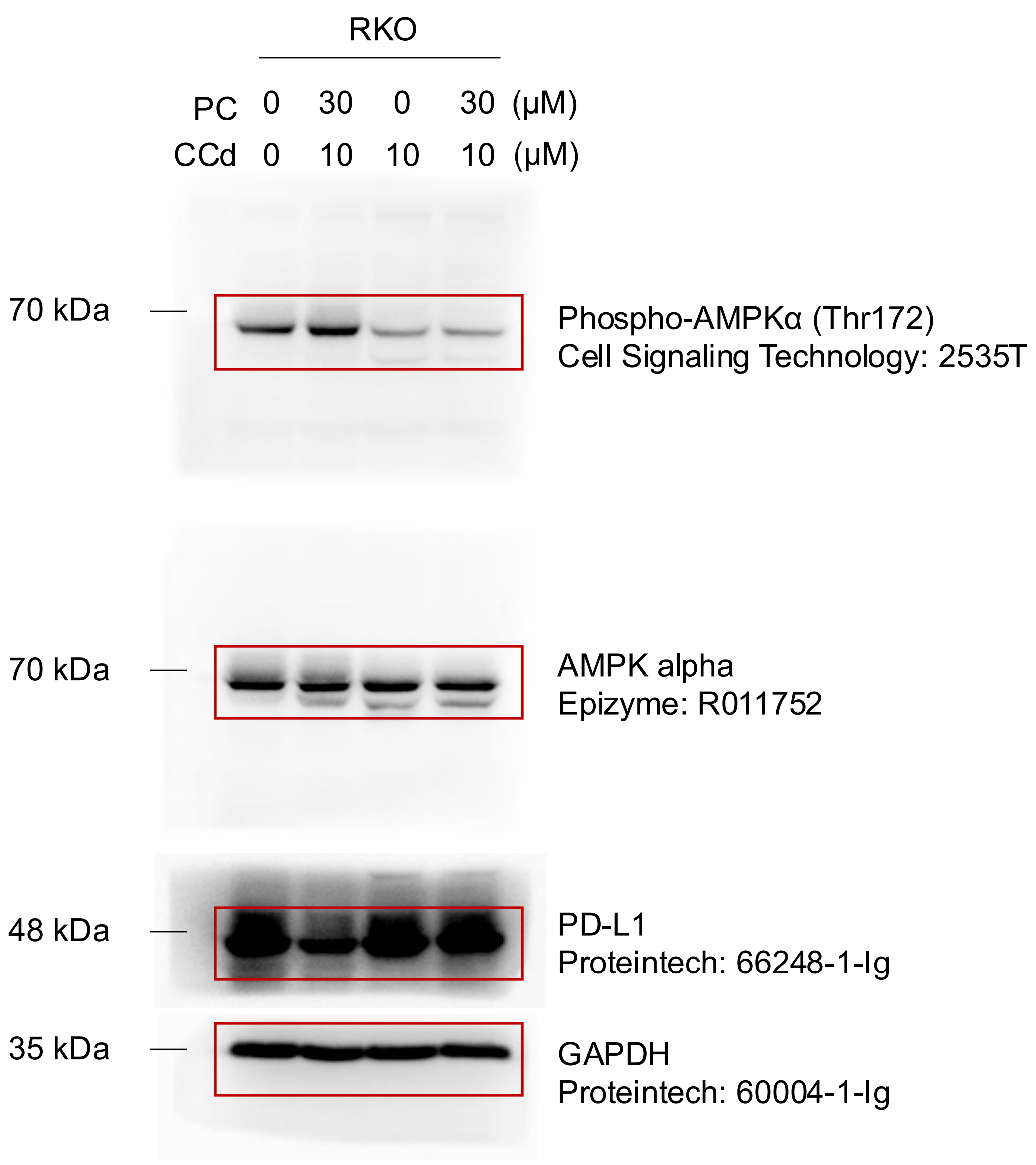

RKO
PC 0 30 0 30 (μM)
CCd 0 10 10 10 (μM)
70 kDa
Phospho-AMPKα (Thr172)
Cell Signaling Technology: 2535T
70 kDa
AMPK alpha
Epizyme: R011752
48 kDa
PD-L1
Proteintech: 66248-1-Ig
35 kDa
GAPDH
Proteintech: 60004-1-Ig

Figure 8B

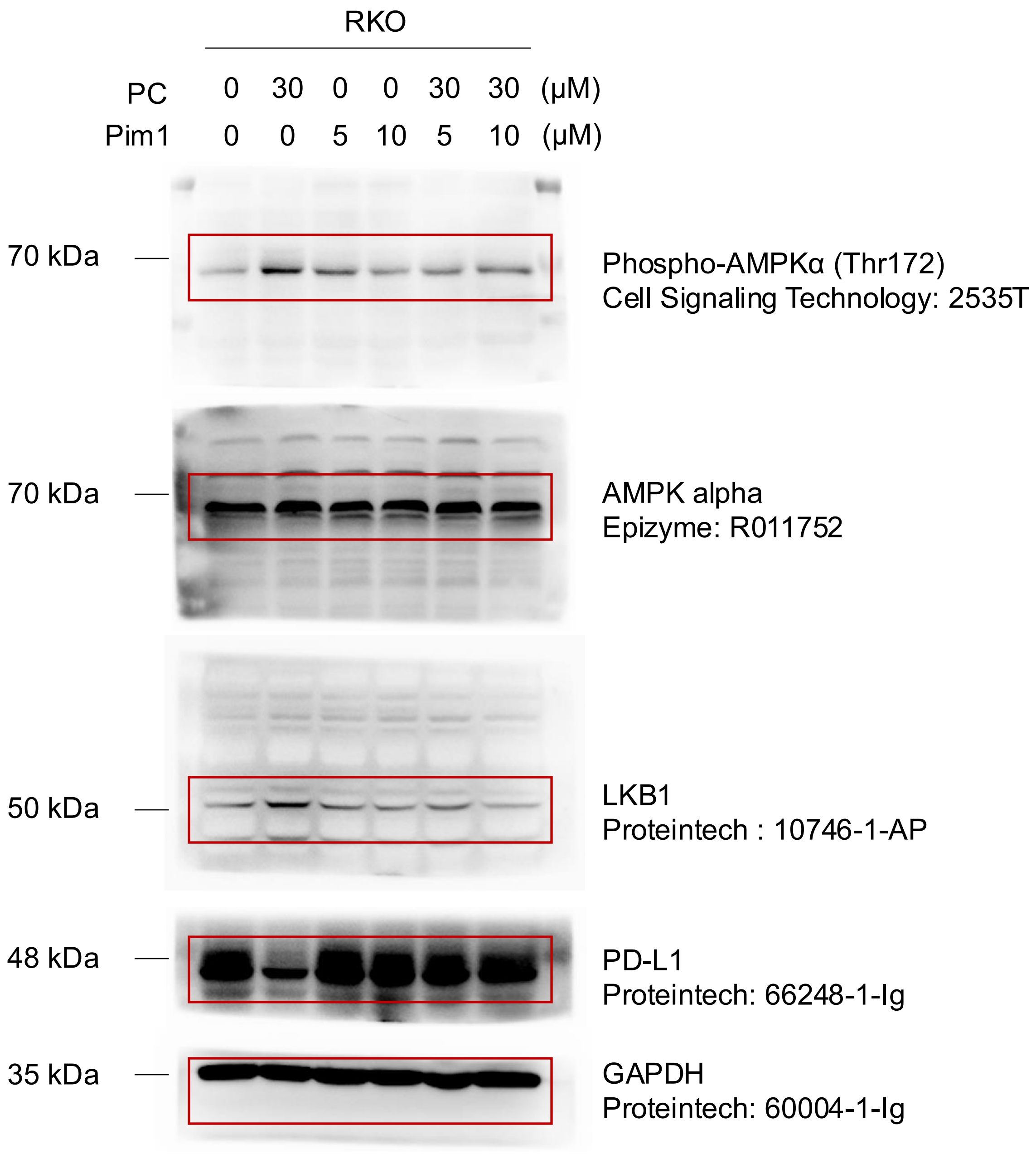
RKO
PC 0 30 0 0 30 30 (μM)
Pim1 0 0 5 10 5 10 (μM)
70 kDa
Phospho-AMPKα (Thr172)
Cell Signaling Technology: 2535T
70 kDa
AMPK alpha
Epizyme: R011752
50 kDa
LKB1
Proteintech : 10746-1-AP
48 kDa
PD-L1
Proteintech: 66248-1-Ig
35 kDa
GAPDH
Proteintech: 60004-1-Ig

Figure 8C

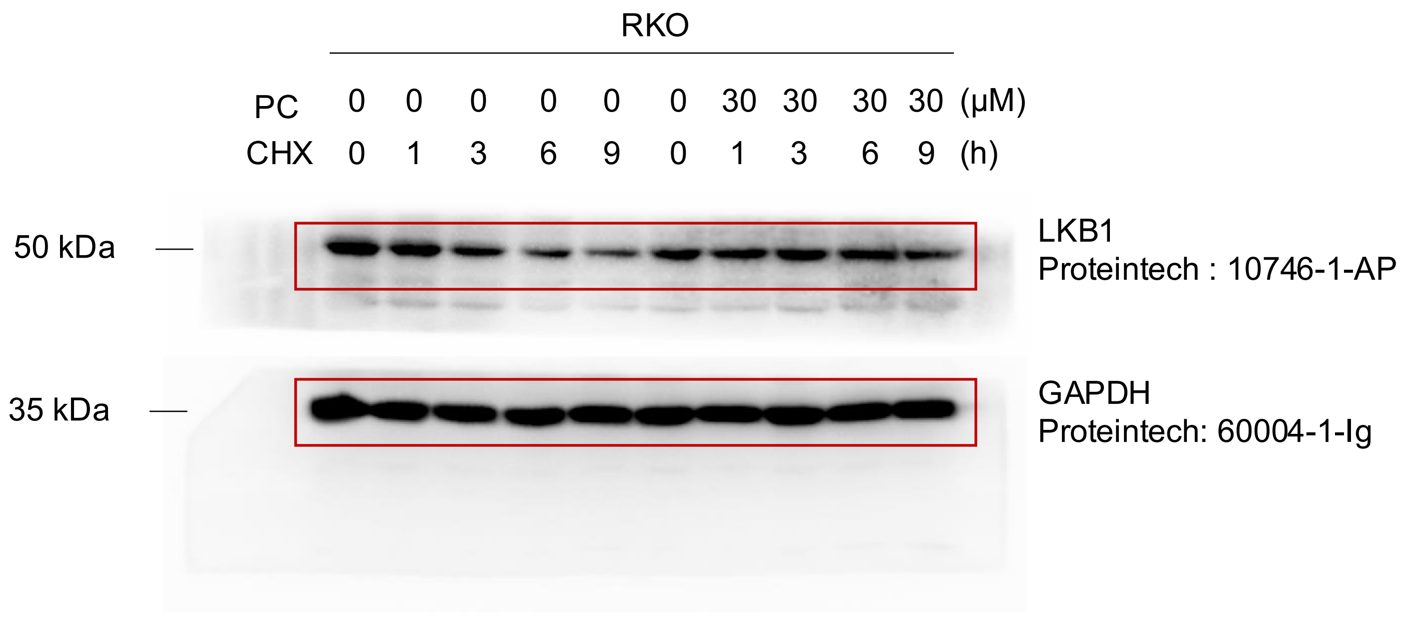
RKO
PC 0 0 0 0 0 0 30 30 30 30 (μM)
CHX 0 1 3 6 9 0 1 3 6 9 (h)
50 kDa
LKB1
Proteintech : 10746-1-AP
35 kDa
GAPDH
Proteintech: 60004-1-Ig

Figure 8E

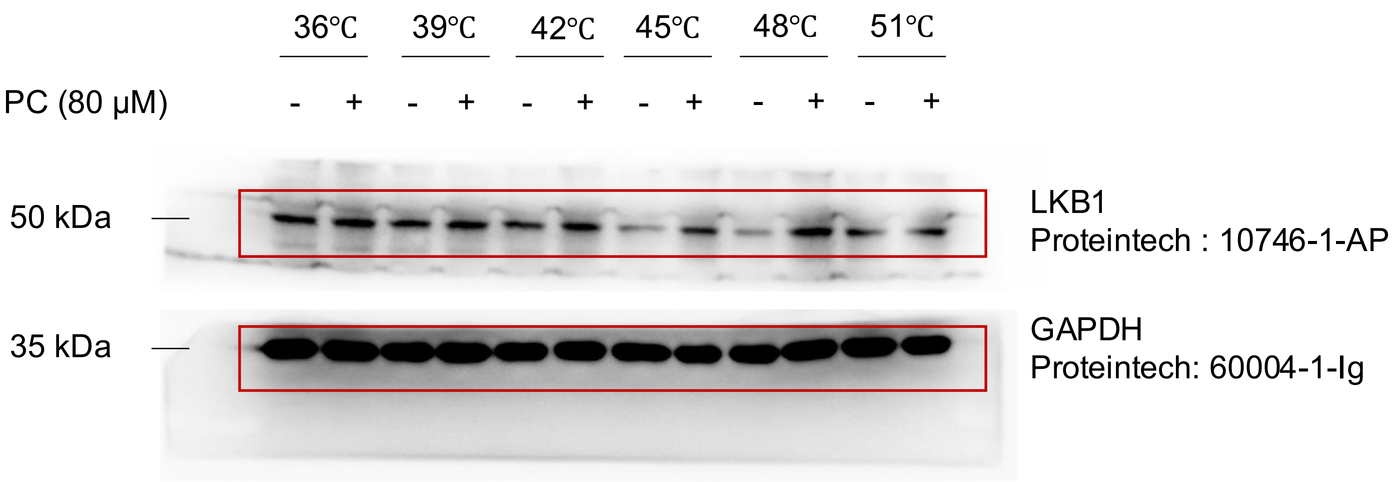

36℃
39℃
42℃
45℃
48℃
51℃
PC (80 μM)
- + - + - + - + - + - +
50 kDa
35 kDa
LKB1
Proteintech : 10746-1-AP
GAPDH
Proteintech: 60004-1-Ig

Figure 8F

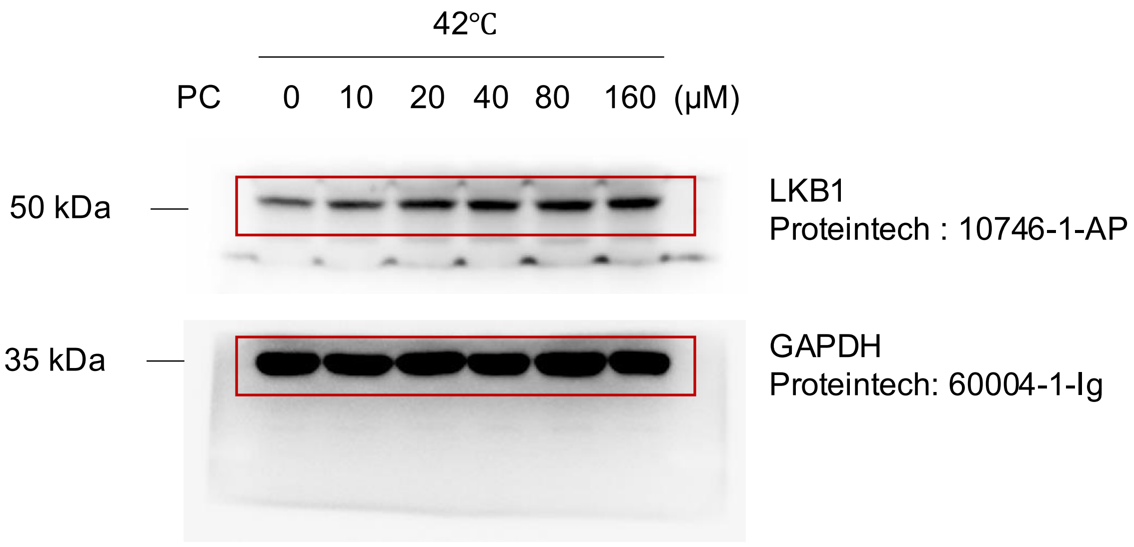

Figure 8H

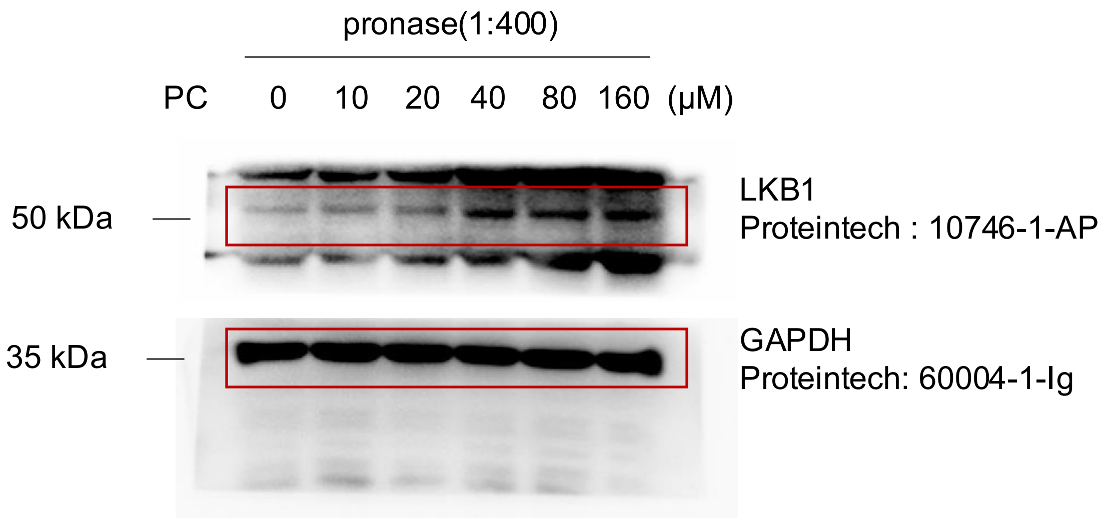

Figure S2L

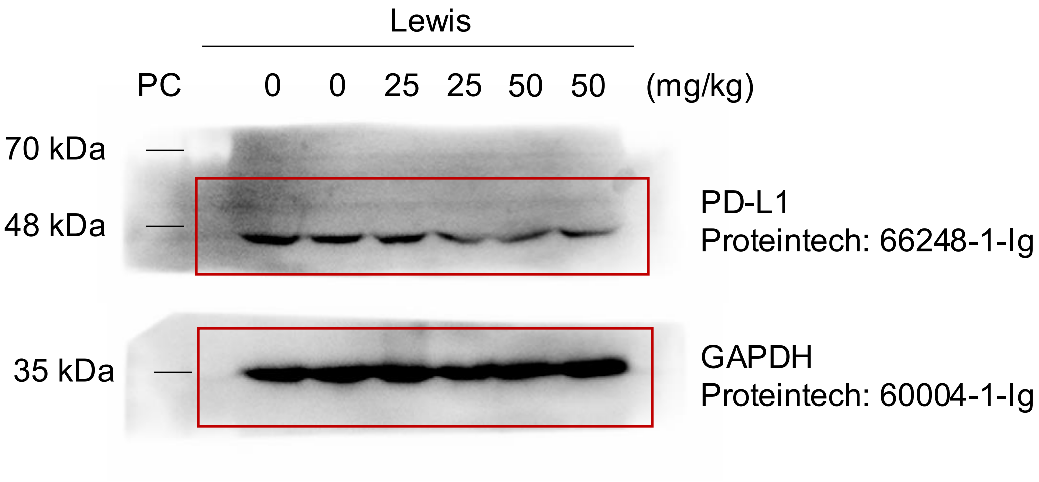
Lewis
PC
0
0
25
25
50
50
(mg/kg)
70 kDa
48 kDa
PD-L1
Proteintech: 66248-1-Ig
35 kDa
GAPDH
Proteintech: 60004-1-Ig

Figure S8B

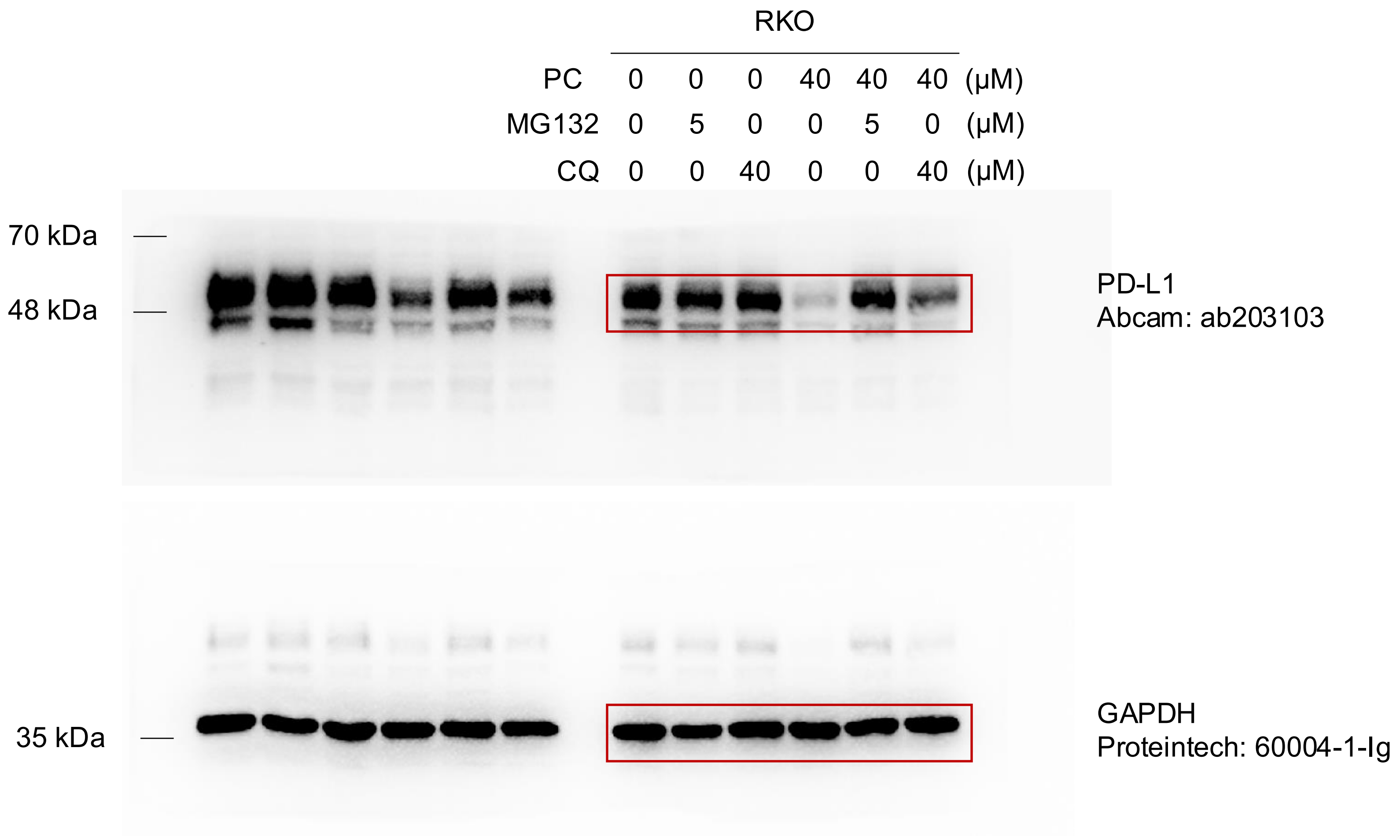

RKO
PC 0 0 0 40 40 40 (μM)
MG132 0 5 0 0 5 0 (μM)
CQ 0 0 40 0 0 40 (μM)
70 kDa
48 kDa
PD-L1
Abcam: ab203103
35 kDa
GAPDH
Proteintech: 60004-1-Ig

Figure S7C

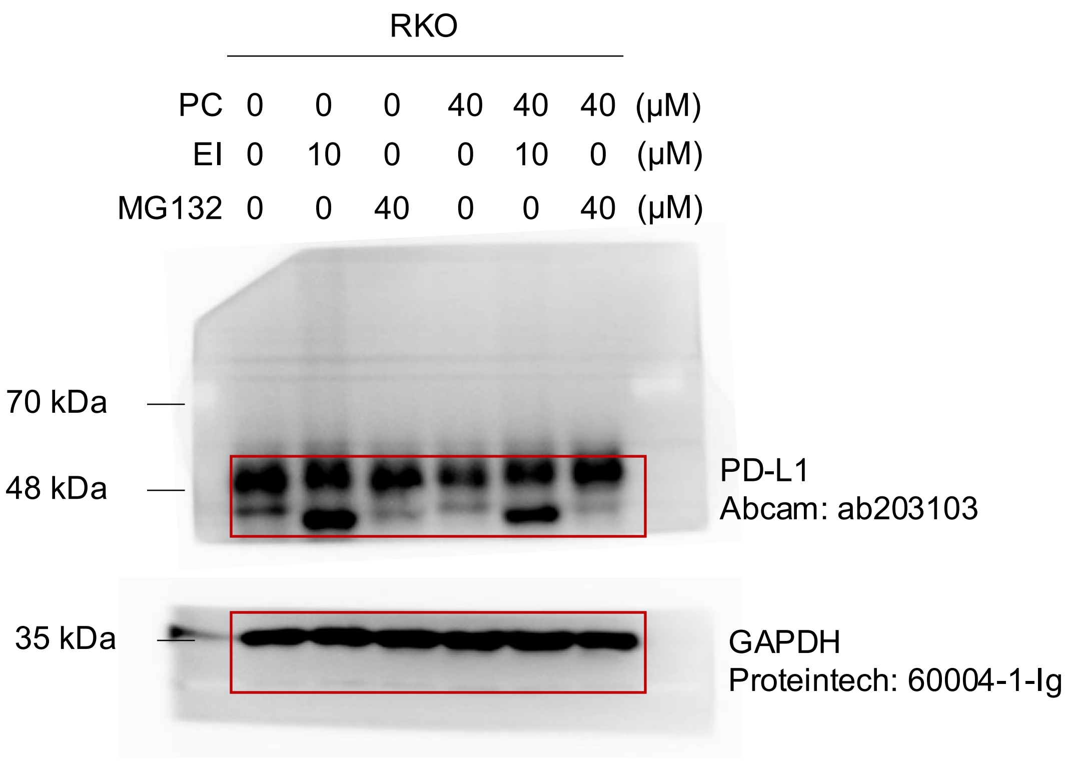
RKO
PC 0 0 0 40 40 40 (μM)
EI 0 10 0 0 10 0 (μM)
MG132 0 0 40 0 0 40 (μM)
70 kDa
48 kDa
PD-L1
Abcam: ab203103
35 kDa
GAPDH
Proteintech: 60004-1-Ig

Figure S9A

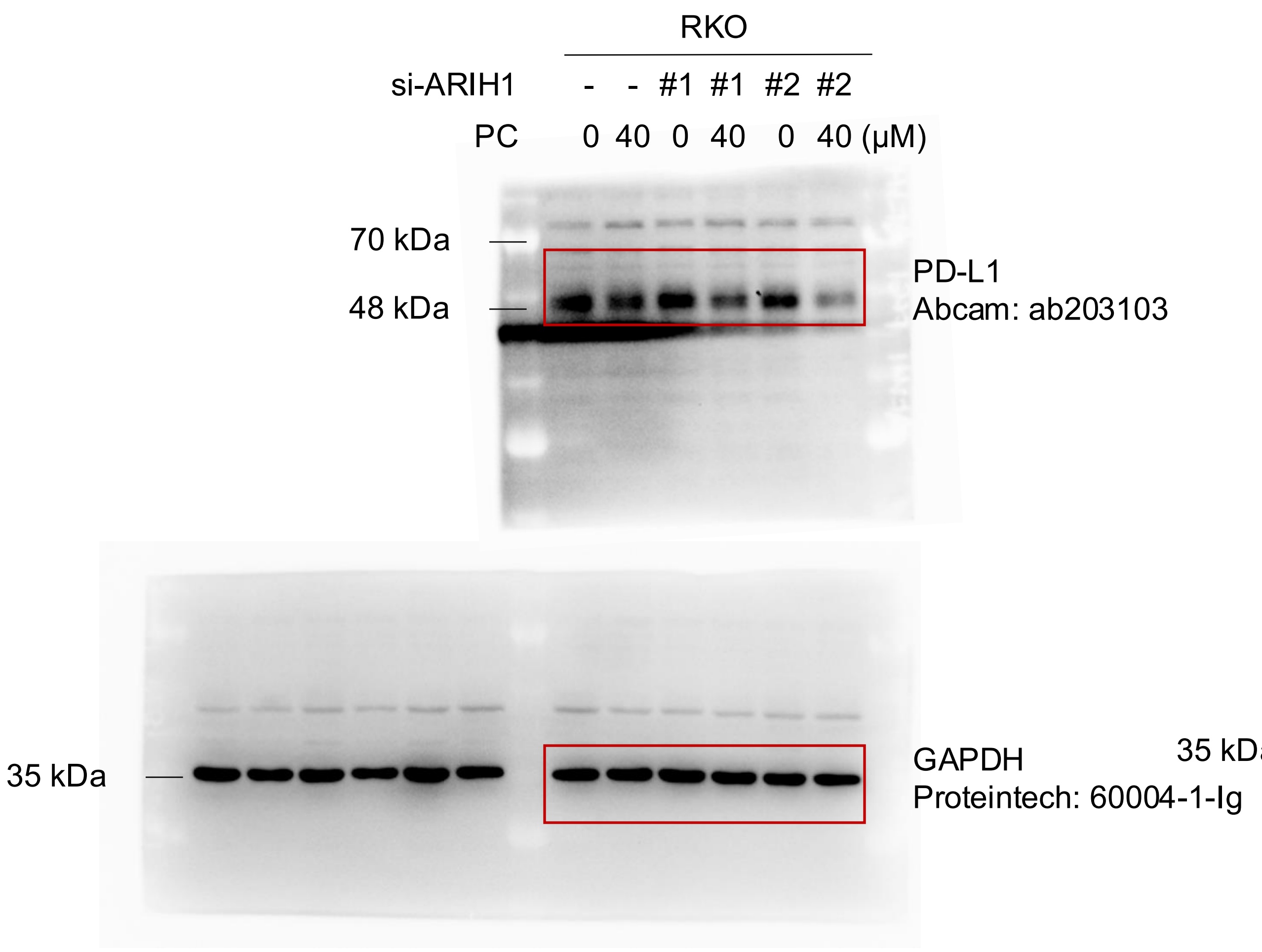

Figure S9B

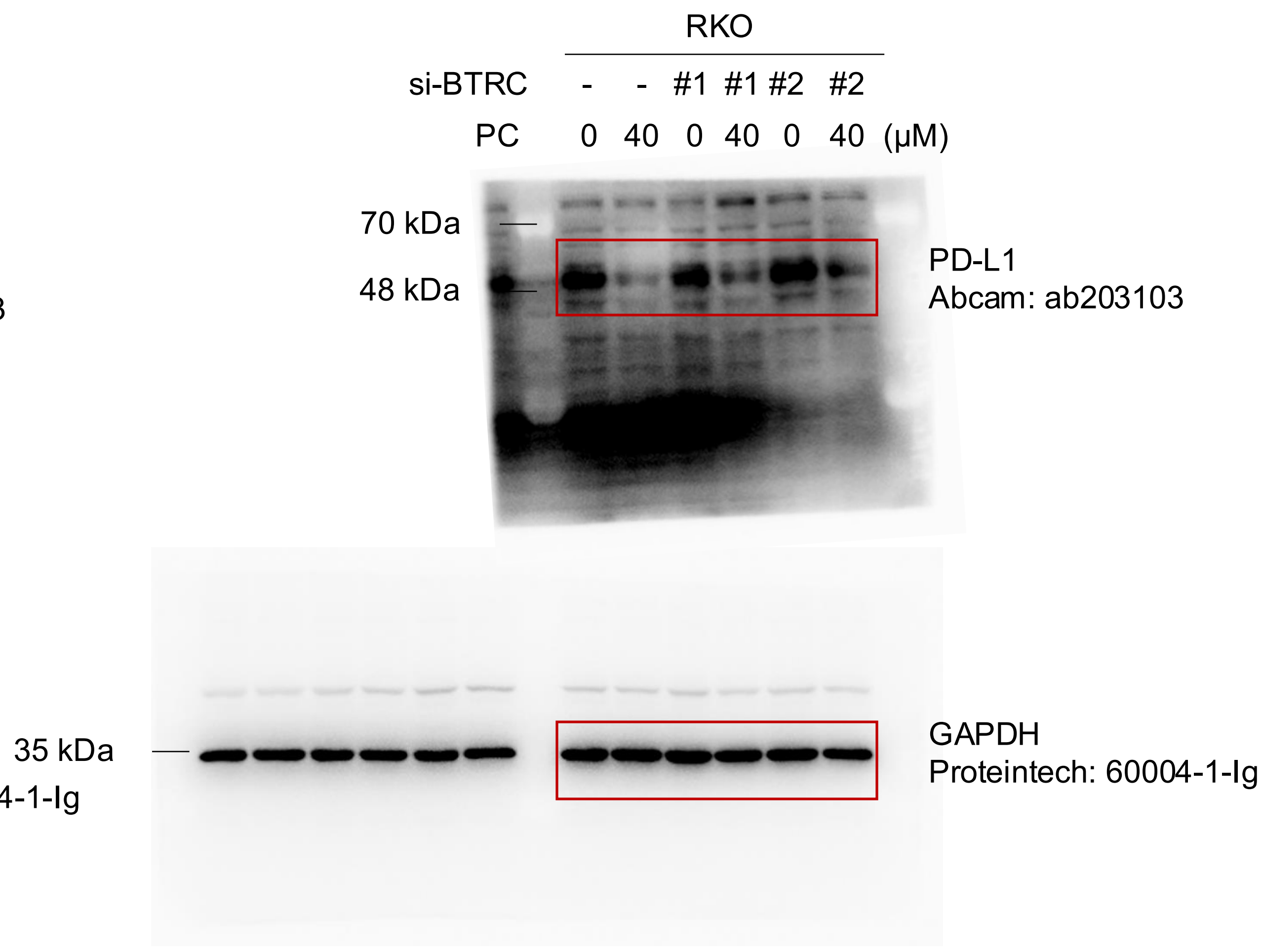

Figure S9C

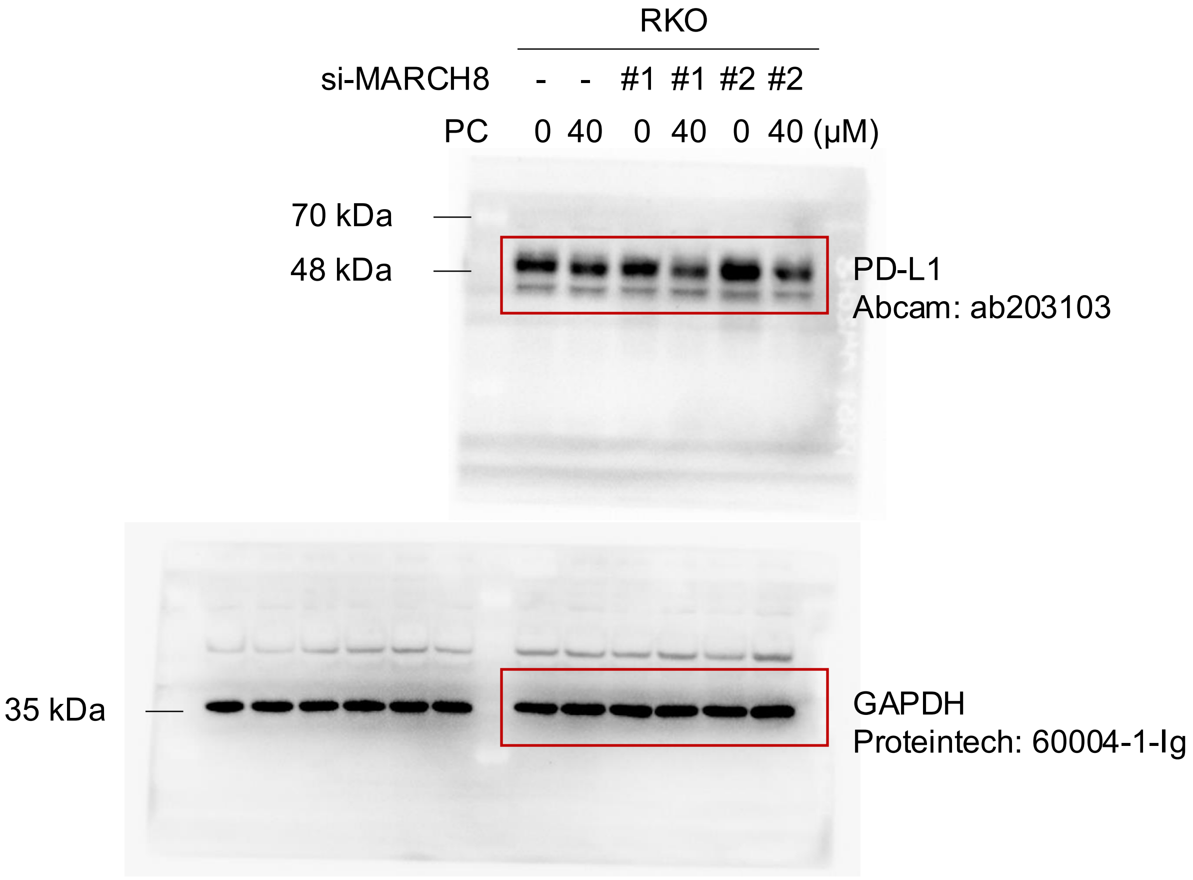

Figure S9D

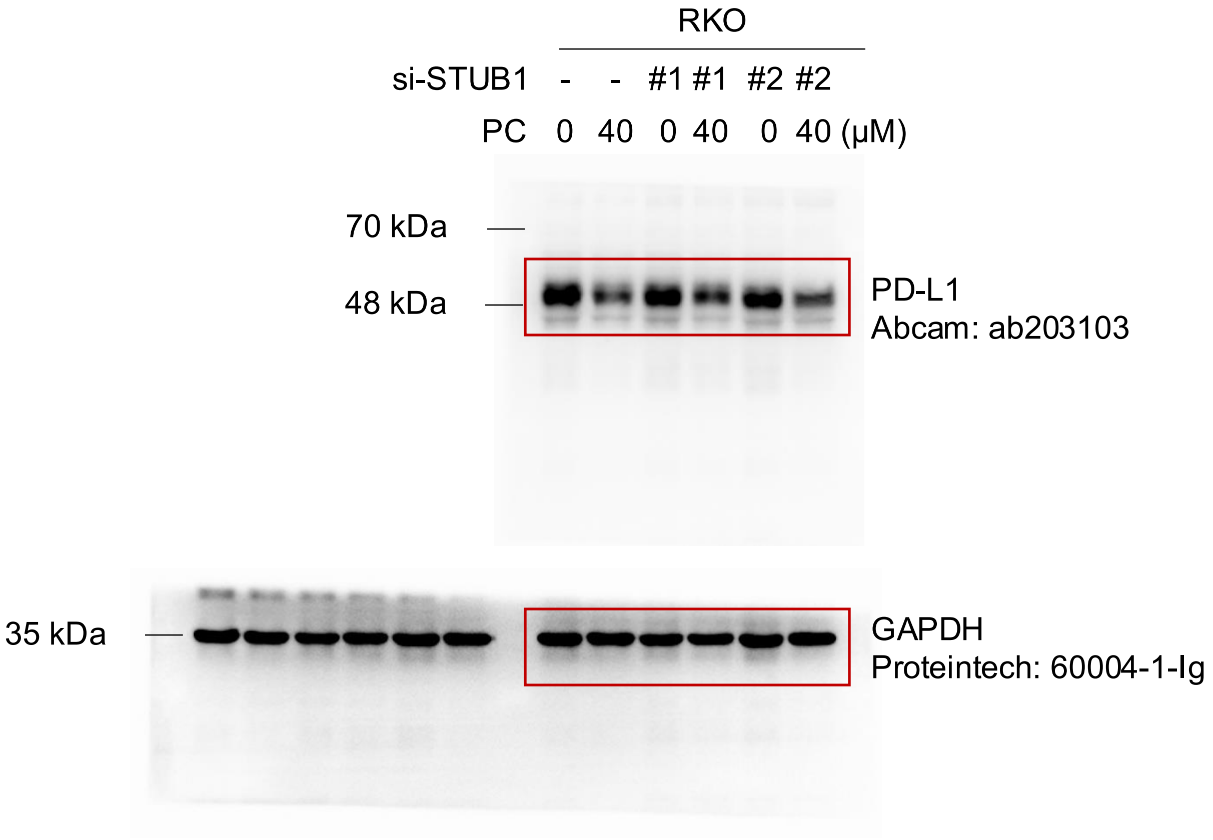

Figure S9E

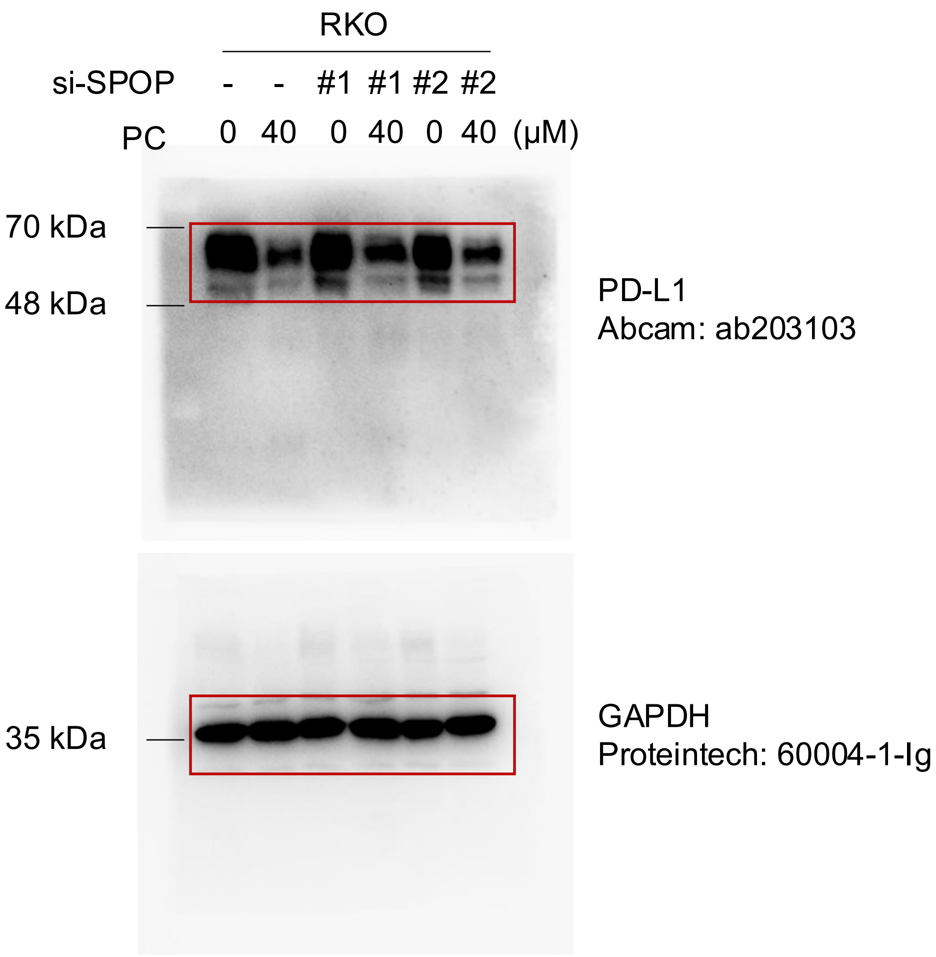

Figure S9F

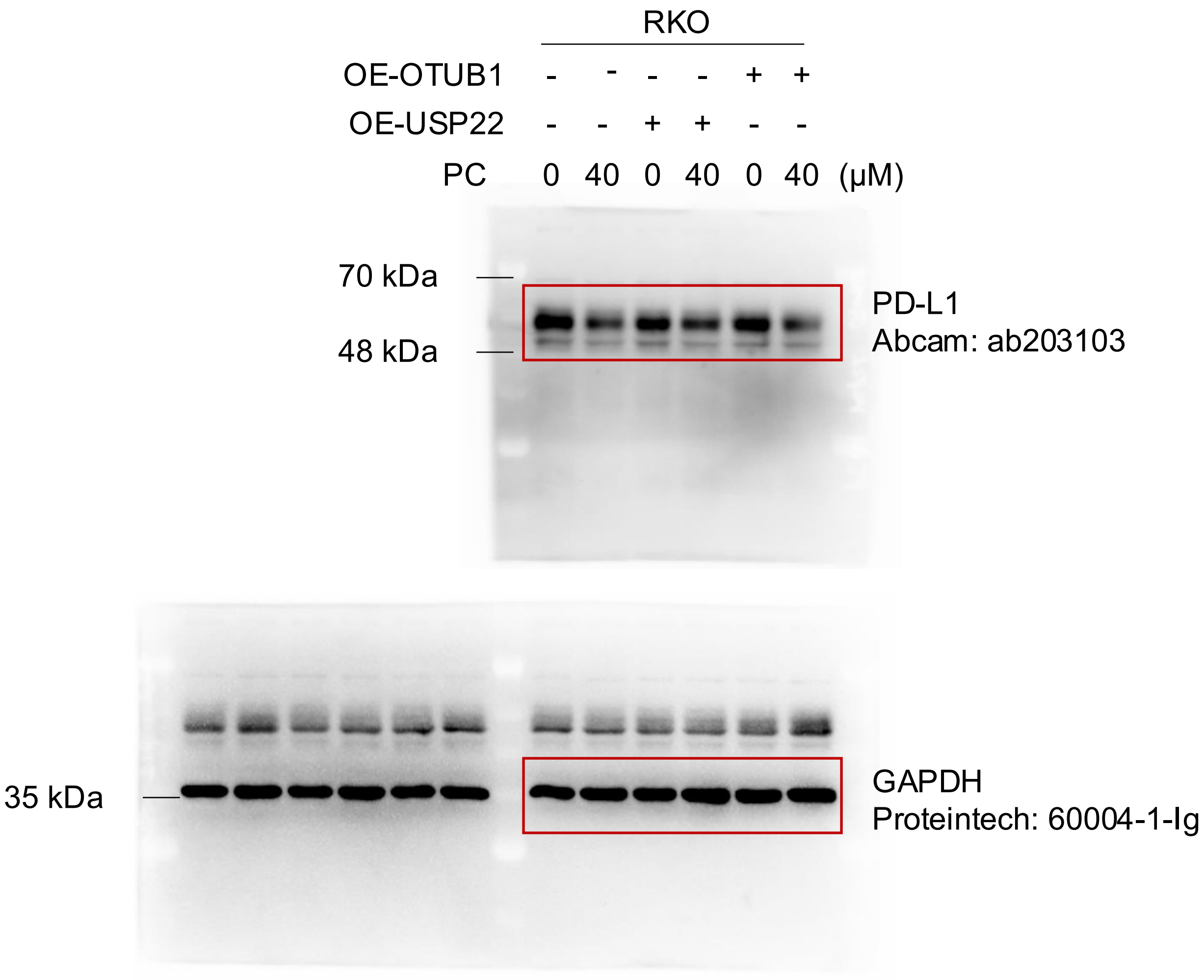

Supplement: Unedited blot and gel images [file jci-136-197592-s034.pdf]
